# ﻿Symbiotic copepods (Cyclopoida and Siphonostomatoida) collected by light trap from Korea

**DOI:** 10.3897/zookeys.1115.83266

**Published:** 2022-07-28

**Authors:** Jimin Lee, Cheon Young Chang, Il-Hoi Kim

**Affiliations:** 1 Marine Ecosystem and Biological Research Center, Korea Institute of Ocean Science & Technology, Busan 49111, Republic of Korea Marine Ecosystem and Biological Research Center, Korea Institute of Ocean Science & Technology Busan Republic of Korea; 2 Department of Biological Science, Daegu University, Gyeongsan 38453, Republic of Korea Daegu University Gyeongsan Republic of Korea; 3 Korea Institute of Coastal Ecology, 302-802, Seokcheon-ro 397, Bucheon 14449, Republic of Korea Korea Institute of Coastal Ecology Bucheon Republic of Korea

**Keywords:** Copepoda, Crustacea, new genus, new species, taxonomy

## Abstract

Thirty-nine species of symbiotic copepods, comprising 24 species of poecilostome Cyclopoida and 15 species of Siphonostomatoida, are reported from Korean waters, which were collected using underwater light traps at 33 collection sites around the South Korean coast. Ten new species are described: *Hemicyclopsrapax***sp. nov.** in the family Clausidiidae; *Pontoclausiacochleata***sp. nov.** and *P.pristina***sp. nov.** in the family Clausiidae; *Heteranthessiusunisetatus***sp. nov.** in the family Lichomolgidae; *Pusanomyicolasensitivus***gen. nov., sp. nov.** in the family Myicolidae; *Polyankylisbogilensis***sp. nov.** in the family Polyankyliidae; *Pseudanthessiuslinguifer***sp. nov.** in the Pseudanthessiidae; *Eupolymniphilusfoliatus***sp. nov.** in the family Sabelliphilidae; and *Acontiophorusestivalis***sp. nov.** and *Thermocherespacificus***sp. nov**. in the family Asterocheridae. Supplementary descriptions or notes for other species are provided as appropriate.

## ﻿Introduction

Light traps are useful tools for collecting marine animals. According to McLeod and Costello (2017), at least 12 phyla of benthic and planktonic animals have been collected in light traps from the world. Animals collected by light traps are unsuitable for strict quantitative research; however, they are often in good condition and well suited for morphological and taxonomic study (Øresland 2007). In some cases, animals seldom taken by other methods can be caught by the light trap (Hernandez Jr. and Shaw 2003; Øresland 2007; Porter et al. 2008).

Crustaceans are the most abundant marine animals that are caught in light traps (Chan et al. 2016). McLeod and Costello (2017) counted 129 marine crustacean species collected in light traps to the date of their study in the world, including 44 copepod species consisting of 29 calanoid species and 15 other species. Holmes (1985) described the fish-parasitic copepod *Anchistrotoslucipetus* as a new species that was collected using a light trap in Irish waters, indicating that strict parasitic animals can be caught in light traps.

Recently copepods caught in light traps have been recorded frequently in Korea (Chang and Song 1995; Chang 2012, 2014; Lee and Chang 2016; Lee et al. 2016; Cho et al. 2018; Jeon et al. 2018, 2019). These copepod species were benthic and planktonic, but not genuinely symbiotic. During the past decade we have collected marine copepods from Korean coasts using a light trap, and the symbiotic copepods (poecilostome Cyclopoida and Siphonostomatoida) are recorded in the present paper.

## ﻿Materials and methods

**Figure 1.**
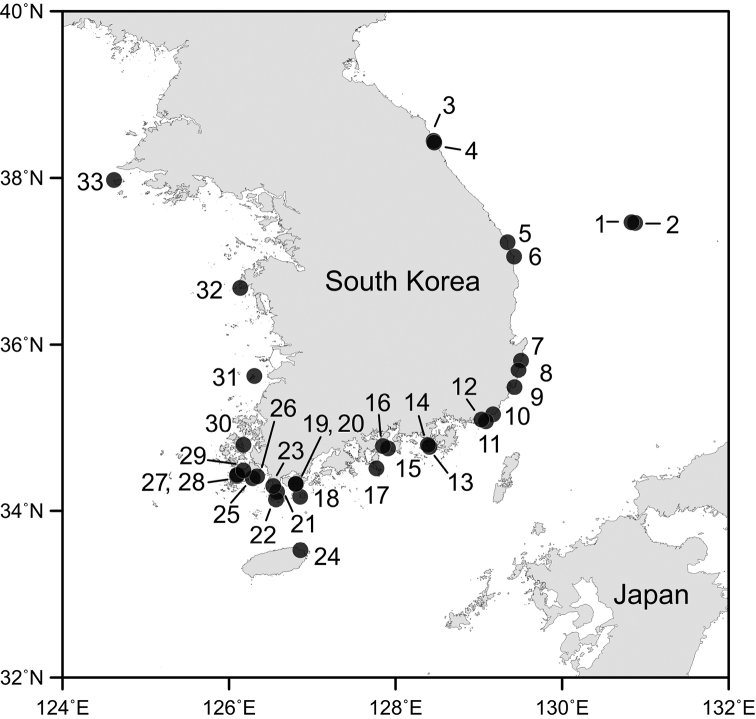
Map showing the collection sites in South Korea.

Copepod specimens in this study were collected using underwater light traps at 33 collection sites (Fig. 1, Table 1) around the South Korean coast during the period from March 2013 to September 2021. Most of these collection sites are fishing ports with water depths less than 10 m. The light traps were made of PVC pipe of ~ 12 cm in diameter and 40 cm long, with a transparent funnel entrance and white-colored LED flashlight inside, as in Sigurdsson et al. (2014). At each collection site, the light trap was deployed on the sea bottom for one or two hours in the early night usually an hour after sunset mainly around the end of the month. Collected material consisting of various invertebrates and fish larvae was fixed with 5% formalin for ~ 1 h and then transferred to 70% ethanol for preservation. The symbiotic copepods were sorted out from the collected material for the present taxonomic study. Before microscopic observation, selected specimens were soaked in lactic acid for ~ ten min. Drawings were made with a drawing apparatus on the microscope. Type specimens have been deposited in the Marine Biodiversity Institute of Korea (**MABIK**), Seocheon, Korea. Scientific names were checked against those in WoRMS (WoRMS Editorial Board 2021).

**Table 1.** Collection sites.

Sites	Localities	Coordinates
1	Sadong, Ulleung I.	37°27'35.7"N, 130°52'34.6"E
2	Namyang, Ulleung I.	37°28'01.3"N, 130°50'01.4"E
3	Geojin, Goseong	38°26'38"N, 128°27'27"E
4	Ban-am, Goseong	38°25'30"N, 128°27'47"E
5	Imwon, Samcheok	37°13'44"N, 129°20'45"E
6	Jukbyeon, Uljin	37°03'22"N, 129°25'22"E
7	Gampo, Gyeongju	35°48'29"N, 129°30'19"E
8	Eupcheon, Gyeongju	35°41'32.6"N, 129°28'30.4"E
9	Bangeojin, Ulsan	35°29'03.9"N, 129°25'44.5"E
10	Haeundae, Pusan	35°09'30"N, 129°10'14"E
11	Yeongdo, Pusan	35°04'31.0"N, 129°05'08.7"E
12	Near Pusan Fish Market	35°05'46"N, 129°01'51"E
13	Minam-ri, Tongyeong	34°46'02.9"N, 128°24'21.1"E
14	Junghwa-ri, Tongyeong	34°47'25.1"N, 128°23'17.9"E
15	Honghyeon-ri, Namhae I.	34°45'00.5"N, 127°54'33.9"E
16	Deogweol, Namhae I	34°46'35.3"N, 127°50'57"E
17	Geum-oh I.	34°30'33.1"N, 127°46'10.1"E
18	Doryak-ri, Cheongsan I.	34°10'12"N, 126°51'13"E
19	Ul-mool, Sinji I.	34°19'25.4"N, 126°48'06.7"E
20	Myeongsa, Sinji I.	34°19'25.48"N, 126°48'05.04"E
21	Nohwa I.	34°13'28"N, 126°53'47"E
22	Yesong, Bogil I.	34°08'11"N, 126°33'49"E
23	Galdu, Haenam	34°17'57"N, 126°31'50"E
24	Saehwa, Jeju I.	33°31'45"N, 126°51'25"E
25	Geumgap-ri, Chindo I.	34°23'30.7"N, 126°17'01.1"E
26	Chopyeong, Chindo I.	34°24'46.3"N, 126°20'11.1"E
27	Saepo, Chindo I.	34°25'10.4"N, 126°05'39.0"E
28	Gahak, Chindo I.	34°25'52.7"N, 126°05'51.4"E
29	Bojeon, Chindo I.	34°29'08.5"N, 126°10'18.5"E
30	Gosan, Palgeum I.	34°47'38"N, 126°10'22"E
31	Wido I.	35°37'04"N, 126°18'15"E
32	Sinjin, Taean	36°40'50"N, 126°08'05"E
33	Dumoojin, Baekryeongdo I.	37°58'31"N, 124°37'10"E

## ﻿Taxonomic account

**Order Cyclopoida Burmeister, 1834**

### ﻿Family Anthessiidae Humes, 1986

#### Genus *Anthessius* Della Valle, 1880

##### ﻿ Anthessius atrinae

Taxon classificationAnimaliaCyclopoidaAnthessiidae

Suh & Choi, 1991

3F59F951-08B2-592B-869D-B3093F12BE19

###### Material examined.

One ♀, Site 12, 16 Mar. 2013.

###### Remarks.

This copepod has been known only from the bivalve *Atrinapectinata* (Linnaeus, 1767). The copepod is presumed to have escaped from its bivalve host in a fish market aquarium into the adjacent waters of the collection site, where the host cannot dwell due to the water pollution.

##### ﻿ Anthessius graciliunguis

Taxon classificationAnimaliaCyclopoidaAnthessiidae

Do & Kajihara, 1984

93A11998-D7D0-5DEF-94BA-14A6B137FF0D

Fig. 2

###### Material examined.

Twenty ♀♀, 9 ♂♂, Site 4, 19 Jul. 2016; 2 ♀♀, Site 5, 21 Jul. 2016; 37 ♀♀, 2 ♂♂, Site 7, 21 Jun. 2019; 7 ♀♀, 3 ♂♂, Site 8, 18 May 2015; 17♀♀, 2 ♂♂, Site 9, 17 May 2015; 8 ♀♀, 2 ♂♂, Site 11, 03 Jun. 2019; 1♀, 1 ♂, Site 11, 07 Jul. 2020; 9 ♀♀, Site 11, 16 Apr. 2014; 10 ♀♀, Site 11, 20 Aug. 2020; 11 ♀♀, Site 12, 16 Mar. 2013; 1♀, 1 ♂, Site 13, 03 Jul. 2020; 9 ♀♀, 1 ♂, Site 14, 03 Jul. 2020; 1 ♀, Site 15, 04 Jul. 2020; 6 ♀♀, Site 16, 04 Jul. 2020; 5 ♀♀, Site 17, 13 May 2015; 24 ♀♀, 4 ♂♂, Site 18, 27 Apr. 2017; 2 ♀♀, Site 19, 05 Jun. 2020; 6 ♀♀, 2 ♂♂, Site 21, 26 May 2017; 62 ♀♀, 13 ♂♂, Site 22, 26 Apr. 2021; 30 ♀♀, 10 ♂♂, Site 22, 31 May 2021; 11 ♀♀, 3 ♂♂, Site 23, 24 Apr. 2021; 3 ♀♀, 1 ♂, Site 26, 06 Jul. 2016; 10 ♀♀, 1 ♂, Site 32, 24 May 2020; 4 ♀♀, 2 ♂♂, Site 33, 11 Aug. 2020.

###### Supplementary description of female.

**Figure 2.**
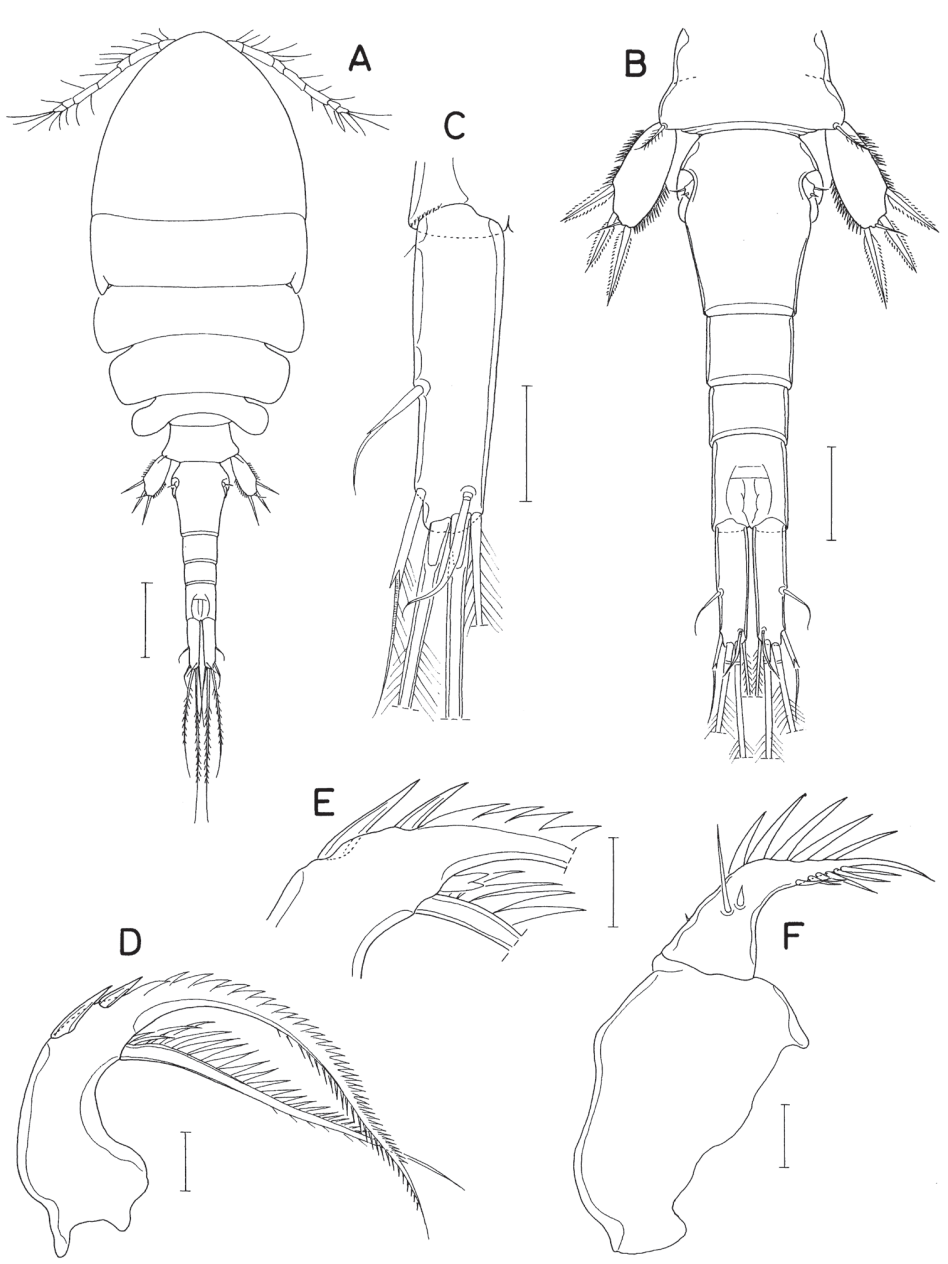
*Anthessiusgraciliunguis* Do & Kajihara, female **A** habitus **B** urosome **C** left caudal ramus, dorsal **D** mandible, dorsal **E** area between inner seta and distal lash of mandible, ventral **F** maxilla. Scale bars: 0.2 mm (**A**); 0.1 mm (**B**); 0.05 mm (**C**); 0.02 mm (**D–F**).

Body (Fig. 2A) narrow. Body length of figured specimen 1.72 mm. Prosome 1.8 × longer than wide (1.06 × 0.59 mm), ~ 60% as long as body length. Cephalothorax with dorsal suture line between cephalosome and first pedigerous somite; posterolateral corners conically produced. Genital double-somite (Fig. 2B) ~ 1.25 × longer than wide (198 × 160 μm), widest at proximal 30% region followed by gradually narrowed distal 70% of double-somite. Caudal ramus (Fig. 2C) 3.49 × longer than wide (136 × 39 μm), gradually narrowed distally, armed with six setae; seta II (outer lateral seta) positioned at 55% of ramus length, with stiff, spiniform proximal half and setiform distal half; seta III (outer distal seta) consisting of distally bifurcate, spiniform proximal part and thin, setiform distal part; seta VII (dorsal seta) annulated proximally, with slightly broadened middle region.

Mandible (Fig. 2D, E) with bifurcate, rudimentary element on ventral side between bases of distal lash and inner seta; inner seta as long as distal lash. Maxilla (Fig. 2F) consisting of syncoxa and basis; basis terminated in spiniform distal lash, armed with three setae (setae I–III); seta I (inner seta) small, rudimentary, positioned close to seta II; seta II simple; seta III minute, almost invisible; distal lash armed with five spines along convex outer margin and six spinules along inner margin.

Leg 4 with three spines and five setae on third exopodal segment. Leg 5 exopod 2.1 × longer than wide.

###### Description.

**Male.** Body form as in female. Body length of measured specimen 1.20 mm.

###### Remarks.

*Anthessiusgraciliunguis* Do & Kajihara, 1984 was described originally as an associate of the mussel *Mytilusgalloprovincialis* Lamarck, 1819 from Japan (Do and Kajihara 1984). Kim (1998, 2010a) recorded four additional bivalve host species in Korea: *Mizuhopectenyessoensis* (Jay, 1857), *Pectenalbicans* (Schröter, 1802), *Scaeochlamyssquamata* (Gmelin, 1791), and *Solecurtusdivaricatus* (Lischke, 1869) in Korea. Ueda et al. (2006) found this copepod species in plankton samples in Japan. In the present study, this copepod occurred most frequently from 19 of 33 collection sites around the coasts of South Korea. Although we have not examined the Korean population of *M.galloprovincialis* for copepods, this mussel seems to be the major host of *A.graciliunguis*, considering that only this mussel inhabits all of those 19 collection sites.

The diagnostic morphological features of the female of *A.graciliunguis* are as follows: (1) the caudal ramus is ~ 3.5 × longer than wide, (2) the terminal segment of antenna is 3.0 × longer than wide; (3) the convex outer margin of the distal lash of maxilla is ornamented with five spines; (4) the third exopodal segment of leg 4 is armed with three spines and five setae; and (5) the exopod of leg 5 is 2.1 × longer than wide. The first (1) and last (5) may be the simple combination of features sufficient to differentiate *A.graciliunguis* from its congeners.

### ﻿Family Clausidiidae Embleton, 1901

#### Genus *Conchyliurus* Bocquet & Stock, 1957

##### ﻿ Conchyliurus quintus

Taxon classificationAnimaliaCyclopoidaClausidiidae

Tanaka, 1961

CA383165-3A09-57F5-BA01-4CFDE1E94A0F

###### Material examined.

One ♂, Site 4, 19 Jul. 2016; 1 ♀, Site 12, 16 Mar. 2013; 1 ♀, Site 33, 11 Aug. 2020.

###### Remarks.

In Korea, *Conchyliurusquintus* is widely distributed along the entire coast. It has a low host specificity, inhabiting 12 species of bivalves in Korea (Kim 2004).

#### Genus *Hemicyclops* Boeck, 1872

##### ﻿ Hemicyclops japonicus

Taxon classificationAnimaliaCyclopoidaClausidiidae

Itoh & Nishida, 1993

BBB9C475-973C-573E-B844-C99D3A6BFFF3

###### Material examined.

Two ♀♀, Site 11, 16 Apr. 2014; 20 ♀♀, 17 ♂♂, Site 22, 31 May 2021; 1 ♀, Site 24, 16 May 2019.

###### Remarks.

This species is easily identifiable due to the characteristic genital double-somite of the female, which has a deep lateral constriction between the anterior third and posterior two-thirds and a pointed process on each lateral margin. The host of this copepod is still unknown.

##### ﻿ Hemicyclops nasutus

Taxon classificationAnimaliaCyclopoidaClausidiidae

Moon & Kim, 2010

94657E38-2ED8-5B57-A550-0B45F53BDA42

###### Material examined.

One ♀, Site 11, 16 Apr. 2014; 1 ♂, Site 20, 05 Jun. 2020; 1 ♀, 7 ♂♂, Site 22, 31 May 2021; 1 ♀, Site 23, 24 Apr. 2021; 1 ♀, 2 ♂♂, Site 27, 09 Jul. 2016.

###### Brief description of male.

Body form as in female. Body length 1.30 mm. Urosome six-segmented. Genital somite wider than long. Caudal ramus 3.03 × longer than wide (115 × 38 μm). Antennule with same armature formula as in female. Antenna, mandible, maxillule the same as those of female. Basis (distal segment) of maxilla terminating in stout claw. Maxilliped four-segmented; first segment (syncoxa) with single large spinulose seta subdistally on inner margin; second segment (basis) broadened proximally, markedly tapering distally, armed with two unequal setae (one spinulose and one minute), and ornamented with three longitudinal rows of denticles along inner margin; small third segment (first endopodal segment) unarmed; terminal segment forming long, curved claw bearing two setae proximally.

Leg 1 different from that of female in absence of inner distal spine on basis. Legs 2–4 as in female. Leg 5 consisting of single dorsolateral seta on fifth pedigerous somite and exopod; protopod completely fused with somite. Leg 6 represented by one spine on posterolateral corner of genital operculum.

###### Remarks.

Moon and Kim (2010) described this species based on a single female found on an unidentified polychaete from the Yellow Sea, Korea. The male is recorded here for the first time.

##### ﻿ Hemicyclops rapax sp. nov.

Taxon classificationAnimaliaCyclopoidaClausidiidae

23EAFB58-675D-59A0-BCE5-8CA97449FDF7

https://zoobank.org/9FE3EF15-2C7B-4266-B619-190EAEE13FC8

Figs 3 , 4 , 5 , 6

###### Material examined.

***Holotype*** ♀ (MABIK CR00250118) and ***paratype*** ♀ (MABIK CR00250119) preserved in 90% alcohol, Site 22 (Yesong, Bogil Island, south coast, 34°08'11"N, 126°33'48"E), 31 May 2021, leg. J. Lee; paratype ♂ (MABIK CR00250123, figured) dissected and mounted on a slide, Site 22, 26 April 2021, leg. J. Lee and C. Y. Chang; 1 ♀ preserved in 90% alcohol and 1 ♀ (figured) dissected and mounted on a slide, Site 11 (Yeongdo, Pusan, 35°04'31.0"N, 129°05'08.7"E), 07 Jul. 2020, leg. J. G. Kim.

###### Description.

**Figure 3.**
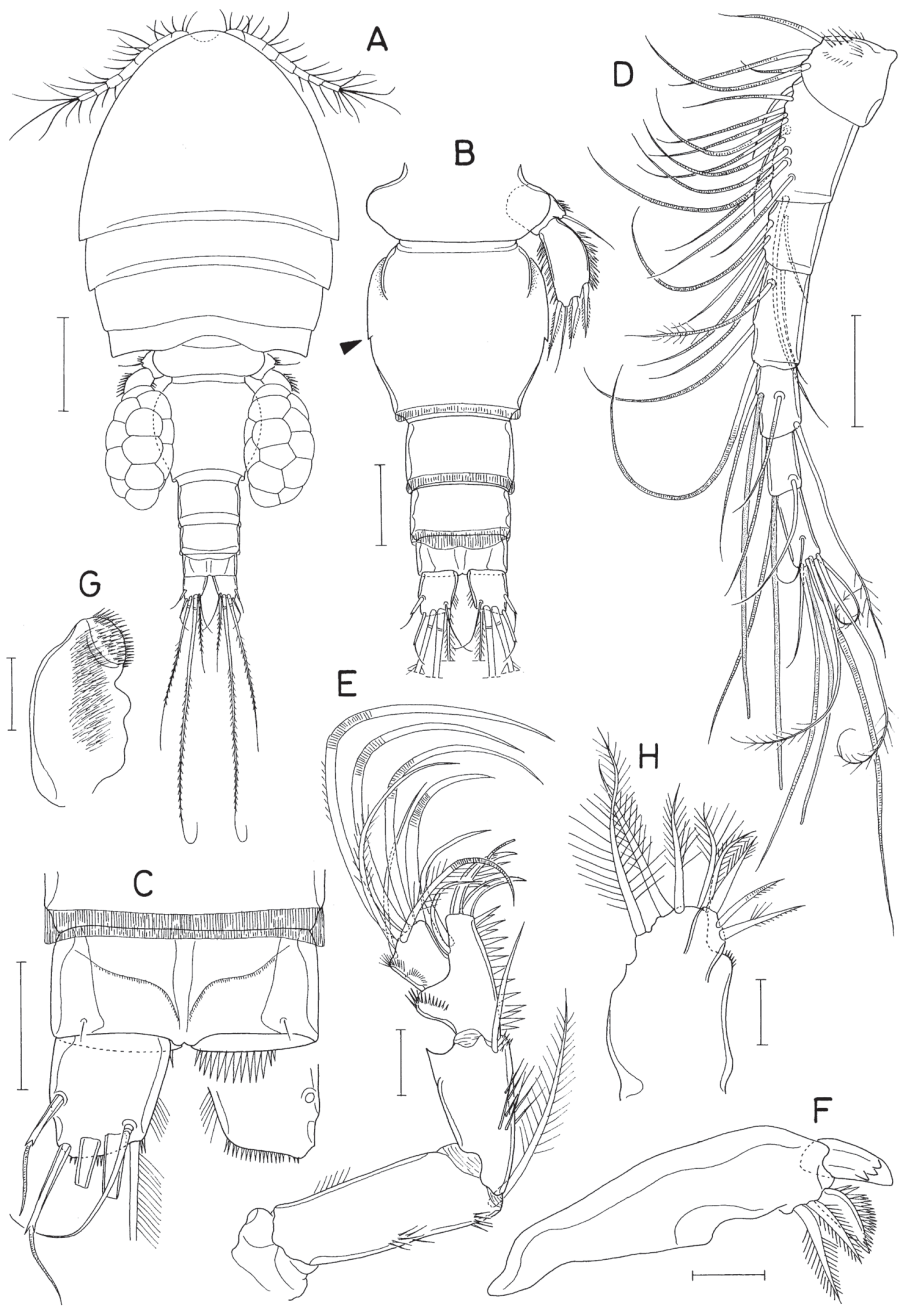
*Hemicyclopsrapax* sp. nov., female **A** habitus, dorsal **B** urosome, dorsal (arrowhead indicates a small denticle on lateral margin of genital double-somite) **C** anal somite and caudal rami, dorsal **D** antennule **E** antenna **F** mandible **G** paragnath **H** maxillule. Scale bars: 0.2 mm (**A**); 0.1 mm (**B**); 0.05 mm (**C, D**); 0.02 mm (**E–H**).

**Female.** Body (Fig. 3A) moderately stout, dorsoventrally flattened. Body length 1.10 mm in dissected and figured specimen (1.22 mm in holotype). Prosome 680 × 555 μm, ~ 56% as long as body length. Posterolateral corners of all prosomal somites pointed or angular. Urosome (Fig. 3B) five-segmented. Fifth pedigerous somite 234 μm wide, slightly wider than genital double-somite. Genital double-somite subcircular, flattened ventrally, thin laterally, slightly wider than long (220 × 227 μm) in dissected specimen or slightly longer than wide in smaller other specimens; lateral margin with small denticle (this denticle absent smaller specimens) as indicated by arrowhead in Fig. 3B; genital apertures positioned dorsolaterally at anterior part of double-somite. Three abdominal somites 84 × 136 μm, 61 × 127 μm, and 45 × 114 μm, respectively. Genital double-somite and first two free abdominal somites with membranous fringe along posterior margin. Anal somite with row of spinules along posteroventral margin. Caudal ramus (Fig. 3C) short, 1.15 × longer than wide (55 × 48 μm), slightly longer than anal somite, armed with six setae (setae II-VII), and ornamented with setules on distal half of inner margin and fine spinules along posteroventral margin; seta II (outer lateral seta) positioned at midlength of ramus, spiniform in proximal half but setiform in distal half; seta III (outer distal seta) also proximally spiniform and distally setiform; dorsal seta (seta VII) annulated proximally; setae II, III, and VII naked, but other three setae pinnate.

Rostrum small, with convex posterior margin. Antennule (Fig. 3D) 237 μm long, seven-segmented; armature formula 4, 14, 6, 3, 4+aesthetasc, 2+aesthetasc, and 7+aesthetasc; first segment ornamented with fines setules on anterior surface; setae and aesthetascs slender; most setae naked except single on each fourth and fifth segments and three on terminal segment. Antenna (Fig. 3E) consisting of coxa, basis and three-segmented endopod; armature formula 0, 1, 1, 4, and 7; basis ornamented with two patches of spinules on inner margin and several setules on outer margin; first endopodal segment with acutely pointed outer distal corner and patch of spinules on inner surface and patch of small spinules at inner distal corner; second endopodal segment with prominent inner distal prolongation (this prolongation distinctly longer than wide; its two apical setae strong, claw-like), ornamented with broad spinules along inner margin and row of small spinules near outer distal corner; third endopodal segment as long as wide, ornamented with two rows of minute setules on outer side; four of seven setae on third endopodal segment claw-like, wrinkled in middle.

**Figure 4.**
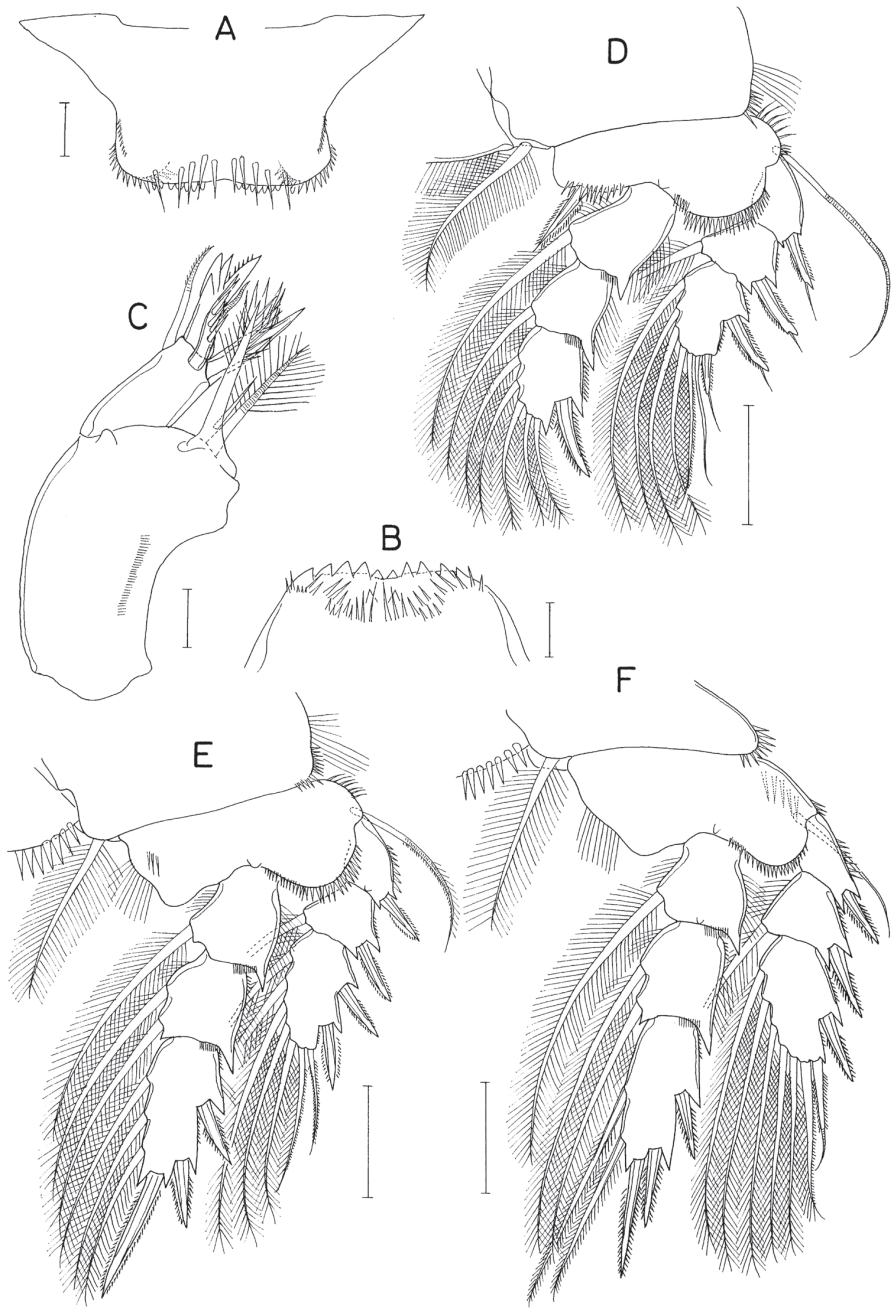
*Hemicyclopsrapax* sp. nov., female **A** labrum **B** labium **C** maxilla **D** leg 1 **E** leg 2 **F** leg 4. Scale bars: 0.02 mm (**A–C**); 0.05 mm (**D–F**).

**Figure 5.**
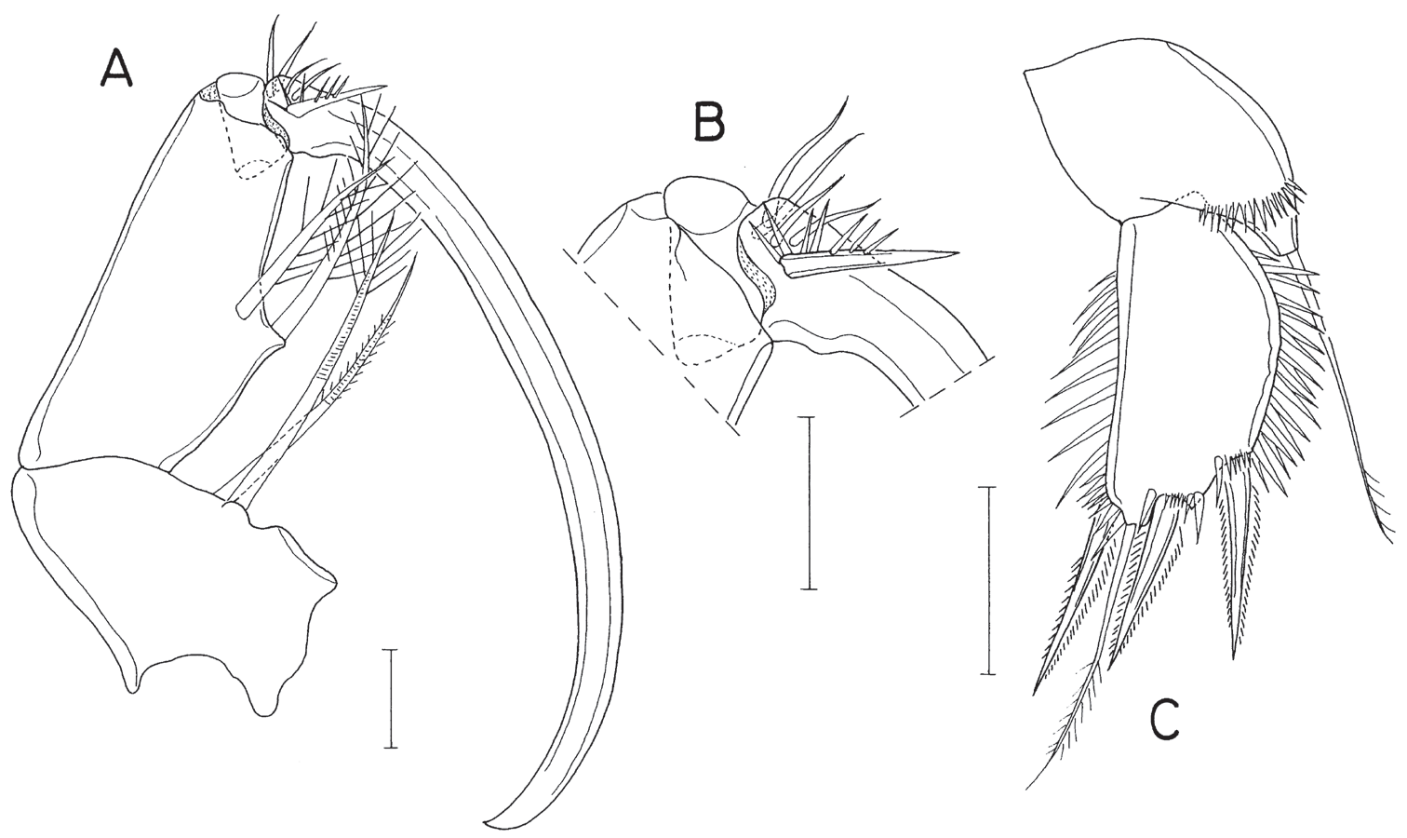
*Hemicyclopsrapax* sp. nov., female **A** maxilliped **B** distal part of maxilliped **C** leg 5. Scale bars: 0.02 mm (**A, B**); 0.05 mm (**C**).

Labrum (Fig. 4A) with denticles and spinules on posterior margin. Labium (Fig. 4B) denticulated along anterior margin, spinulose subapically. Mandible (Fig. 3F) distally armed with one stout, denticulate element, one spinulose, plate-like element and two pinnate setae. Paragnath (Fig. 3G) lobate, ornamented with fine setules on middle and subdistal regions and spinules on distal region, with trace of articulation subdistally. Maxillule (Fig. 3H) unequally bilobed distally; smaller inner lobe armed with three weakly pinnate setae; larger outer lobe with five pinnate setae. Maxilla (Fig. 4C) two-segmented; proximal segment (syncoxa) armed with three setae, smallest one setule-like, inserted on proximal region of spiniform largest seta, and ornamented with row of minute spinules on proximal region; distal segment (basis) distally armed with three heavily spinulose or denticulate spines and single seta. Maxilliped (Fig. 5A) four-segmented; first segment (syncoxa) with two large setae on inner margin; second segment (basis) also with two large setae on inner margin; third segment (first endopodal segment) short, unarmed; terminal segment (second endopodal segment) forming large hook (this hook much longer than proximal three segments), proximally armed with one spine bearing seven spinules on outer margin and four small, naked setae (Fig. 5B).

Legs 1–4 biramous, with three-segmented rami (Fig. 4D–F); basis spinulose along outer margin and posterior margin between bases of rami. Intercoxal plate setulose in leg 1 (Fig. 4D) but spinulose in legs 2–4 (Fig. 4E, F). Outer spines on exopod of leg 1 tipped with setule. Leg 3 armed and shaped as leg 2. Two inner setae on third endopodal segment of leg 4 stiff, spiniform. Armature formula for legs 1–4 as follows:

**Table:** 

	Coxa	Basis	Exopod	Endopod
Leg 1	0-1	1-I	I-0; I-1; II, 6	0-1; 0-1; I, 5
Legs 2 & 3	0-1	1-0	I-0; I-1; II, 7	0-1; 0-2; I, II, 3
Leg 4	0-1	1-0	I-0; I-1; II, 6	0-1; 0-2; I, II, 2

Leg 5 (Fig. 5C) two-segmented; proximal segment (protopod) with one slender outer seta and row of spinules at outer distal region; distal segment (exopod) 1.95 × longer than wide (86 × 44 μm) densely ornamented with spinules along outer and inner margins, armed with three spines and one weakly pinnate seta; lengths of three spines 54, 50, and 56 μm respectively from outer to inner. Leg 6 invisible.

**Figure 6.**
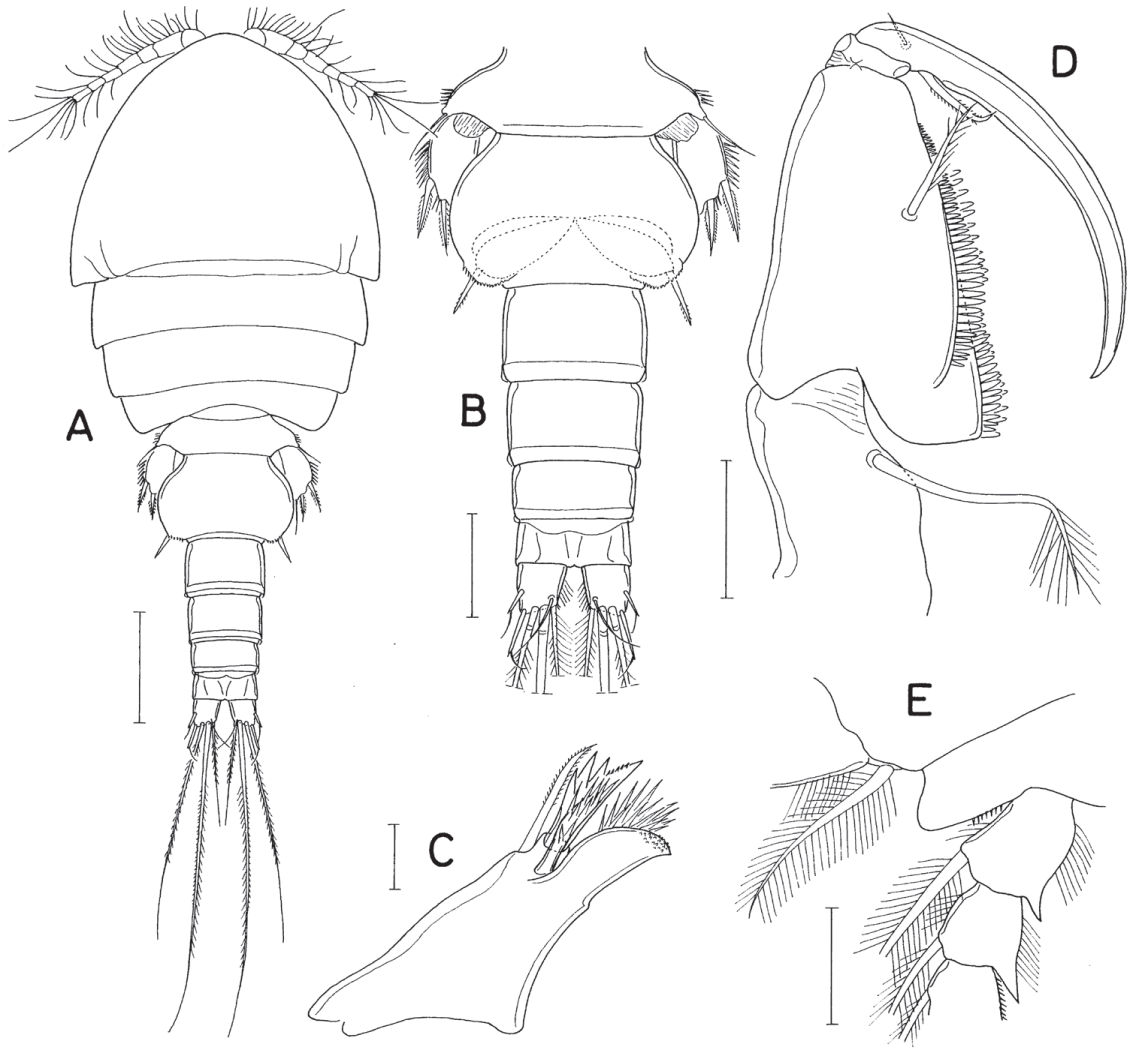
*Hemicyclopsrapax* sp. nov., male **A** habitus, dorsal **B** urosome, dorsal **C** distal segment of maxilla **D** maxilliped **E** inner proximal region of leg 1. Scale bars: 0.2 mm (**A**); 0.1 mm (**B**); 0.02 mm (**C**); 0.05 mm (**D, E**).

**Male.** Body (Fig. 6A) slightly larger than that of female. Body length 1.28 mm. Cephalothorax distinctly broader than next somites. Posterolateral corners of all prosomal somites blunt or rounded. Urosome (Fig. 6B) six-segmented. Fifth pedigerous somite with membranous flap on each side of posterodorsal margin. Genital somite much wider than long (152 × 255 μm), with finely serrate posterodorsal corners and single spine on genital operculum. All urosomal somites lacking membranous fringe on posterior margin. Four abdominal somites gradually shorter from proximal to distal. Caudal ramus 1.04 × longer than wide (50 × 48 μm).

Rostrum as in female. Antennule different from that of female in having one additional seta at proximal anterior margin of fourth segment (thus with 4 setae on this segment). Antenna, labrum, mandible, paragnath, maxillule as in female. Maxilla different from that of female; basis armed with two spines and one seta and terminating in stout, claw-like process bearing spinules on outer margin and granule-like papillae on distal region (Fig. 6C). Maxilliped (Fig. 6D) four-segmented; first segment (syncoxa) with one large, distally pinnate seta; second segment (basis) strongly tapering distally, armed with one pinnate seta, ornamented with three rows of denticles along inner margin; small third segment (first endopodal segment) unarmed; terminal segment as hook bearing two unequal setae proximally.

Leg 1 different from that of female in absence of inner distal element on basis (Fig. 6E). Legs 2–4 as in female. Leg 5 consisting of one naked dorsolateral seta on somite (protopod completely fused with somite) and exopod; exopod shaped and armed as in female. Leg 6 represented by single spine on genital operculum (Fig. 6B).

###### Etymology.

The specific name *rapax* is derived from the Latin *rapa* (grasping), alluding to the grasping form of the female maxilliped.

###### Remarks.

*Hemicyclopsrapax* sp. nov. is characterized by its peculiar female maxilliped, in which the terminal segment is transformed to a large hook, as in the males of existing species. This form of female maxilliped is very unusual for the genus, since the terminal segment (second endopodal segment) of the female maxilliped in other species of the genus generally terminates in a spiniform process (or a spine). The only other example of this peculiar female maxilliped in *Hemicyclops* is that of *H.cylindraceus* (Pelseneer, 1929), as illustrated by Stock (1954), although the terminal hook is much less developed in the latter species. Otherwise, *H.cylindraceus* differs from *H.rapax* sp. nov. in having a narrow, almost cylindrical body, five setae on the first antennular segment, four setae (without spines) on the exopod of leg 5, and an inner distal element on the basis of leg 1 (Stock 1954).

*Hemicyclopsrapax* sp. nov. may be differentiated from its congeners in other ways. In eight species in *Hemicyclops* the caudal ramus is short, less than 1.5 × longer than wide in the female, as in *H.rapax* sp. nov. In five of these eight species (*H.apiculus* Humes, 1995, *H.australis* Nicholls, 1944, *H.intermedius* Ummerkutty, 1962, *H.parapiculus* Kim & Hong, 2014, and *H.vicinalis* Humes, 1995), the genital double-somite of the female is distinctly longer than wide (more than 1.2 × longer than wide); in *H.tamilensis* (Thompson & T. Scott, 1903) the urosome of the female is six-segmented and the exopod of leg 5 is elongated; in *H.saxatilis* Ho & Kim, 1992 the basis of male leg 1 bears an inner distal spine, the first segment of the male maxilliped is armed with two (rather than one) setae, and the maxilla is not sexually dimorphic. In the remaining species, *H.leggii* (Thompson & T. Scott, 1903) described based on the male, the third endopodal segment is armed with five armature elements (I, 4, rather than I, 5), the basis of male leg 1 is armed with an inner distal spine, and the first segment of male maxilliped is armed with two setae. These differences are considered sufficient to distinguish the new species from these eight congeners.

##### ﻿ Hemicyclops parilis

Taxon classificationAnimaliaCyclopoidaClausidiidae

Moon & Kim, 2010

FA51A4C1-C5D2-5B62-A67C-519676627FD8

###### Material examined.

Two ♀♀, Site 11, 20 Aug. 2020; 1 ♀, Site 12, 16 Mar. 2013; 2 ♀♀, 1 ♂, Site 15, 04 Jul. 2020; 1 ♀, Site 20, 05 Jun. 2020; 2 ♀♀, 8 ♂♂, Site 26, 06 Jul. 2016; 1 ♂, Site 33, 11 Aug. 2020.

###### Remarks.

Due to the close relatedness of this species to *H.gomsoensis* Ho & Kim, 1992, Moon and Kim (2010) compared it in detail with the latter species in the original description. Moon and Kim (2010) found *H.parilis* from burrows of unknown invertebrates on the south coast of Korea. The host of this copepod has turned out to be the decapod crustacean *Upogebiaissaeffi* (Balss, 1913). We collected it at six collection sites in this study, which indicates that the decapod host is likely to occur at those sites.

#### Genus *Hersiliodes* Canu, 1888

##### ﻿ Hersiliodes exiguus

Taxon classificationAnimaliaCyclopoidaClausidiidae

Kim & Stock, 1996

B3028D0B-8E5F-52A0-80F5-93B942E75659

###### Material examined.

One ♀, Site 1, 28 Jun. 2021; 1 ♀, Site 22, 31 May 2021.

###### Remarks.

This is the second record of *Hersiliodesexiguus* which was originally recorded as an associate of the clam *Ruditapesphilippinarum* (A. Adams & Reeve, 1850) inhabiting a brackish lagoon on the east coast of Korea (Kim and Stock 1996). The characteristic form of the female genital double-somite bearing a pair of lateral projections allows easy identification of this species without dissection.

### ﻿Family Clausiidae Giesbrecht, 1895

#### Genus *Pontoclausia* Bacescu & Por, 1957

##### ﻿ Pontoclausia cochleata sp. nov.

Taxon classificationAnimaliaCyclopoidaClausiidae

D71C474D-4D07-5977-BB6D-7E2A89FDE6E1

https://zoobank.org/0B9BBD7E-FB2C-42ED-B5BB-4DD3BF0927EA

Figs 7 , 8 , 9

###### Material examined.

***Holotype*** ♀ (MABIK CR00250124) dissected and mounted on a slide, Site 22 (Yesong, Bogil Island, south coast, 34°08'11"N, 126°33'49"E), 26 Apr. 2021, leg. J. Lee and C. Y. Chang; ***Paratype*** ♂ (MABIK CR00250125) dissected and mounted on a slide, Site 27 (Sepo, Chindo Island, southwest coast, 34°25'10.4"N, 126°05'39.0"E), 09 Jul. 2016, leg. J. Lee and C. Y. Chang.

###### Description.

**Figure 7.**
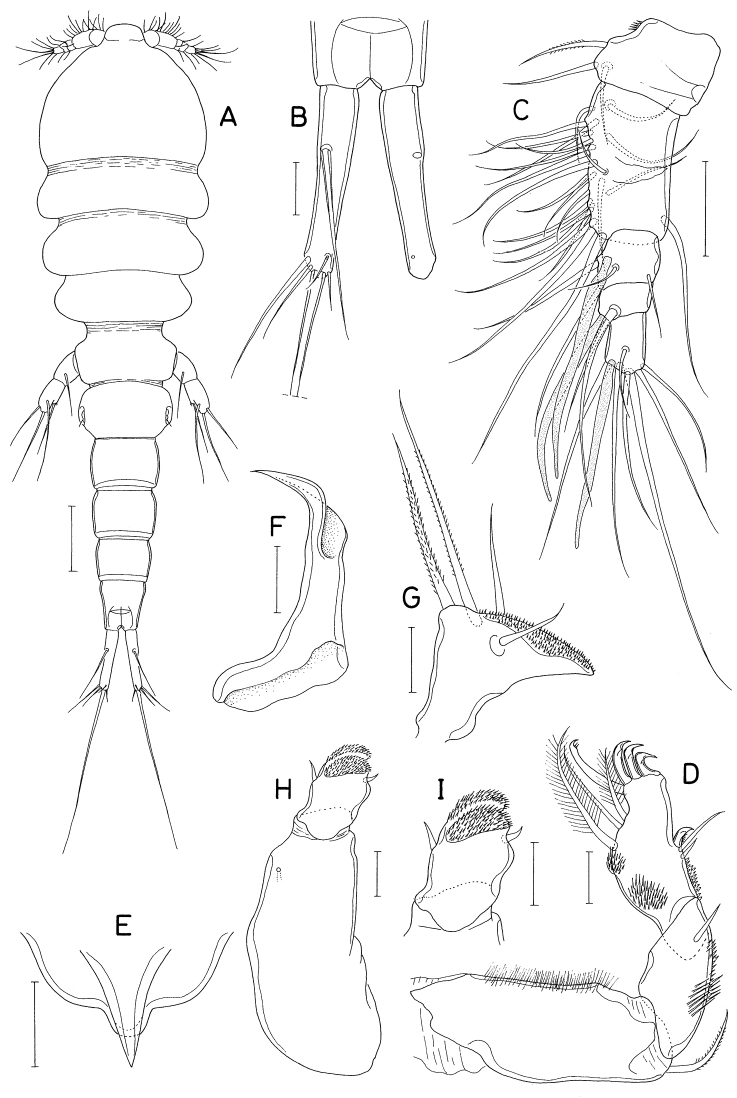
*Pontoclausiacochleata* sp. nov., female **A** habitus, dorsal **B** caudal rami, dorsal **C** antennule **D** antenna **E** labrum **F** mandible **G** maxillule **H** maxilla **I** distal segment of maxilla. Scale bars: 0.2 mm (**A**); 0.05 mm (**B, C**); 0.02 mm (**D–I**).

**Female.** Body (Fig. 7A) narrow, gradually narrowing from anterior to posterior. Body length 2.10 mm. Maximum width 523 μm across cephalothorax. Prosome 936 μm long, shorter than urosome, consisting of cephalothorax and second to fourth pedigerous somites. Cephalothorax wider than long, without dorsal suture line between cephalosome and first pedigerous somite. All prosomal somites with rounded lateral margins. Urosome six-segmented. Fifth pedigerous somite 340 μm wide. Genital somite ~ 1.8 × wider than long (170 × 304 μm), with convex lateral margins; genital aperture positioned dorsolaterally near middle of somite. Four abdominal somites unornamented, 160 × 220 μm, 152 × 200 μm, 130 × 174 μm, and 148 × 144 μm, respectively. Anal somite tapering distally. Caudal rami (Fig. 7B) divergent; each ramus 4.9 × longer than wide (186 × 38 μm), gradually narrowed distally, armed with six stiff, naked setae (setae II–VII); seta II as long as ramus, positioned dorsolaterally at 34% region of ramus length; setae III to VII 136 μm, 83 μm, 532 μm, 38 μm, and 33 μm long, respectively; seta V much larger than other caudal setae, nearly 3.0 × longer than ramus.

Rostrum represented by spatulate anterior prominence of cephalothorax (Fig. 7A). Antennule (Fig. 7C) short, 190 μm long, five-segmented; armature formula 3, 24, 4+aesthetasc, 2+aesthetasc, and 7+aesthetasc; all setae naked; aesthetascs tapering in distal part; first segment with few minute spinules on proximal anterior margin. Antenna (Fig. 7D) three-segmented; first segment (coxobasis) longest, with one seta at inner distal corner and hair-like setules on outer margin; second segment (first endopodal segment) with one seta subdistally and two groups of spinules on inner surface; third segment (fused second and third endopodal segments) armed with one claw plus two setae on inner margin, four claws distally (outermost claw longest, bearing two minute spinules subdistally on inner margin), three setae on subdistal outer margin (middle one naked, but other two pinnate), and ornamented with three patches of spinules (two patches on outer side and one on proximal inner margin).

**Figure 8.**
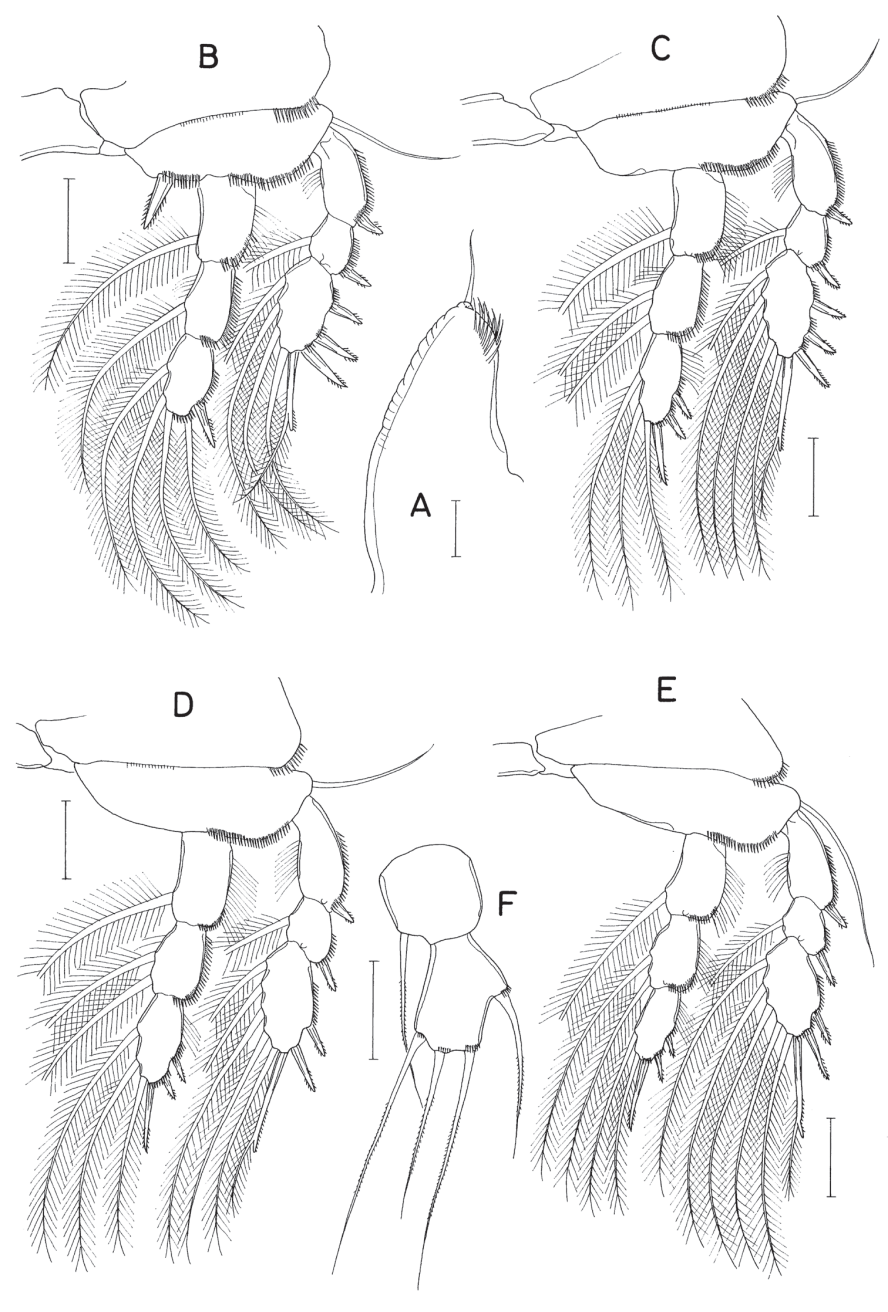
*Pontoclausiacochleata* sp. nov., female **A** maxilliped **B** leg 1 **C** leg 2 **D** leg 3 **E** leg 4 **F** leg 5. Scale bars: 0.02 mm (**A**); 0.05 mm (**B–E**); 0.1 mm (**F**).

Labrum (Fig. 7E) small, not covering mouthparts, with protuberance in middle of posterior margin and large, tapering, beak-like process on dorsal surface. Mandible (Fig. 7F) unarmed but highly transformed; its distal part curved, tapering, scoop-like. Maxillule (Fig. 7G) as foot-like lobe, distally expanded medially, armed with four setae (two outer ones longer than other two); broadened distal surface covered with numerous spinules. Maxilla (Fig. 7H) two-segmented; proximal segment (syncoxa) unarmed, ~ twice longer than wide; distal segment (basis; Fig. 7I) blunt, with two spinulose pads apically, armed with two small subdistal setae each on inner and outer margins. Maxilliped (Fig. 8A) as unsegmented, tapering lobe tipped with one naked seta, ornamented with two subapical rows of setules (or spinules).

Legs 1–4 (Fig. 8B–E) biramous, with three-segmented rami; both rami of each leg slender, almost equal in length. Coxa lacking inner seta but ornamented with spinules on outer distal corner. Basis with spinules on distal margin between rami; outer seta long, naked. Terminal spine on third exopodal segment of legs 1–4 characteristically unequally bifurcate at tip. Armature formula for legs 1–4 as follows:

**Table:** 

	Coxa	Basis	Exopod	Endopod
Leg 1	0-0	1-I	I-0; I-1; III, I, 4	0-1; 0-1; I, 2, 2
Leg 2	0-0	1-0	I-0; I-1; III, I, 5	0-1; 0-2; II, I, 3
Leg 3	0-0	1-0	I-0; I-1; II, I, 5	0-1; 0-2; II, I, 3
Leg 4	0-0	1-0	I-0; I-1; II, I, 5	0-1; 0-2; II, I, 2

Leg 5 (Fig. 8F) two-segmented; proximal segment (protopod) articulated from somite, armed with one dorsodistal seta of 180 μm long. Distal segment (exopod) 1.4 × longer than wide (114 × 82 μm), armed with four slender setae, single on inner margin and three on distal margin; inner margin seta 170 μm long; three distal setae 252, 180, and 261 μm long respectively from inner to outer. All setae on leg 5 finely spinulose (or with minute setules). Leg 6 probably represented by single minute seta on genital operculum.

**Figure 9.**
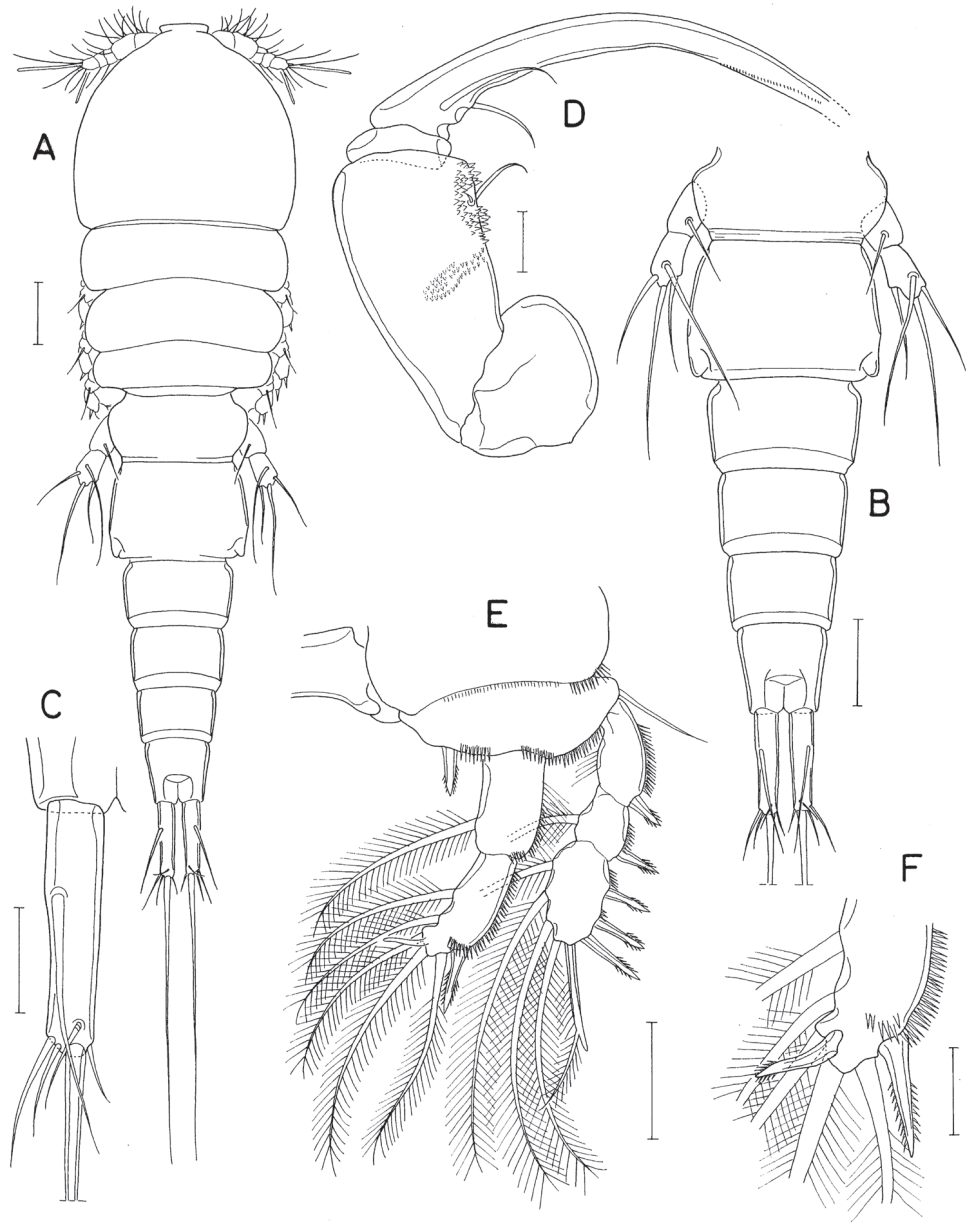
*Pontoclausiacochleata* sp. nov., male **A** habitus, dorsal **B** urosome, dorsal **C** left caudal ramus, dorsal **D** maxilliped **E** leg 1 **F** distal part of endopod of leg 1. Scale bars: 0.1 mm (**A**); 0.05 mm (**B, C**); 0.02 mm (**D, F**); 0.05 mm (**E**).

**Male.** Body form (Fig. 9A) as in female. Body length 1.36 mm. Urosome (Fig. 9B) six-segmented, as in female. Genital somite rectangular, wider than long (158 × 224 μm), as wide as fifth pedigerous somite, gradually broadened distally; genital opercula indistinct, positioned at outer distal corners. Four abdominal somites 106 × 178 μm, 97 × 150 μm, 82 × 127 μm, 97 × 103 μm, respectively. Caudal ramus (Fig. 9C) 4.32 × longer than wide (121 × 28 μm), armed as in female.

Rostrum as in female. Antennule and antenna segmented and armed as in female. Labrum, mandible, maxillule, and maxilla also as in female. Maxilliped (Fig. 9D) four-segmented; first segment (syncoxa) wider than long, unarmed; second segment (basis) gradually broadened distally, armed with two unequal setae, one of them rudimentary, on subdistal inner margin, ornamented with two patches of scale-like spinules; short third segment (first endopodal segment) unarmed; terminal segment as long, arched hook bearing two simple setae proximally.

Leg 1 (Fig. 9E) with three-segmented exopod and two-segmented endopod; compound distal endopodal segment (Fig. 9F) armed with two spines plus five setae (formula I, 2, I, 3). Legs 2–4 as in female. Leg 5 also as in female; exopodal segment 1.5 × longer than wide (68 × 44 μm). Leg 6 not seen.

###### Etymology.

The specific name *cochleata* is derived from the Latin *cochl* (a spoon), alluding to the spoon-like mandible of the new species.

###### Remarks.

With the three-segmented rami of legs 1–4, the inner distal spine on the basis of leg 1, and the laterally positioned leg 5, the new species apparently belongs to the genus *Pontoclausia* which contains five known species (Ho and Kim 2003). Within the genus *Pontoclausiacochleata* sp. nov. may be clearly defined from other species by its three unique features: (1) the mandible is unarmed, with a scoop-like distal part, rather than armed with one or two armature elements as in congeners; (2) the maxilliped is unsegmented and tipped with one seta, rather than segmented and unarmed as in congeners; and (3) the third exopodal segment of leg 1 is armed with eight armature elements (formula III, I, 4), rather than six or seven elements as in congeners. It is remarkable that the form of the mandible of the new species is very unusual for the Clausiidae. Nevertheless, we have refrained from establishing a new genus, since other features of mouthparts and legs are as usual for the genera within Clausiidae.

##### ﻿ Pontoclausia pristina sp. nov.

Taxon classificationAnimaliaCyclopoidaClausiidae

0DB635BF-C436-51D7-9368-882771737A18

https://zoobank.org/EBD91A7F-FD84-4B52-BD6A-590BAB4C21B8

Figs 10 , 11 , 12

###### Material examined.

***Holotype*** ♂ (MABIK CR00250126) dissected and mounted on a slide, Site 1 (Sadong, Ulleung Island, 37°27'35.7"N, 130°52'34.6"E), 28 Jun. 2021, leg. J. G. Kim.

###### Description.

**Figure 10.**
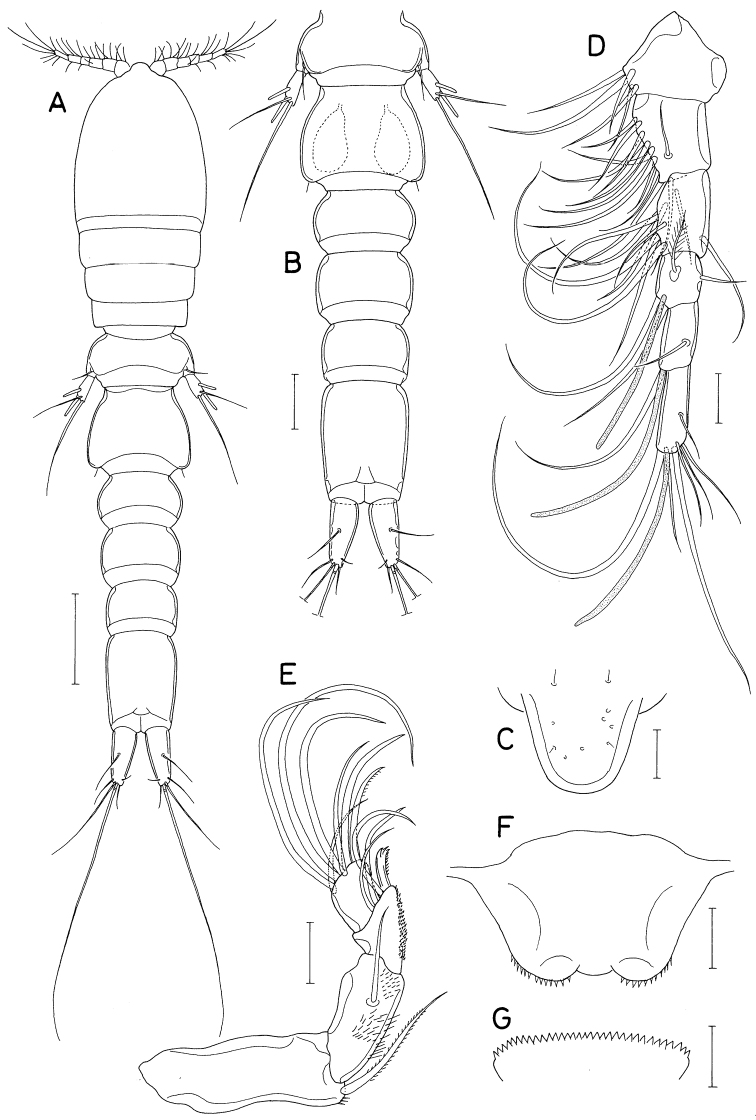
*Pontoclausiapristina* sp. nov., male **A** habitus, dorsal **B** urosome, dorsal **C** rostrum **D** antennule **E** antenna **F** labrum **G** labium. Scale bars: 0.2 mm (**A**); 0.1 mm (**B**); 0.02 mm (**C–G**).

**Male.** Body (Fig. 10A) harpacticiform, slender, cylindrical. Body length 1.60 mm. Prosome ~ twice longer than wide (593 × 295 μm), much shorter than urosome, consisting of cephalothorax and second to fourth pedigerous somites. Cephalothorax 363 μm long, longer than wide, with roundly produced rostral apex. Fourth pedigerous somite with angular posterolateral corners. Urosome (Fig. 10B) six-segmented. Fifth pedigerous somite 240 μm wide. Genital somite wider than long (194 × 230 μm), gradually broadened posteriorly. Four abdominal somites 115 × 188 μm, 127 × 179 μm, 109 × 160 μm, and 227 × 164 μm, respectively. Anal somite ~ twice longer than third abdominal somite. Caudal ramus (Fig. 10B) tapering, 2.46 × longer than wide (128 × 52 μm), armed with six thin, naked setae; distal longest seta (seta V) ~ 600 μm long, other setae short; seta II positioned dorsally at 48% region of ramus length.

Rostrum (Fig. 10C) well-sclerotized, gradually narrowed distally, with round apical margin. Antennule (Fig. 10D) 180 μm long, six-segmented; armature formula 5, 13, 9, 4+aesthetasc, 2+aesthetasc, and 7+aesthetasc; all setae naked except one on fourth segment; several of setae very long. Antenna (Fig. 10E) four-segmented; armature formula 1, 1, 3+claw, and 7; second segment (first endopodal segment) setulose on surfaces; third segment with densely arranged minute spinules on inner surface; claw of third segment distally trifurcate; terminal segment slightly longer than wide (17 × 15 μm); third outer seta on distal margin of terminal segment distinctly longer than other six setae.

**Figure 11.**
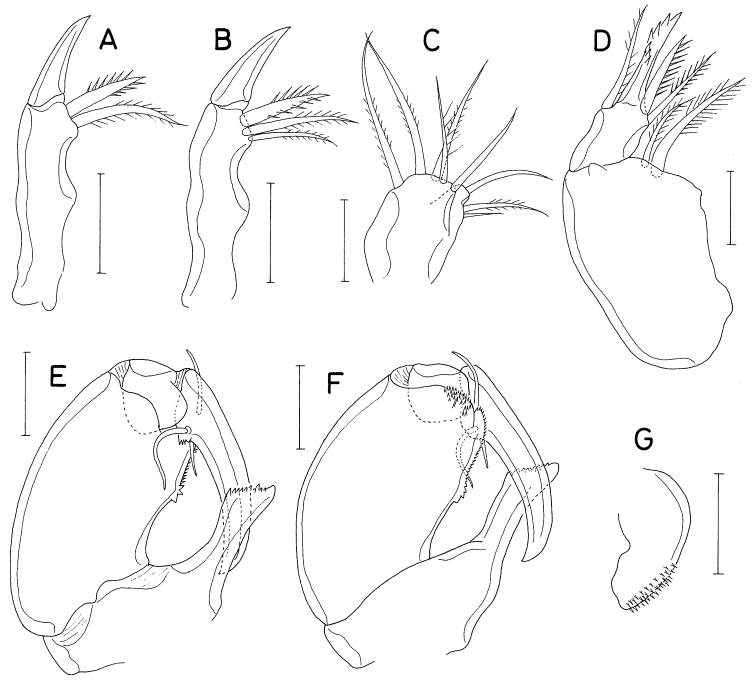
*Pontoclausiapristina* sp. nov., male **A, B** mandibles **C** maxillule **D** maxilla **E, F** maxillipeds **G** paragnath. Scale bars: 0.02 mm (**A–D, G**); 0.05 mm (**E, F**).

Mouthparts small, except large maxilliped. Labrum (Fig. 10F) with very shallow posterior incision, roundly convex posterolateral lobes fringed with spinules along their posterior margin. Labium (Fig. 10G) denticulate, saw-like. Mandible (Fig. 11A, B) distally armed with one strong, claw-like spine plus two or three spinulose or pinnate setae. Paragnath (Fig. 11G) as spinulose lobe. Maxillule (Fig. 11C) distally bilobed; with three setae on smaller inner lobe (proximalmost small, hardly visible) and five setae on larger outer lobe. Maxilla (Fig. 11D) two-segmented; proximal segment (syncoxa) with two unequal setae medio-distally; distal segment (basis) with three setae and one spiniform process bearing six denticles. Maxilliped (Fig. 11E, F) massive, consisting of three segments and terminal claw; first segment with large medio-distal process bearing truncate, spinulose distal margin; second segment unarmed but ornamented with spinules along distal half of inner margin and patch of spinules at inner distal region; short third segment unarmed; terminal claw strong, with three setae proximally (two on one side and one on opposite side).

**Figure 12.**
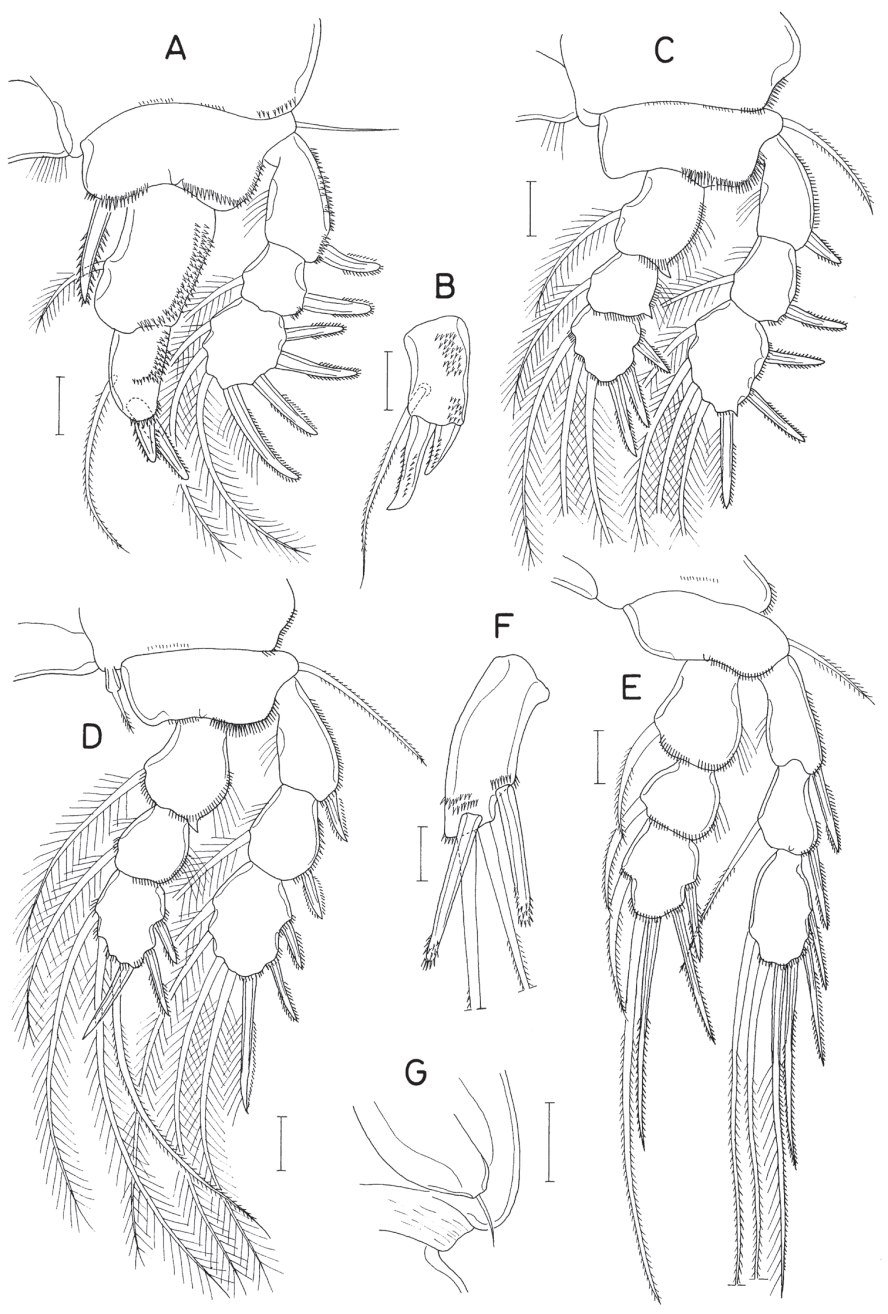
*Pontoclausiapristina* sp. nov., male **A** leg 1 **B** distal endopodal segment of leg 1 **C** leg 2 **D** leg 3 **E** leg 4 **F** exopod of leg 5 **G** left side of genital somite and leg 6, ventral. Scale bars: 0.02 mm (**A–F**); 0.05 mm (**G**).

Legs 1–4 (Fig. 12A–E) biramous. Inner coxal seta absent in legs 1, 2, and 4, but present in leg 3. Leg 1 with three-segmented exopod and two-segmented endopod; first endopodal segment inflated; inner distal spine on basis large, spinulose. Legs 2–3 with three-segmented rami. First and second endopodal segment of legs 2–4 bearing one inner seta. Inner coxal seta of leg 3 short, thickened in proximal third but thin, weakly pinnate in distal two-thirds. Distal setae on third endopodal segment of legs 2 and 3 very long. Leg 4 with finely spinulose setae; inner setae on endopod stiff; spines on both rami elongated, setiform, hardly distinguishable from setae. Armature formula for legs 1–4 as follows:

**Table:** 

	Coxa	Basis	Exopod	Endopod
Leg 1	0-0	1-I	I-0; I-1; III, I, 3	0-1; 0, II, 1
Leg 2	0-0	1-0	I-0; I-1; II, I, 4	0-1; 0-1; II, I, 3
Leg 3	0-1	1-0	I-0; I-1; II, I, 4	0-1; 0-1; II, I, 3
Leg 4	0-0	1-0	I-0; I-1; III, I, 2	0-1; 0-1; II, I, 2

Leg 5 (Fig. 10B) directed posterolaterally, clearly visible in dorsal view, consisting of one dorsolateral seta on fifth pedigerous somite and free exopod; exopodal segment 2.88 × longer than wide (72 × 25 μm), armed with two spines and two unequal setae; spines rod-shaped, spinulose in distal part, 60 and 52 μm long; setae spinulose, 245 and 136 μ long. Leg 6 (Fig. 12G) represented by one small, naked seta tipped on genital operculum.

**Female.** Unknown.

###### Etymology.

The specific name of the new species is derived from the Latin *pristin* (primitive), referring to the primitive condition of its antenna and mouthparts.

###### Remarks.

Although only a single male specimen is available for the description of *Pontoclausiapristina* sp. nov., it is distinctively characterized by its primitive antenna which is four-segmented with a full armature and by primitive, *Hemicyclops*-type mandible, maxillule and maxilla. The taxonomic position of the new species appears to be intermediate between the genera *Hemicyclops* and *Pontoclausia* of the Clausiidae. In the new species (1) the body is slender, harpacticiform (*Pontoclausia*-type feature); (2) the antennule is six-segmented (*Pontoclausia*-type); (3) the antenna is four-segmented, with 1, 1, 4, and 7 armature elements respectively on the first to fourth segments (*Hemicyclops*-type); (4) the mandible bears three or four distal armature elements (*Hemicyclops*-type); (5) the maxillule is distally bilobed with a total of eight setae (*Hemicyclops*-type); (6) the maxilla is two-segmented, with two distinct setae on the proximal segment and three armature elements plus one spiniform process on the distal segment (*Hemicyclops*-type); (7) the endopod of male leg 1 is two-segmented (*Pontoclausia*-type); (8) most of swimming legs lack the inner coxal seta (*Pontoclausia*-type); (9) the second endopodal segment of legs 2–4 bears only a single inner seta (*Pontoclausia*-type); and (10) the setation of the third exopodal and endopodal segments of most swimming legs is reduced (*Pontoclausia*-type). We consider that the two-segmented condition of the endopod of male leg 2 (above character state 7), which is a consistent, typical feature of *Pontoclausia*, is the most important taxonomic feature for determining the familial position of the new species; therefore, we place it within the Clausiidae. *Pontoclausiapristina* sp. nov. is distinguished from its congeners and other species in the family by the above *Hemicyclops*-type features.

### ﻿Family Kelleriidae Humes & Boxshall, 1996

#### Genus *Kelleria* Gurney, 1927

##### ﻿ Kelleria andamanensis

Taxon classificationAnimaliaCyclopoidaKelleriidae

Sewell, 1949

D7A79AC9-F083-52BF-9998-DAEF2D97B64C

###### Material examined.

One ♀, Site 15, 04 Jul. 2020.

###### Remarks.

Hong and Kim (2021) synonymized *K.grandisetiger* Kim, 2006 with *K.andamanensis* Sewell and redescribed it.

### ﻿Family Lichomolgidae Kossmann, 1877

#### Genus *Herrmannella* Canu, 1891

##### ﻿ Herrmannella dentata

Taxon classificationAnimaliaCyclopoidaLichomolgidae

Avdeev, 1987

8807C2BA-7FC0-5DE0-9F55-002B8EB51B5E

###### Material examined.

One ♀, Site 12, 16 Mar. 2013.

###### Remarks.

*Herrmannelladentata* was originally described as an associate of the bivalves *Myajaponica* Jay, 1857 and *Garikazusensis* (Yokoyama, 1922) in the Peter the Great Bay, Russia (Avdeev 1987). In Korea, this copepod species has been found only from *Myaarenaria* Linnaeus, 1758 (previously reported as *Myaarenariaoonogai* Makiyama, 1935) on the south coast.

##### ﻿ Herrmannella hoonsooi

Taxon classificationAnimaliaCyclopoidaLichomolgidae

Kim I.H., 1992

1003988B-9B39-5160-90E5-3D5A846A347D

###### Material examined.

One ♂, Site 11, 03 Jun. 2019; 1 ♀, 4 ♂♂, Site 12, 16 Mar. 2013; 1 ♂, Site 17, 13 May 2015.

###### Remarks.

This copepod species had been found only in the bivalve *Saxidomuspurpurata* (Sowerby, 1852).

##### ﻿ Herrmannella macomae

Taxon classificationAnimaliaCyclopoidaLichomolgidae

Kim I.H. & Sato, 2010

6F515870-D939-56D5-AA5C-34C6F663A439

###### Material examined.

One ♀, 3 ♂♂, Site 33, 11 Aug. 2020.

###### Remarks.

Kim and Sato (2010) described *Herrmannellamacomae* from the clam *Limecolacontabulata* (Deshayes, 1855) (recorded as *Macomacontabulata*) in Mutsu Bay, Japan. This copepod is new to the Yellow Sea, Korea and this is only the second record of the species.

#### Genus *Heteranthessius* Scott T., 1904

##### ﻿ Heteranthessius unisetatus sp. nov.

Taxon classificationAnimaliaCyclopoidaLichomolgidae

74243762-19C3-5936-90DE-FB617AC12F2A

https://zoobank.org/E7E5EAEE-6742-4312-8467-11EA6CF572F3

Figs 13 , 14

###### Material examined.

***Holotype*** ♂ (MABIK CR00250127) dissected and mounted on a slide, Site 6 (Jukbyeon Port, Uljin, 36°49'26.4"N, 129°26'52.2"E), 21 Sep. 2020, leg. J. Lee and J. G. Kim.

###### Description.

**Figure 13.**
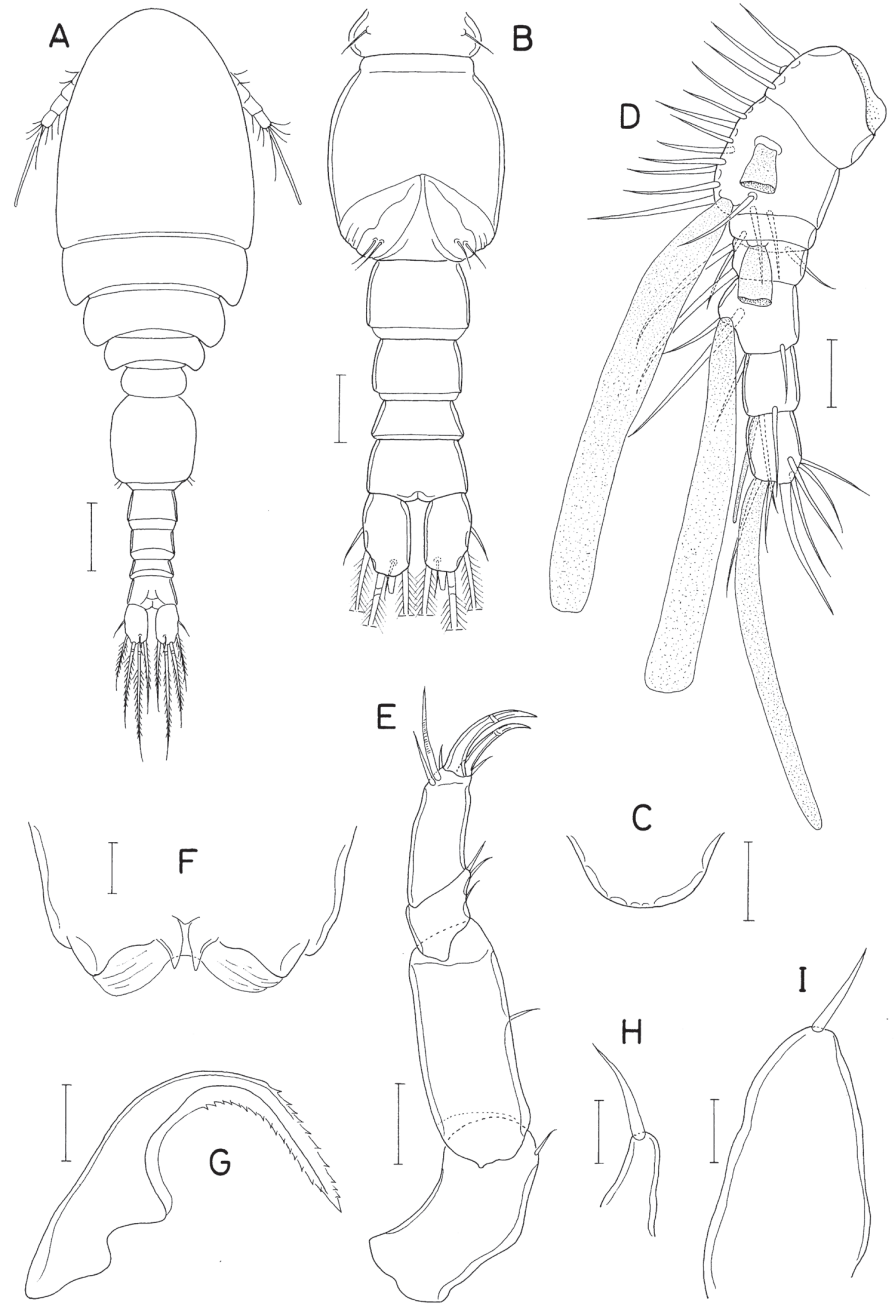
*Heteranthessiusunisetatus* sp. nov., male **A** habitus, dorsal **B** urosome, ventral **C** rostrum **D** antennule **E** antenna **F** labrum **G** mandible **H** maxillule **I** maxilla. Scale bars: 0.2 mm (**A**); 0.1 mm (**B, C**); 0.05 mm (**D, E**); 0.02 mm(**F–I**).

**Male.** Body (Fig. 13A) moderately narrow. Body length 1.92 mm. Prosome 1.08 mm long, comprising cephalothorax and second to fourth pedigerous somites. Cephalothorax 690 × 596 μm, distinctly longer than wide. All prosomal somites with rounded posterolateral corners. Urosome (Fig. 13B) six-segmented. Fifth pedigerous somite 200 μm wide. Genital somite subquadrate, longer than wide (309 × 265 μm), with rounded corners. Four abdominal somites 116 × 153 μm, 91 × 131 μm, 58 × 136 μm, and 91 × 149 μm, respectively. All abdominal somites smooth, without ornamentation. Caudal ramus broad, 1.64 × longer than wide (120 × 73 μm), with six setae; outer seta (seta II) short, naked, positioned at 45% region of ramus length; dorsal seta (seta VII) small and naked; other four setae pinnate.

Rostrum (Fig. 13C) broad, with round posterior margin. Antennule (Fig. 13D) 335 μm long, seven-segmented; armature formula 3, 12+2 aesthetascs, 2, 2+aesthetasc, 4+aesthetasc, 2+aesthetasc, and 7+aesthetasc; all setae naked; aesthetascs on second, fourth, and fifth segments large, broad, longer than antennular segments; aesthetasc on sixth segment small; aesthetasc on terminal segment as long as those of proximal segments but slender. Antenna (Fig. 13E) four-segmented, with armature formula 1, 1, 3, and 4+2 claws; terminal segment (third endopodal segment) gradually narrowed distally, 2.0 × longer than wide (76 × 38 μm); two terminal claws unequal, outer longer and thicker than inner, ~ 0.9 × as long as terminal segment.

**Figure 14.**
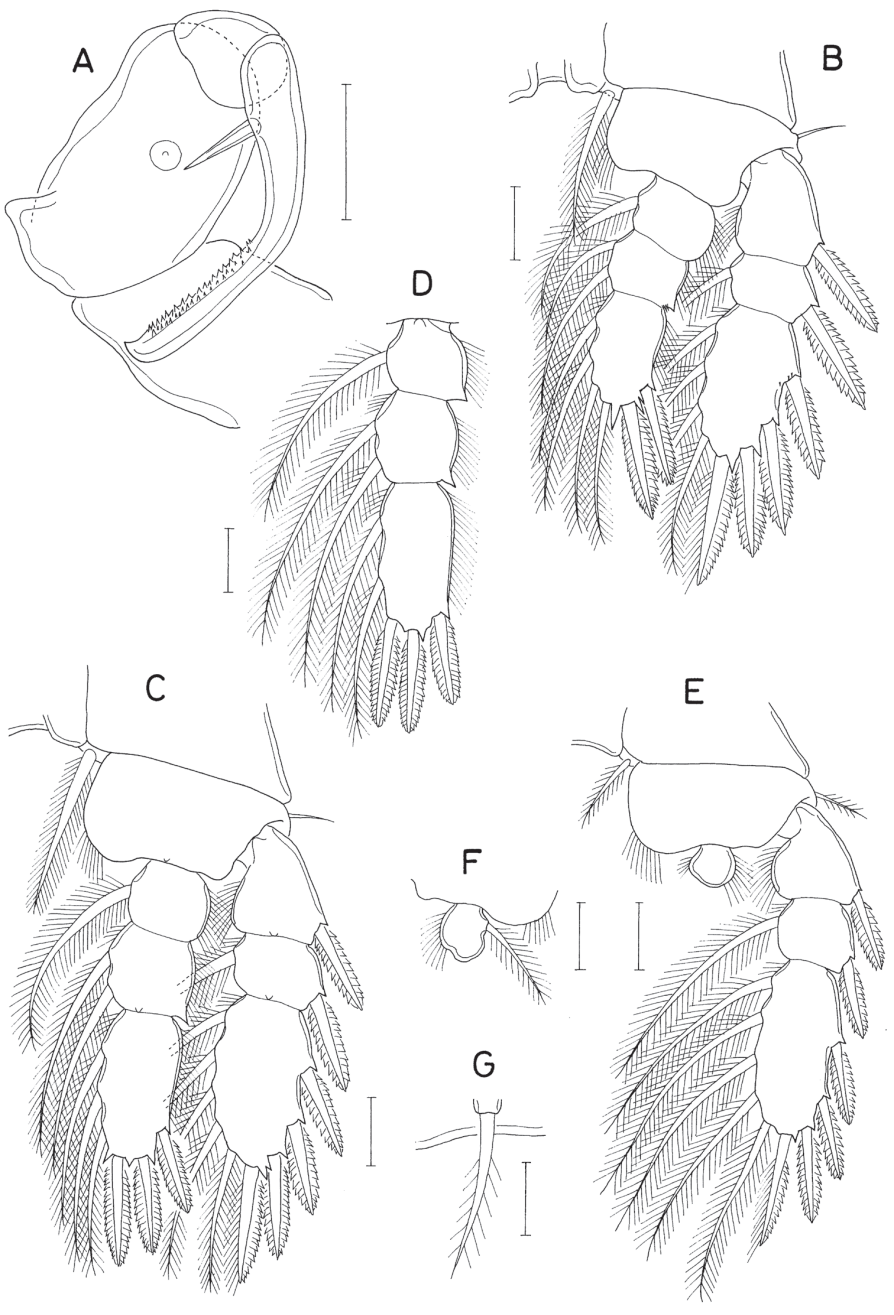
*Heteranthessiusunisetatus* sp. nov., male **A** maxilliped **B** leg 1 **C** leg 2 **D** endopod of left leg 3 **E** leg 4 **F** endopod of right leg 4 **G** leg 5. Scale bars: 0.05 mm (**A–F**); 0.02 mm (**G**).

Labrum (Fig. 13F) wider than long, with shallow posteromedian incision, fringed with broad membrane along posterior margin, pair of weak, tapering lobes at posteromedial region. Mandible (Fig. 13G) simple, with curved, elongate gnathobase bearing serrate margins. Maxillule (Fig. 13H) as small, digitiform lobe tipped with one naked seta. Maxilla (Fig. 13I) as large lobe tipped with one naked seta. Maxilliped (Fig. 14A) large, consisting of three segments and terminal claw; first segment as long as wide, unarmed; large second segment with one rudiment of seta and one large tubercle ventromedially; small third segment unarmed; terminal claw large, with one spine proximally and denticles on distal half of inner margin.

Legs 1–4 biramous; outer seta on basis small; spines on rami with densely serrate margins. Legs 1–3 (Fig. 14B–D) with three-segmented rami. Leg 4 (Fig. 14E) with three-segmented exopod and one-segmented endopod. Second endopodal segment of leg 1 characteristically with two inner setae. Endopod of leg 4 small, globular, with or without inner seta. Armature formula for legs 1–4 as follows:

**Table:** 

	Coxa	Basis	Exopod	Endopod
Leg 1	0-1	1-0	I-0; I-1; III, I, 4	0-1; 0-2; I, I, 4
Leg 2	0-1	1-0	I-0; I-1; III, I, 5	0-1; 0-2; I, II, 3
Leg 3	0-1	1-0	I-0; I-1; III, I, 5	0-1; 0-2; I, II, 2
Leg 4	0-1	1-0	I-0; I-1; III, I, 5	0, 0, 1 (or 0, 0, 0)

Leg 5 (Fig. 14G) represented by small papilla tipped with one pinnate seta 47 μm long. Leg 6 (Fig. 13B) represented by two small setae on genital operculum.

**Female.** Unknown.

###### Etymology.

The specific name of the new species is derived from Latin words, referring to the presence of a single seta on the maxillule and maxilla.

###### Remarks.

The genus *Heteranthessius* consists of four known species: *H.dubius* (T. Scott, 1903) from an unknown host in Scotland (T. Scott 1903), *H.scotti* Bocquet, Stock & Bernard, 1959 from calcareous algae at Roscoff, France (Bocquet et al. 1959), *H.furcatus* Stock, 1971 from a tunicate in the Mediterranean Sea (Stock 1971), and *H.hoi* López-González & Conradi, 1995 from an actiniarian at Gibraltar (López-González and Conradi 1995). *Heteranthessiusunisetatus* sp. nov. is easily distinguishable from the congeners by its unique morphological features: the maxillule bears only a single seta apically, against two setae in the four congeners, the maxilla is unsegmented, with a single seta apically, against two-segmented, with one spine or spiniform process and one seta on the distal segment in the congeners, and leg 5 is represented by a single seta, against two setae in the congeners. The most striking feature of the new species is the possession of two inner setae on the second endopodal segment of leg 1. Because the latter feature is very extraordinary and we have failed to find the same armature condition in other poecilostome copepods, it may be interpreted as an abnormality. However, it is remarkable that both left and right leg 1 display the same setation.

#### Genus *Modiolicola* Aurivillius, 1883

##### ﻿ Modiolicola bifidus

Taxon classificationAnimaliaCyclopoidaLichomolgidae

Tanaka, 1961

CF8682FC-A703-5282-A37E-7C65CF673226

###### Material examined.

Eight ♀♀, 2 ♂♂, Site 12, 16 Mar. 2013; 1 ♀, Site 23, 24 Apr. 2021.

###### Remarks.

*Modiolicolabifidus* has a very low host specificity and is distributed all around the Korean. Kim (2004) recorded 12 bivalve species as hosts, including *Ruditapesphilippinarum* (A. Adams & Reeve, 1850), the major host.

### ﻿Family Myicolidae Yamaguti, 1936

#### ﻿ Pusanomyicola gen. nov.

Taxon classificationAnimaliaCyclopoidaMyicolidae

019E2CE3-04C5-51F3-A795-4D0D711F9929

https://zoobank.org/D280A953-BE0A-458B-A438-650794FC0354

##### Diagnosis.

**Male.** Body narrow, cyclopiform, clearly segmented. Prosome consisting of cephalosome and four pedigerous somites. Urosome six-segmented. Caudal ramus with six setae, Antennule seven-segmented, heavily armed with setae and aesthetascs; first and second segments with multiple aesthetascs. Antenna three-segmented, consisting of coxobasis and two-segmented endopod, and terminated in single, strong claw. Labrum broader than long, with short posterolateral lobes. Mandible distally armed with three denticle-like elements, innermost one articulate at base. Maxillule as lobe tipped with two setae. Maxilla as lobe tipped with single seta. Maxilliped four-segmented; armature formula 0, 2, 0, and 1; terminal claw reduced, rudimentary. Legs 1–4 biramous, with three-segmented rami. Coxa of all swimming legs with small inner seta. Leg 1 lacking inner distal armature element on basis. Second endopodal segment of legs 2–4 armed with two inner setae. Third endopodal segment of legs 2 and 3 armed with three spines plus three setae (formula I, II, 3). Third exopodal segment of legs 3 and 4 armed with three spines plus five setae (formula II, I, 5). Leg 5 consisting of protopod and exopod; protopod well-defined from somite; exopod armed with three setae. Leg 6 represented by three setae on genital operculum.

##### Type species.

*Pusanomyicolasensitivus* gen. nov., sp. nov. (original designation).

##### Etymology.

The generic name is the combination of “Pusan”, the type locality of the type species, and *Myicola*, the type genus of the family. Gender masculine.

##### Remarks.

Boxshall and Halsey (2004) recognized eight genera in the family Myicolidae, including the highly transformed genus *Crucisoma* Kabata, 1981. While establishing the family Anthessiidae, Humes (1986) excluded *Conchocheres* Sars, 1918 from this family due to the lack of long elements on the mandible, the absence of the maxilliped in the female, and the presence of three setae only on the exopod of leg 5, but he did not determine the familial position of *Conchocheres*. Boxshall and Halsey (2004) tentatively placed *Conchocheres* in the Myicolidae on the basis of similarities in the armature of the antenna and in the form of the caudal rami, but they mentioned that the genus differed from all myicolids in the absence of the inner seta on the basis of leg 1, a characteristic found in the Anthessiidae.

**Figure 15.**
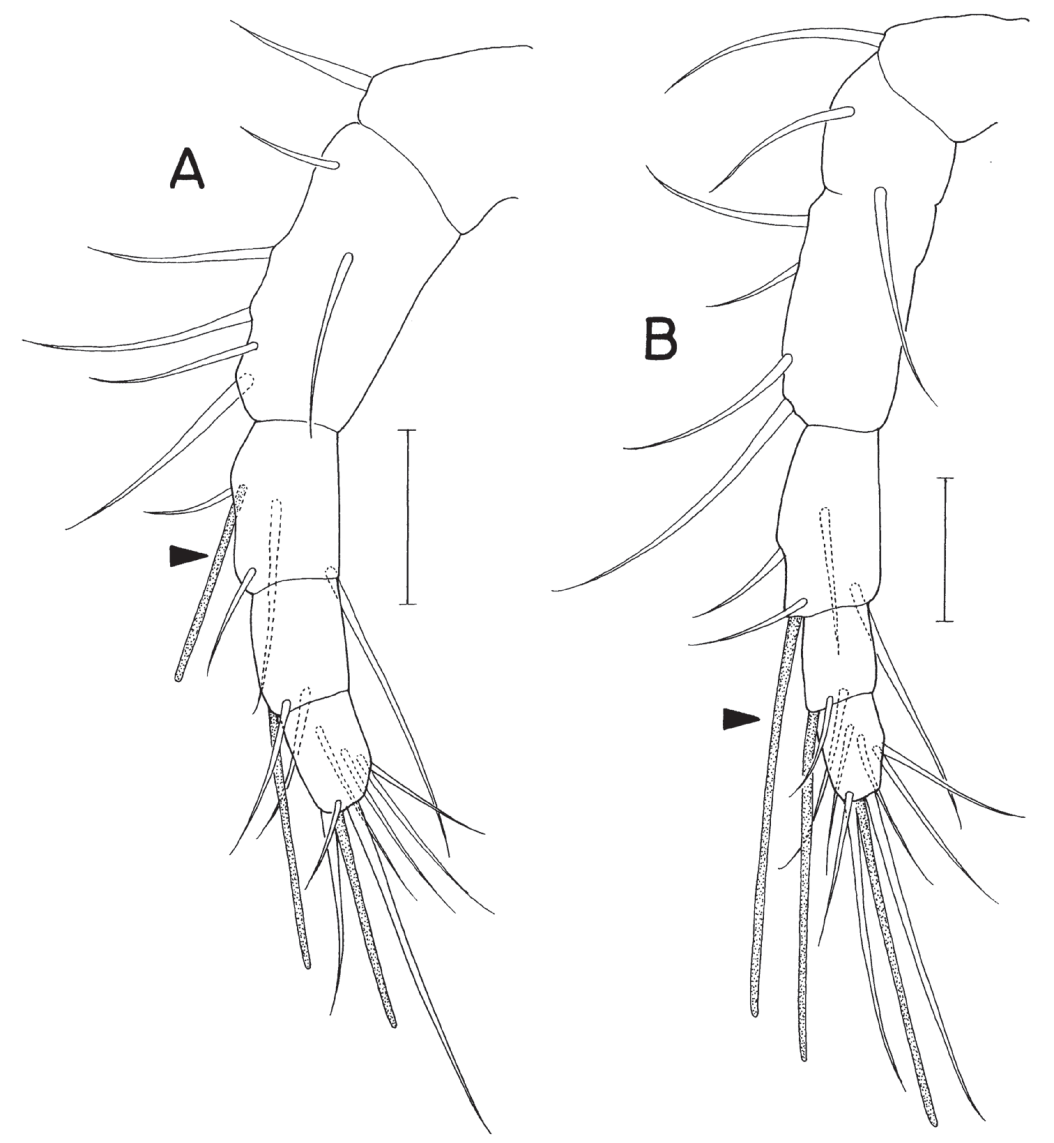
Antennule of copepodid II stage of *Critomolgusanthopleurus* Kim (**A**) and *Anthessiusdolabellae* Humes & Ho,1965 (**B**). Scale bars: 0.02 mm. **A** redrawn from Kim (2003).

It is notable that one typical feature of the Anthessiidae is in the antennule. In poecilostome cyclopods, the armature of three terminal segments of the antennule (4+aesthetasc, 2+aesthetasc, and 7+aesthetasc) is generally determined as early as the copepodid II stage, and this armature formula remains unchanged throughout subsequent developmental stages. However, the position of the aesthetasc on the antepenultimate segment (the segment of 4+aesthetasc) differs between the Anthessiidae and other poecilostome families, since the aesthetasc in the Anthessiidae is inserted at the distal corner, accompanied with anterodistal seta (Fig. 15B), whereas it is inserted near the proximal seta (Fig. 15A) in other poecilostome families, such as the Myicolidae, Clausidiidae, Ergasilidae, and lichomolgoid families. *Pusanomyicola* gen. nov. and *Conchocheres* share the armature pattern of the latter poecilostome families.

*Pusanomyicola* gen. nov. is more similar to *Conchocheres* than to any other known genera of the Myicolidae; their shared features are the unsegmented maxilla bearing a single distal element, the absence of the inner distal element on the basis of leg 1, and the possession of only three setae on the exopod of leg 5. Nevertheless, *Pusanomyicola* gen. nov. cannot be considered congeneric with *Conchocheres* due to their significant differences on the generic level, as follows: (1) the male urosome is five-segmented in *Conchocheresmalleolatus* Sars, 1918, the type and only species of *Conchocheres*, while it is six-segmented in *Pusanomyicolasensitivus* gen. nov., sp. nov.; (2) the male antennule of *C.malleolatus* bears five aesthetascs as illustrated by Sars (1918), but as many as 28 aesthetascs in *P.sensitivus* gen. nov., sp. nov.; (3) the male maxilliped of *C.malleolatus* bears a large terminal hook, while it is markedly reduced in *P.sensitivus* gen. nov., sp. nov.; (4) the third exopodal segment of leg 1 is armed with three spines plus four setae (II, I, 4) in *C.malleolatus*, but with four spines plus four setae (III, I, 4) in *P.sensitivus* gen. nov., sp. nov.; and (5) the third endopodal segment of leg 4 is armed with one spine plus three setae (I, 3) in *C.malleolatus*, but with three spines plus two setae (I, II, 2) in *P.sensitivus* gen. nov., sp. nov.

We place *Pusanomyicola* gen. nov. in the Myicolidae on the basis of its myicolid form of antenna bearing a single robust terminal claw and a truncate inner distal seta on the terminal segment, the presence of a group of spinules on the labrum, maxilla and genital operculum, and the myicolid form mandible. We confirm that *Conchocheres*, which shares important character states with *Pusanomyicola* gen. nov., is placed in the Myicolidae, as well.

#### ﻿ Pusanomyicola sensitivus

Taxon classificationAnimaliaCyclopoidaMyicolidae

gen. nov. sp. nov.

556C984A-88BE-54B3-AA4B-FAC730AE759C

https://zoobank.org/5C31B845-08AB-455D-9249-29B7617CCA87

Figs 16 , 17

##### Material examined.

***Holotype*** ♂ (MABIK CR00250128) dissected and mounted on a slide, Site 11 (Yeongdo, Pusan, 35°04'31.0"N, 129° 05'08.7"E), 07 Jul. 2020, leg. J. G. Kim.

##### Description.

**Figure 16.**
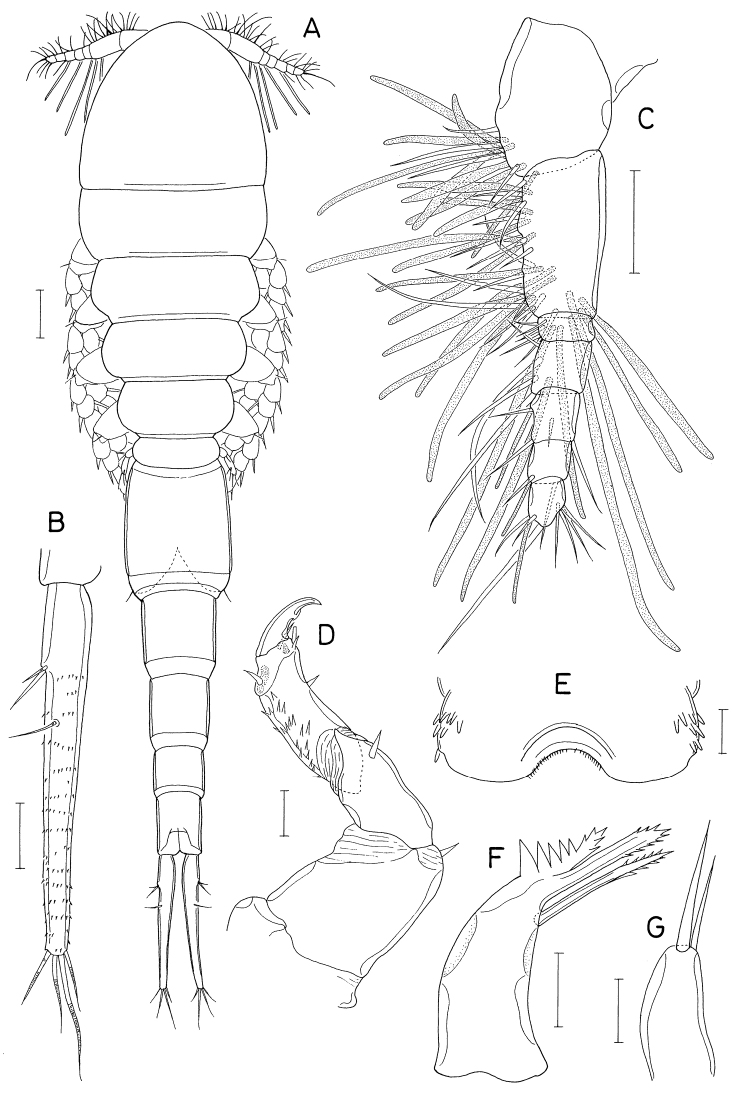
*Pusanomyicolasensitivus* gen. nov., sp. nov., male **A** habitus, dorsal **B** left caudal ramus, dorsal **C** antennule **D** antenna **E** labrum **F** mandible **G** maxillule. Scale bars: 0.1 mm (**A**); 0.05 mm (**B, C**); 0.02 mm (**D, E, G**); 0.01 mm(**F**).

**Figure 17.**
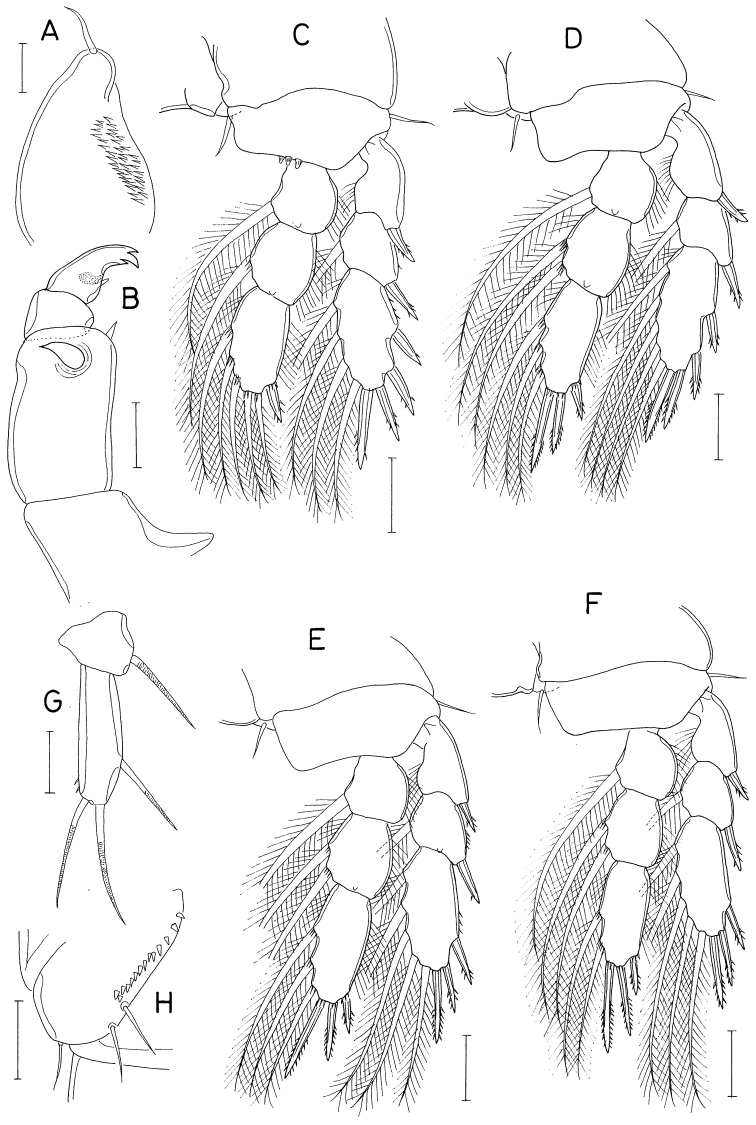
*Pusanomyicolasensitivus* gen. nov. sp. nov., male **A** maxilla **B** maxilliped **C** leg 1 **D** leg 2 **E** leg 3 **F** leg 4 **G** leg 5 **H** right genital operculum, ventral. Scale bars: 0.02 mm (**A, B, G**); 0.05 mm (**C–F, H**).

**Male.** Body (Fig. 16A) narrow, clearly segmented, gradually narrowed from anterior to posterior. Body length 2.06 mm. Maximum width 400 μm across cephalosome. Prosome 886 μm long, distinctly shorter than urosome, consisting of cephalosome and four pedigerous somites. All prosomal somites with rounded lateral margin. Urosome six-segmented. Fifth pedigerous somite short, narrower than genital somite. Genital somite nearly rectangular, 1.2 × longer than wide (273 × 227 μm); genital operculum (Fig. 17H) distinct, bearing three setae and row of scale-like spinules along inner distal margin. Four abdominal somites 177 × 159 μm, 150 × 127 μm, 100 × 109 μm, and 132 × 95 μm, respectively. Caudal ramus (Fig. 16B) slender, 7.9 × longer than wide (284 × 36 μm), armed with seven setae (seta I to VII), ornamented with many transverse rows of minute spinules; setae I and II positioned at same place at 23% length of ramus on outer margin; setae III–VI positioned on distal margin; seta VII positioned on dorsal surface at 38% length of ramus; all caudal setae naked, short, longest one (seta V) one-third as long as ramus.

Rostrum not developed. Antennule (Fig. 16C) 245 μm long, seven-segmented, densely armed with setae and aesthetascs; armature formula 4+7 aesthetascs, 15+15 aesthetascs, 4+2 aesthetascs, 4+aesthetasc, 2+aesthetasc, and 7+aesthetasc; all setae naked, mostly short; aesthetascs shorter than antennule. Antenna (Fig. 16D) three-segmented, consisting of coxobasis and two-segmented endopod; coxobasis as long as wide, with one short seta at inner distal corner; first endopodal segment ~ 1.5 × longer than wide, with one small seta at inner subdistal region; second endopodal segment twice longer than wide, terminated in strong claw, armed with five small setae (one on inner margin, one on subdistal outer margin, and three at inner distal corner, one of latter truncate), and ornamented with scattered scale-like spinules on outer surface; terminal claw half as long as second endopodal segment.

Labrum (Fig. 16E) much wider than long, with patch of several blunt spinules on each lateral surface; posteromedian incision shallow, semicircular, with finely spinulose margin. Mandible (Fig. 16F) narrowed distally, armed with three armature elements distally: short outer element not articulated at base, with five large teeth along outer margin and five denticles distally; longest middle element (stiff lash) straight, not articulated at base, with several denticles at distal part; slender inner element (spiniform seta) articulated at base, as long as middle element, denticulate distally. Maxillule (Fig. 16G) as digitiform lobe tipped with two unequal, naked setae. Maxilla (Fig. 17A) as tapering, unsegmented lobe bearing patch of spinules on posteroventral surface, tipped with one naked seta. Maxilliped (Fig. 17B) four-segmented; first segment (syncoxa) broader than long, unarmed; second segment (basis) rectangular, armed with one broad, leaf-like seta subdistally and one small seta distally; short third segment (first endopodal segment) unarmed; terminal segment (second endopodal segment) tapering, curved, trifurcate at tip, with one small seta on inner margin; claw or hook absent (or reduced to small middle process of distal tip).

Legs 1–4 (Fig. 17C–F) biramous, with three-segmented rami; inner coxal seta small, naked; outer seta on basis also small, naked; both rami of each leg almost equal in length; spines on exopods and endopods spinulose along both margins. Leg 1 lacking inner distal element of basis, but with three blunt dentiform spinules near base of endopod. Legs 3 and 4 with same armature on third exopodal segment. Armature formula for legs 1–4 as follows:

**Table:** 

	Coxa	Basis	Exopod	Endopod
Leg 1	0-1	1-0	I-0; I-1; III, I, 4	0-1; 0-1; I, 2, 3
Leg 2	0-1	1-0	I-0; I-1; III, I, 5	0-1; 0-2; I, II, 3
Leg 3	0-1	1–0	I-0; I-1; II, I, 5	0-1; 0-2; I, II, 3
Leg 4	0-1	1-0	I-0; I-1; II, I, 5	0-1; 0-2; I, II, 2

Leg 5 (Fig. 17G) small, consisting of protopod and exopod; protopod articulated from somite, as long as wide, with one seta dorsodistally; exopod 3.0 × longer than wide (45 × 15 μm), armed with three naked setae (two on distal margin and one at 75% region of dorsal margin), ornamented with few spinules ventrodistally; setae on protopod and exopod shorter than exopodal segment. Leg 6 (Fig. 17H) represented by three naked setae on inner distal margin of genital operculum.

**Female.** Unknown.

##### Etymology.

The specific name *sensitivus* refers to the presence of the multiple aesthetascs on the male antennule.

### ﻿Family Polyankyliidae Ho & Kim I.H., 1997

#### Genus *Polyankylis* Ho & Kim I.H., 1997

##### ﻿ Polyankylis ovilaxa

Taxon classificationAnimaliaCyclopoidaPolyankyliidae

Kim, 2014

28A2073F-3352-50FF-AB01-B5882111C159

###### Material examined.

One ♀, Site 27, 09 Jul. 2016.

###### Remarks.

Kim (2014) described this copepod as an associate of the terebellid polychaete *Thelepusjaponicus* Marenzeller, 1884 from the south coast of Korea.

##### ﻿ Polyankylis bogilensis sp. nov.

Taxon classificationAnimaliaCyclopoidaPolyankyliidae

240BC9A3-9AD3-5D65-8CB3-BB786A29E130

https://zoobank.org/DCF49374-198A-4824-910A-45D3C72A4C81

Figs 18 , 19

###### Material examined.

***Holotype*** ♀ (MABIK CR00250129) dissected and mounted on a slide, Site 22 (Yesong, Bogil Island, south coast, 34°08'11"N, 126°33'49"E), 31 May 2021, leg. J. Lee.

###### Description.

**Figure 18.**
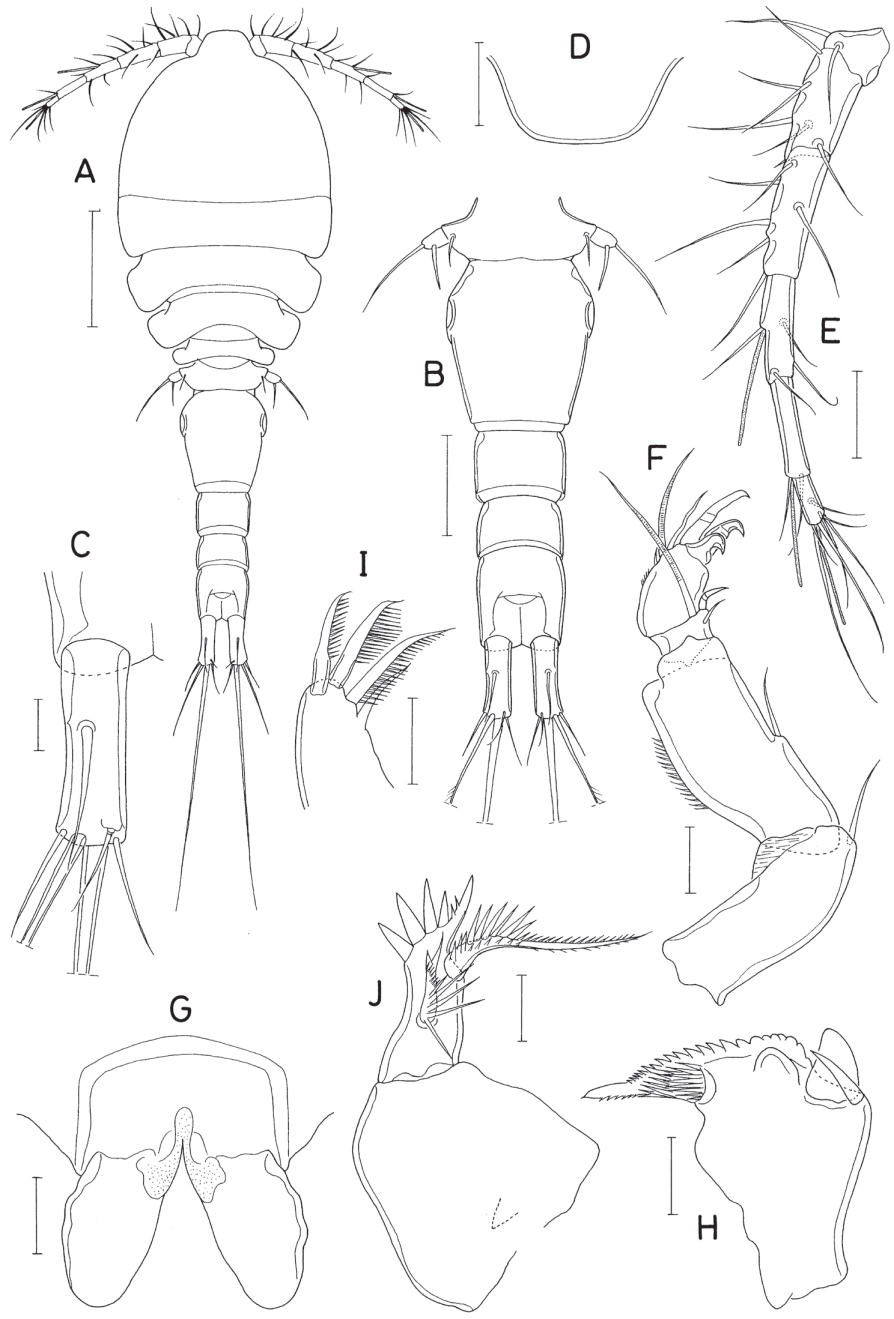
*Polyankylisbogilensis* sp. nov., female **A** habitus, dorsal **B** urosome, dorsal **C** left caudal ramus, dorsal **D** rostrum **E** antennule **F** antenna **G** labrum **H** mandible **I** maxillule **J** maxilla. Scale bars: 0.2 mm (**A**); 0.1 mm (**B**); 0.02 mm (**C, F–J**); 0.05 mm (**D, E**).

**Female.** Body (Fig. 18A) dorsoventrally flattened. Body length 1.10 mm. Prosome 1.54 × longer than wide (570 × 370 μm). Cephalothorax with faint dorsal suture line between cephalosome and first pedigerous somite, with rounded posterolateral corners. Second to fourth pedigerous somites with rounded anterolateral and posterolateral corners. Urosome (Fig. 18B) six-segmented. Fifth pedigerous somite 155 μm wide, slightly wider than genital double-somite. Genital double-somite 1.15 × longer than wide (170 × 148 μm), widest at proximal third of double-somite; posterior two-thirds gradually narrowing posteriorly; genital apertures positioned dorsolaterally at widest region of double-somite. Three free abdominal somites 70 × 93 μm, 56 × 86 μm, and 90 × 90 μm, respectively. All urosomal somites smooth, unornamented. Caudal rami straight backwards, rectangular, isolated from each other; each ramus (Fig. 18C) 2.96 × longer than wide (80 × 27 μm), armed with six setae (seta II-VII); seta II positioned dorsally at 45% region of ramus length; seta V much longer than other caudal setae; seta III feebly pinnate, other five setae naked.

Rostrum (Fig. 18A, D) as broad, spatulate anterior prominence of cephalothorax. Antennule (Fig. 18E) 295 μm long, six-segmented, gradually narrowed distally; armature formula 2, 7, 6 (or 2. 6, 7), 4+aesthetasc, 2+aesthetasc, and 7+aesthetasc; all setae naked. Antenna (Fig. 18F) four-segmented; first segment (coxobasis) with one seta at inner distal corner; second segment (first endopodal segment) longest, armed with one seta on inner margin and ornamented with setules on outer margin; short third segment with one small claw and two very unequal setae; terminal segment 1.28 × longer than wide (23 × 18 μm), distally armed with three claws of different lengths and three unequal setae, including minute outermost seta, and ornamented with minute spinules on subdistal outer margin.

**Figure 19.**
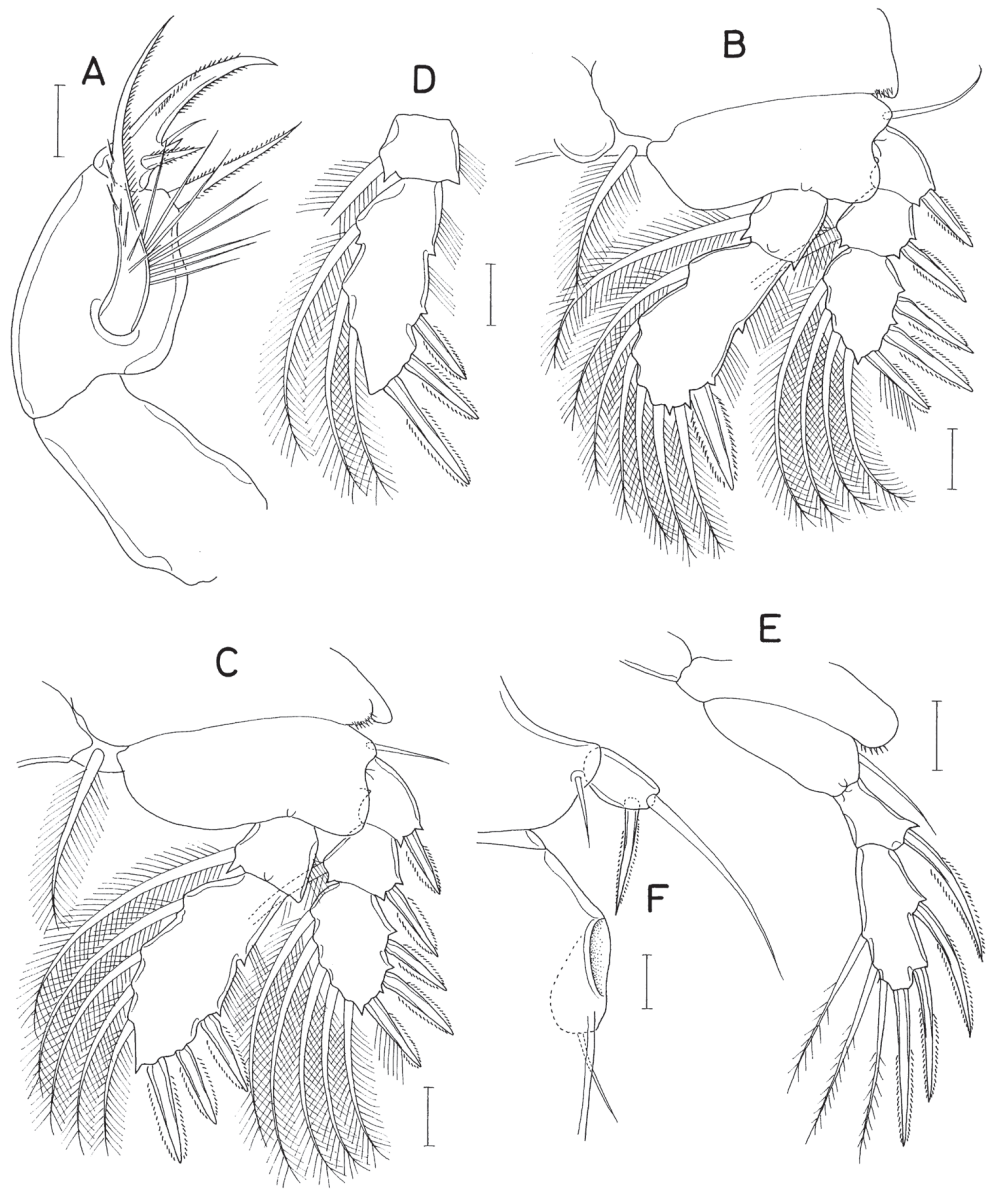
*Polyankylisbogilensis* sp. nov., female **A** maxilliped **B** leg 1 **C** leg 2 **D** endopod of leg 3 **E** leg 4 **F** leg 5 and genital aperture, dorsal. Scale bars: 0.02 mm.

Labrum (Fig. 18G) bilobed, with deep median incision and proximal sclerotization band; each lobe distinctly longer than wide (~ 41 × 25 μm), divided from proximal part by weak suture line, with uneven outer margin. Mandible (Fig. 18H) with two very unequal outer scales (spiniform proximal one and large, plate-like distal one) followed by stout tubercle; inner margin short, with circular row of spinules at junction between distal lash and inner margin; distal lash short, denticulate along outer margin, with fine denticle along inner margin; terminal part of lash not flexible. Maxillule (Fig. 18I) lobate, armed with four setae; larger distal three setae pectinate along their inner margin; smaller inner margin seta naked, not articulated at base. Maxilla (Fig. 18J) two-segmented; proximal segment (syncoxa) broad, with one claw-like cusp on proximal part of posterior surface; distal segment (basis) armed with two spiniform setae (setae I and II), terminating in short, spiniform distal lash bearing four spines followed by one or two denticles along outer margin; seta I (inner seta) large, spinulose, proximal six spinules markedly larger than other spinules on seta; seta II (anterior seta) distally unequally bifurcate, with row of spinules, proximal four or five of these spinules much larger than distal spinules. Maxilliped (Fig. 19A) three-segmented; first segment (syncoxa) unarmed; second segment (basis) broadened, armed with two large setae distantly isolated from each other: proximal seta spiniform, curved, extending to distal tip of maxilliped, ornamented with three kinds of spinules, eight extremely long spinules on proximal part of inner margin followed distally by minute spinules and row of several small spinules along outer margin; distal seta straight, less than half as long as proximal seta, feebly spinulose along both margins; terminal segment (endopod) distally forming spinulose claw, proximally with one spine, one dentiform process and one small seta.

Leg 1–3 (Fig. 19B–D) biramous, each with three-segmented exopod and two-segmented endopod; coxa with minute spinules at outer distal corner and large, pinnate inner seta; outer seta on basis naked. Leg 3 dissimilar to leg 2 in having three inner setae (instead of four) on distal endopodal segment. Leg 4 (Fig. 19E) uniramous, with distinctly two-segmented exopod; endopod absent; coxa lacking inner seta; spines on exopod elongate. Armature formula for legs 1–4 as follows:

**Table:** 

	Coxa	Basis	Exopod	Endopod
Leg 1	0-1	1-0	I-0; I-1; III, 4	0-1; I, 1, 5
Leg 2	0-1	1-0	I-0; I-1; III, 5	0-1; III, 4
Leg 3	0-1	1-0	I-0; I-1; III, 5	0-1; III, 3
Leg 4	0-0	1-0	I-0; II, I, 3	(lacking)

Leg 5 (Fig. 19F) consisting of one dorsolateral seta on fifth pedigerous somite and free exopod; exopodal segment 1.72 × longer than wide (31 × 18 μm) armed with one spine (45 μm long) one naked seta (91 μm long). Leg 6 unarmed (Fig. 19F).

**Male.** Unknown.

###### Etymology.

The name of the new species is taken from the type locality, Bogil Island.

###### Remarks.

The genus *Polyankylis* currently consists of three known species: *P.orientalis* Ho & Kim, 1997, *P.australis* Karanovic, 2008, and *P.ovilaxa*. *Polyankylisaustralis* is known from Australia (Karanovic 2008) and the other two from Korea (Ho and Kim 1997; Kim 2014). These three species are distinguished from *P.bogilensis* sp. nov. by different features, as follows: *P.orientalis* has a claw-like distal process on the coxobasis of the antenna (cf. this process absent in *P.bogilensis* sp. nov.) and a single-segmented exopod of leg 4 (cf. two-segmented in *P.bogilensis* sp. nov.); *P.australis* has an aesthetasc on the second endopodal segment of the antenna (cf. this aesthetasc absent in *P.bogilensis* sp. nov.), the terminal antennal segment is 3.5 × longer than wide (cf. 1.28 × longer than wide in *P.bogilensis* sp. nov.), and the maxillary syncoxa lacks a claw-like process (cf. this process present in *P.bogilensis* sp. nov.); and *P.ovilaxa* has caudal rami which are 4.40 × longer than wide (cf. 2.96 × in *P.bogilensis* sp. nov.), the terminal antennal segment is 2.5 × longer than wide, and the maxillary syncoxa lacks the claw-like process.

### ﻿Family Pseudanthessiidae Humes & Stock, 1972

#### Genus *Pseudanthessius* Claus, 1889

##### ﻿ Pseudanthessius linguifer sp. nov.

Taxon classificationAnimaliaCyclopoidaPseudanthessiidae

E2AA7EA4-BF29-5546-A5ED-C4D03106245B

https://zoobank.org/99BB54CC-6289-4E0D-9B8C-8D4AE3EF5805

Figs 20 , 21

###### Material examined.

***Holotype*** ♀ (MABIK CR00250120) and intact ***paratypes*** 3 ♀♀ (MABIK CR00250121) preserved in 90% alcohol, and paratype ♀ dissected and mounted on a slide, Site 22 (Yesong, Bogil Island, south coast, 34°08'11"N, 126°33'49"E), 31 May 2021, leg. J. Lee; ♀ dissected and mounted on a slide, Site 23 (Haenam, south coast, 34°17'57"N, 126°31'50"E), 24 Apr. 2021, leg. J. Lee and C. Y. Chang. Dissected specimens are retained in the collection of I.-H. Kim.

###### Description.

**Figure 20.**
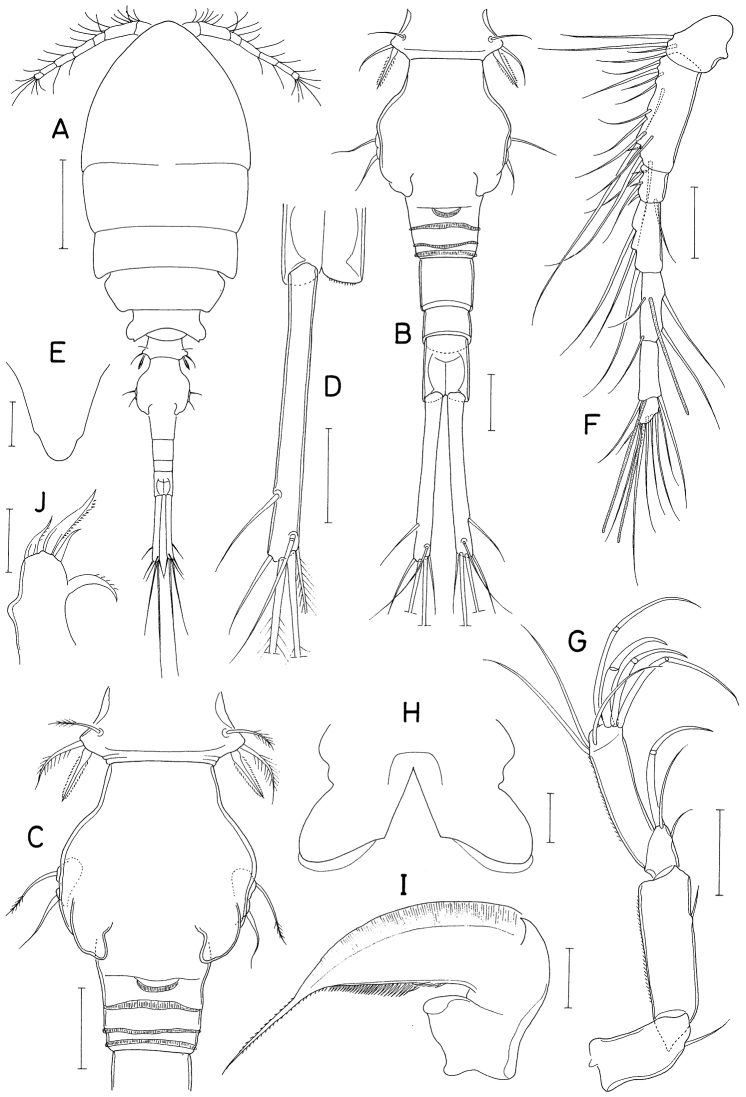
*Pseudanthessiuslinguifer* sp. nov., female **A** habitus, dorsal **B** urosome, dorsal **C** proximal somites of urosome, dorsal **D** left caudal ramus, dorsal **E** rostrum **F** antennule **G** antenna **H** labrum **I** mandible **J** maxillule. Scale bars: 0.2 mm (**A**); 0.05 mm (**B– D, F, G**); 0.02 mm (**E, H–J**).

**Figure 21.**
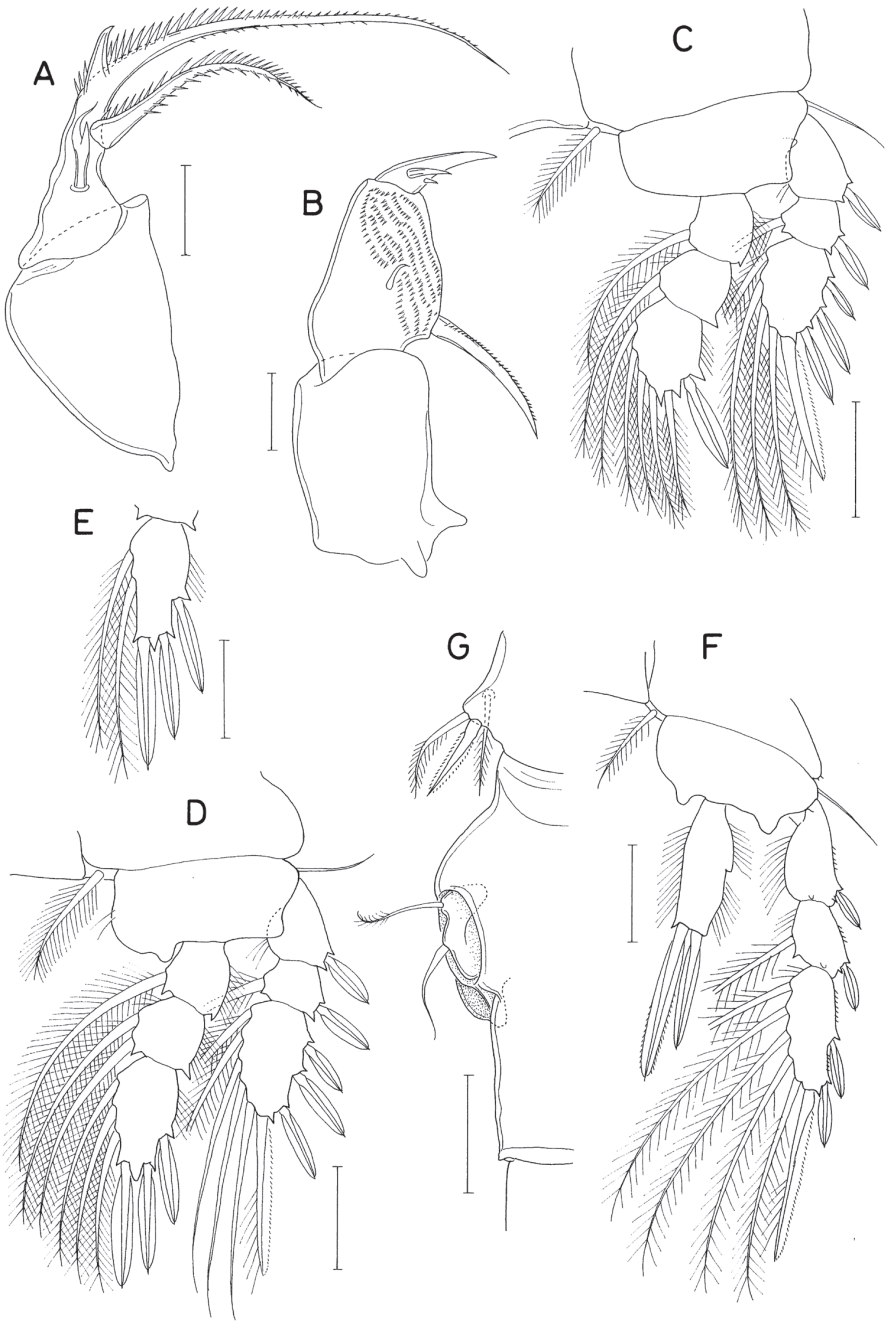
*Pseudanthessiuslinguifer* sp. nov., female **A** maxilla **B** maxilliped **C** leg 1 **D** leg 2 **E** third endopodal segment of leg 3 **F** leg 4 **G** leg 5 and genital aperture, dorsal. Scale bars: 0.02 mm (**A, B**); 0.05 mm (**C–G**).

**Female.** Body (Fig. 20A) narrow. Body length of dissected and figured paratype 1.23 mm (length range 1.17–1.32 mm, holotype 1.19 mm). Maximum width 385 μm across cephalothorax. Prosome 727 μm long. Cephalothorax 463 μm long, distinctly longer than wide, with weak dorsal suture line delimiting cephalosome and first pedigerous somite. Fourth pedigerous somite with point near posterolateral corners; other prosomal somites with rounded corners. Urosome (Fig. 20B) shorter than prosome, five-segmented. Fifth pedigerous somite 102 μm wide. Genital double-somite ~ 1.5 × longer than wide (182 × 123 μm), consisting of narrow anterior 17%, inflated middle 49%, and narrow posterior 34%; dorsally covered by brownish sticky material; genital apertures characteristically positioned ventrolaterally (Fig. 21G) at 45% region of double-somite length; broader middle region bearing linguiform process dorsolaterally, posterior to each genital aperture (Fig. 20C); narrow posterior region with four horizontal membranous flanges (Fig. 20C) on dorsal surface, anterior one short, curved. Three free abdominal somites 45 × 49 μm, 25 × 44 μm, and 56 × 42 μm, respectively. Anal somite with minute spinules along posteroventral margin. Caudal ramus (Fig. 20D) elongate, 10 × longer than wide (155 × 15.5 μm), 2.77 × longer than anal somite, armed with six setae (seta II–VII); seta II (outer lateral seta) positioned at 78% length of ramus; setae IV–VI pinnate, other three setae naked.

Rostrum (Fig. 20E) tapering, as long as wide, abruptly narrowed subdistally, with round apex. Antennule (Fig. 20F) 295 μm long, seven-segmented; armature formula 4, 13, 6, 3, 4+aesthetasc, 2+aesthetasc, and 7+aesthetasc; all setae thin, naked; aesthetascs also thin, setiform. Antenna (Fig. 20G) four-segmented; first segment (coxobasis) with one seta inner distally; second segment (first endopodal segment) with one seta on inner margin and fine spinules along outer margin; third segment short, armed with one slender claw and two setae; terminal segment 3.28 × long than wide (77 × 23 μm), armed with four slender claws (inner and outer claws longer than middle two) plus three setae, and ornamented with fine spinules along outer margin.

Labrum (Fig. 20H) with long, divergent posterolateral lobes, with deep median incision; each lobe with angle on inner margin; posterior margin of lobes fringed with membrane. Mandible (Fig. 20I) with one large, tooth-like outer scale; gnathobase tapering, with row of minute spinules along inner margin, terminating in long, thin lash. Maxillule (Fig. 20J) with four unequal setae (three apical and one on inner margin) and one blunt tubercle on outer margin; middle of three distal setae larger than other two. Maxilla (Fig. 21A) two-segmented; proximal segment (syncoxa) unarmed; distal segment (basis) with extremely long distal lash and armed with two setae (setae I & II); distal lash longer than remaining part maxilla, bearing one large claw-like process proximally, spinulose along convex outer margin; seta I large, slightly longer than half length of distal lash, spinulose along both margins; seta II unequally bifurcate at tip, with setiform outer furca and spinule-like inner furca; seta III absent. Maxilliped (Fig. 21B) three-segmented; first segment (syncoxa) longest but unarmed; second segment (basis) armed with two very unequal setae (proximal seta large, spiniform, longer than width of segment, more than 4 × as long as small distal seta), and ornamented with several longitudinal rows of fine spinules on inner surface; small third segment (endopod) tapering, claw-like, proximally with one spine and one small seta.

Legs 1–4 (Fig. 21C–F) biramous. Legs 1–3 with three-segmented rami. Leg 4 with three-segmented exopod and one-segmented endopod. Inner coxal seta well-developed, pinnate in legs 1–4. Outer seta on basis thin, naked. Distal process between two distal spines on third endopodal segment of leg 2 blunt, slightly swollen. Three inner distal setae on third exopodal segment of legs 2 and 3 naked. Endopodal segment of leg 4 setulose on inner and outer margins, 2.6 × longer than wide (68 × 26 μm), bearing angle on outer margin; two distal spines 82 (inner) and 61 μm long (outer). Armature formula for legs 1–4 as follows:

**Table:** 

	Coxa	Basis	Exopod	Endopod
Leg 1	0-1	1-0	I-0; I-1; III, I, 4	0-1; 0-1; I, 1, 4
Leg 2	0-1	1-0	I-0; I-1; III, I, 5	0-1; 0-2; I, II, 3
Leg 3	0-1	1-0	I-0; I-1; III, I, 5	0-1; 0-2; I, II, 2
Leg 4	0-1	1-0	I-0; I-1; II, I, 5	0, II, 0

Leg 5 (Fig. 21G) represented by one spine and two setae on lateral surface of fifth pedigerous somite. Leg 6 (Fig. 21G) represented on two setae on genital operculum; anterior seta thin, weakly pinnate; posterior seta naked, proximally broadened.

**Male.** Unknown.

###### Etymology.

The specific name of the new species *linguifer* is derived from Latins *lingu* (the tongue) and *fer* (bear), referring to the presence of the tongue-like dorsolateral processes on the genital double-somite.

###### Remarks.

The most conspicuous feature of *Pseudanthessiuslinguifer* sp. nov. is its elongate caudal rami, which are 10 × longer than wide. Such long caudal rami are exhibited by four congeners: *P.concinnus* Thompson & Scott, 1903, *P.dubius* Sars, 1918, *P.thorelli* (Brady & Robertson, 1875), and *P.stenosus* Kim & Hong, 2014. All of the other species in the genus have shorter caudal rami, at most 8.5 × longer than wide, as in *P.deficiens* Stock, Humes & Gooding, 1964 (Stock et al. 1964). *Pseudanthessiuslinguifer* sp. nov. differs from *P.concinnus* in having a large outer scale on the mandible (cf. the scale absent in *P.concinnus*) and two distal spines on the endopod of leg 4 (cf. one spine plus one seta in *P.concinnus*); from *P.dubius* in having the five-segmented urosome in the female (cf. four-segmented female urosome in *P.dubius*) and four distal claws on the antenna (cf. a single large claw in *P.dubius*); and from *P.thorelli* in having one spine plus one seta on the exopod of female leg 5 (cf. two setae in *P.thorelli*). *Pseudanthessiuslinguifer* sp. nov. resembles *P.stenosus* which is known from Thailand (Kim and Hong 2014) in many morphological aspects, in particular, the possession of the spinules-covered second segment (basis) of the female maxilliped and the bifurcate anterior seta (seta II) on the basis of the maxilla. However, the new species is distinguishable from *P.stenosus* and other congeners by its other outstanding features, such as the presence of the tongue-like dorsolateral processes on the genital double-somite, the extremely long distal lash of the maxilla, and the ventrolateral position of the genital apertures.

### ﻿Family Rhynchomolgidae Humes & Stock, 1972

#### Genus *Critomolgus* Humes & Stock, 1983

##### ﻿ Critomolgus anthopleurus

Taxon classificationAnimaliaCyclopoidaRhynchomolgidae

Kim I.H., 1996

5BD612D9-0523-58B0-95A8-44D93F960C78

###### Material examined.

One ♀, Site 4, 19 Jul. 2016.

###### Remarks.

The host of this copepod is the actiniarian *Anthopleuraanjunae* Den Hartog & Vennam, 1993. The previously recorded host name *Anthopleuramidori* Uchida & Muramatsu, 1958 is a junior synonym of *A.anjunae* (WoRMS Editorial Board 2021). It is remarkable that *C.anthopleurus* is an internal associate, living within the gastrovascular cavity of the actiniarian, but can be attracted to light.

### ﻿Family Sabelliphilidae Gurney, 1927

#### Genus *Eupolymniphilus* Humes & Boxshall, 1996

##### ﻿ Eupolymniphilus orientalis

Taxon classificationAnimaliaCyclopoidaSabelliphilidae

Kim, 2006

E5FF658A-E8A9-5A75-8F58-8BBB743EB513

###### Material examined.

Five ♀♀, 2 ♂♂, Site 22, 26 Apr. 2021; 5 ♂♂, Site 27, 09 Apr. 2016.

###### Remarks.

The host of this copepod is still unknown but is probably a polychaete.

##### ﻿ Eupolymniphilus foliatus sp. nov.

Taxon classificationAnimaliaCyclopoidaSabelliphilidae

C12C171F-5B70-5762-9692-F7C8B2BB998B

https://zoobank.org/BBF87DB7-F7BA-4642-8951-456E4885F148

Figs 22 , 23 , 24

###### Material examined.

***Holotype*** ♀ (MABIK CR00250130) and ***paratype*** ♂ dissected and mounted on a slide, and intact ***paratypes*** 2 ♂♂ (MABIK CR00250122) preserved in 90% alcohol, Site 2 (Namyang, Ulleung Island, Sea of Japan, 37°28'01.3"N, 130°50'01.4"E), 01 Jul. 2021, leg. J. G. Kim. Dissected paratype (♂) is retained in the collection of I.-H. Kim.

###### Description.

**Figure 22.**
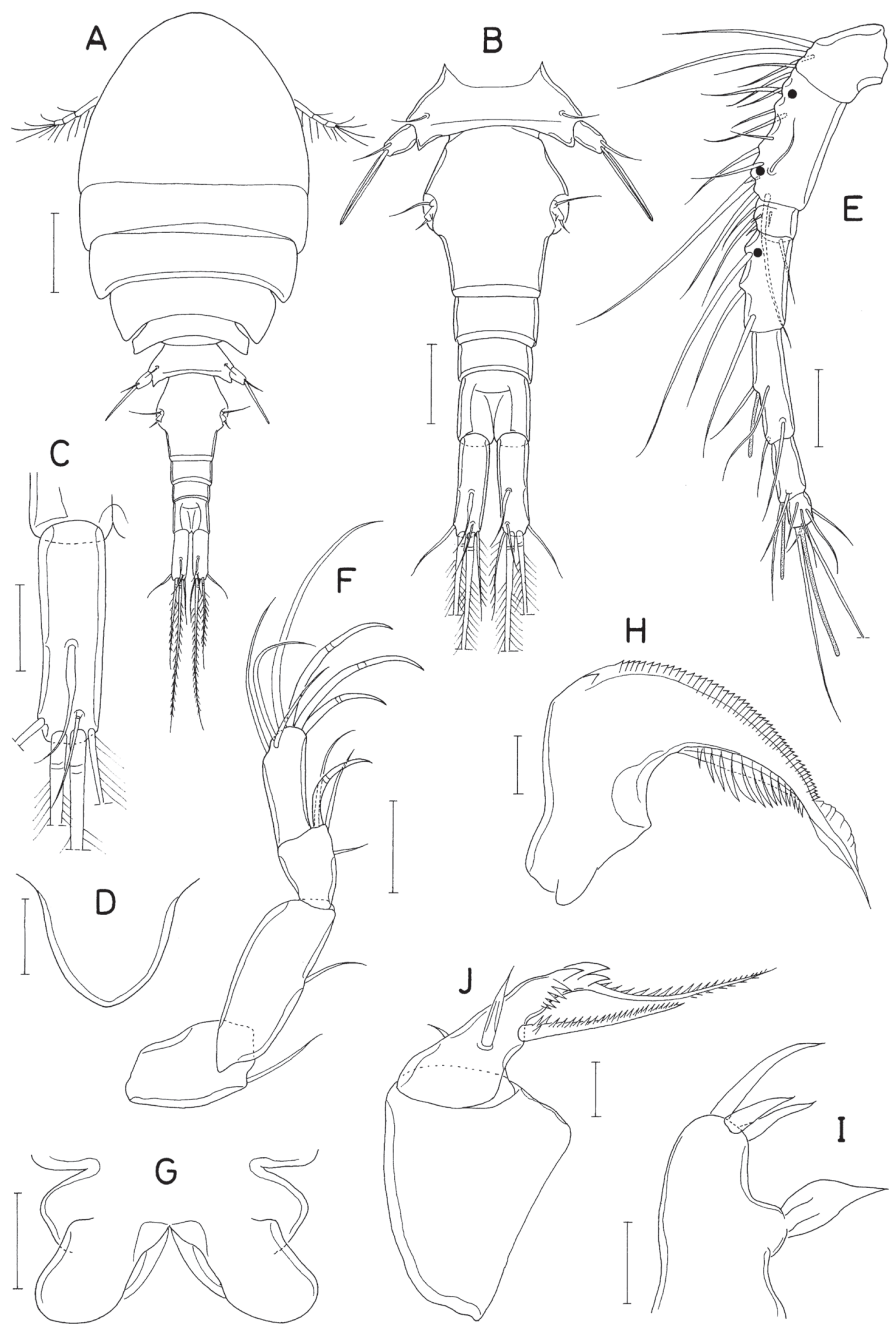
*Eupolymniphilusfoliatus* sp. nov., female **A** habitus, dorsal **B** urosome, dorsal **C** left caudal ramus, dorsal **D** rostrum **E** antennule **F** antenna **G** labrum **H** mandible **I** maxillule **J** maxilla. Scale bars: 0.2 mm (**A**); 0.1 mm (**B**); 0.05 mm (**C–G**); 0.02 mm (**H–J**).

**Female.** Body (Fig. 22A) moderately broad. Body length 1.44 mm. Prosome 840 × 586 μm, fusiform. Cephalothorax with dorsal suture line delimiting cephalosome and first pedigerous somite. Second to fourth pedigerous somites bearing angular posterolateral corners. Urosome (Fig. 22B) five-segmented. Fifth pedigerous somite expanded laterally, wider than genital double-somite, with sleeve-like, pronounced posterolateral corners. Genital double-somite longer than wide (210 × 184 μm), with convex lateral margins, widest at 45% region of double-somite. Three free abdominal somites 59 × 106 μm, 45 × 95 μm, and 80 × 91 μm, respectively. Anal somite unornamented, lacking any spinules. Caudal ramus (Fig. 22C) 3.33 × longer than wide (130 × 39 μm), ~ 1.6 × longer than anal somite, armed with six setae; seta II slightly expanded along proximal third, positioned dorsally at 56% region of ramus length.; setae IV–VI pinnate, other setae naked.

Rostrum (Fig. 22D) well-developed, slightly wider than long, with blunt apex. Antennule (Fig. 22E) 340 μm long, seven-segmented; first and second segments broader than distal segments; armature formula 4, 13, 6, 3, 4+aesthetasc, 2+aesthetasc, and 7+aesthetasc; all setae naked; third and terminal segments equally short. Antenna (Fig. 22F) four-segmented; armature formula 1, 1, 3+claw, and 4+3 claws; terminal segment (third endopodal segment) 2.65 × longer than wide (61 × 23 μm); claws on third and terminal segments slender, setiform; apical seta on terminal segment distinctly longer than other setae on same segment; innermost of three claws on terminal segment shorter than others.

**Figure 23.**
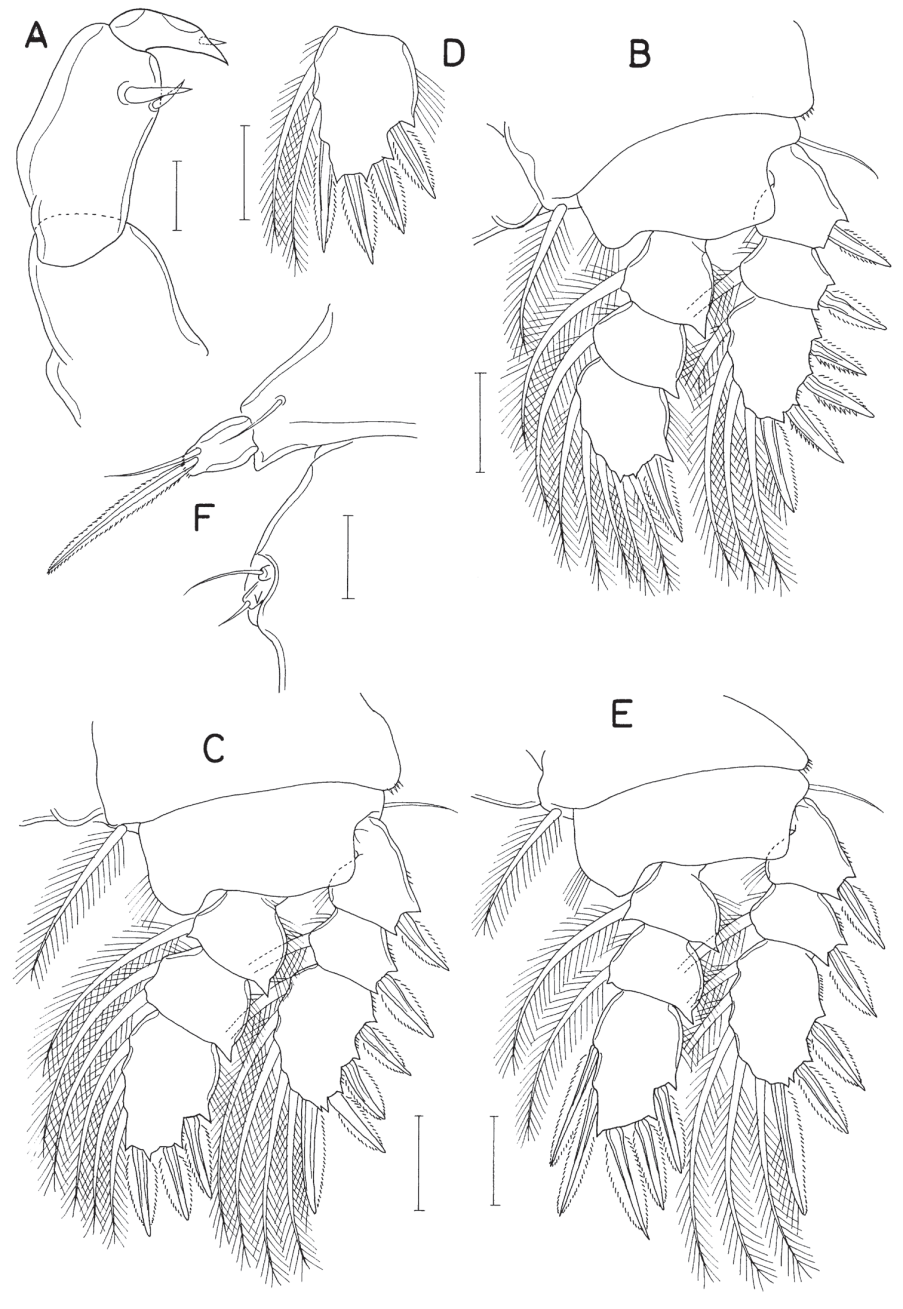
*Eupolymniphilusfoliatus* sp. nov., female **A** maxilliped **B** leg 1 **C** leg 2 **D** third endopodal segment of leg 3 **E** leg 4 **F** left leg 5 and genital aperture. Scale bars: 0.02 mm (**A**); 0.05 mm (**B–F**).

Labrum (Fig. 22G) with distinctly defined, divergent posterolateral lobes and broad posteromedian incision. Mandible (Fig. 22H) with gnathobase bearing finely denticulate convex outer margin, ~ 15 unequal spinules along concave inner margin, and distal lash fringed with wrinkled membrane along outer margin and narrow membrane along inner margin; inner proximal region of gnathobase lacking notch; outer proximal region of blade with one small, indistinct scale. Maxillule (Fig. 22I) lobate, with one expanded, leaf-like, modified seta on inner margin and three (one longer and two shorter) apical setae. Maxilla (Fig. 22J) two-segmented; proximal segment (syncoxa) unarmed; distal segment (basis) distally with five spinules followed by three larger spinules and slender, spinulose lash, and armed with three setae (seta I-III); seta I (inner seta) large, spinulose along distal (outer) margin; seta II (anterior seta) slightly broadened, with acute distal tip; seta III (outer proximal seta) rudimentary. Maxilliped (Fig. 23A) three-segmented; first segment unarmed; second segment with two unequal setae subdistally; third segment narrow, pointed distally, with one small, subdistal seta.

Legs 1–4 (Fig. 23B–D) biramous with three-segmented rami; outer seta on basis small, naked. Inner coxal seta of all swimming legs well-developed, pinnate. Armature formula for legs 1–4 as follows:

**Table:** 

	Coxa	Basis	Exopod	Endopod
Leg 1	0-1	1-0	I-0; I-1; III, I, 4	0-1; 0-1; I, 1, 4
Leg 2	0-1	1-0	I-0; I-1; III, I, 5	0-1; 0-2; I, II, 3
Leg 3	0-1	1-0	I-0; I-1; III, I, 5	0-1; 0-2; I, II, I+2
Leg 4	0-1	1-0	I-0; I-1; II, I, 5	0-1; 0-1; I, II, II

Leg 5 (Fig. 23F) consisting of one small dorsolateral seta on fifth pedigerous somite and exopod; exopodal segment small, 1.59 × longer than wide (46 × 29 μm), widest at proximal third, narrowing distally, armed with one seta (60 μm long) and one elongate compound spine (117 μm long). Leg 6 (Fig. 23F) represented two small setae and single denticle on genital operculum

**Figure 24.**
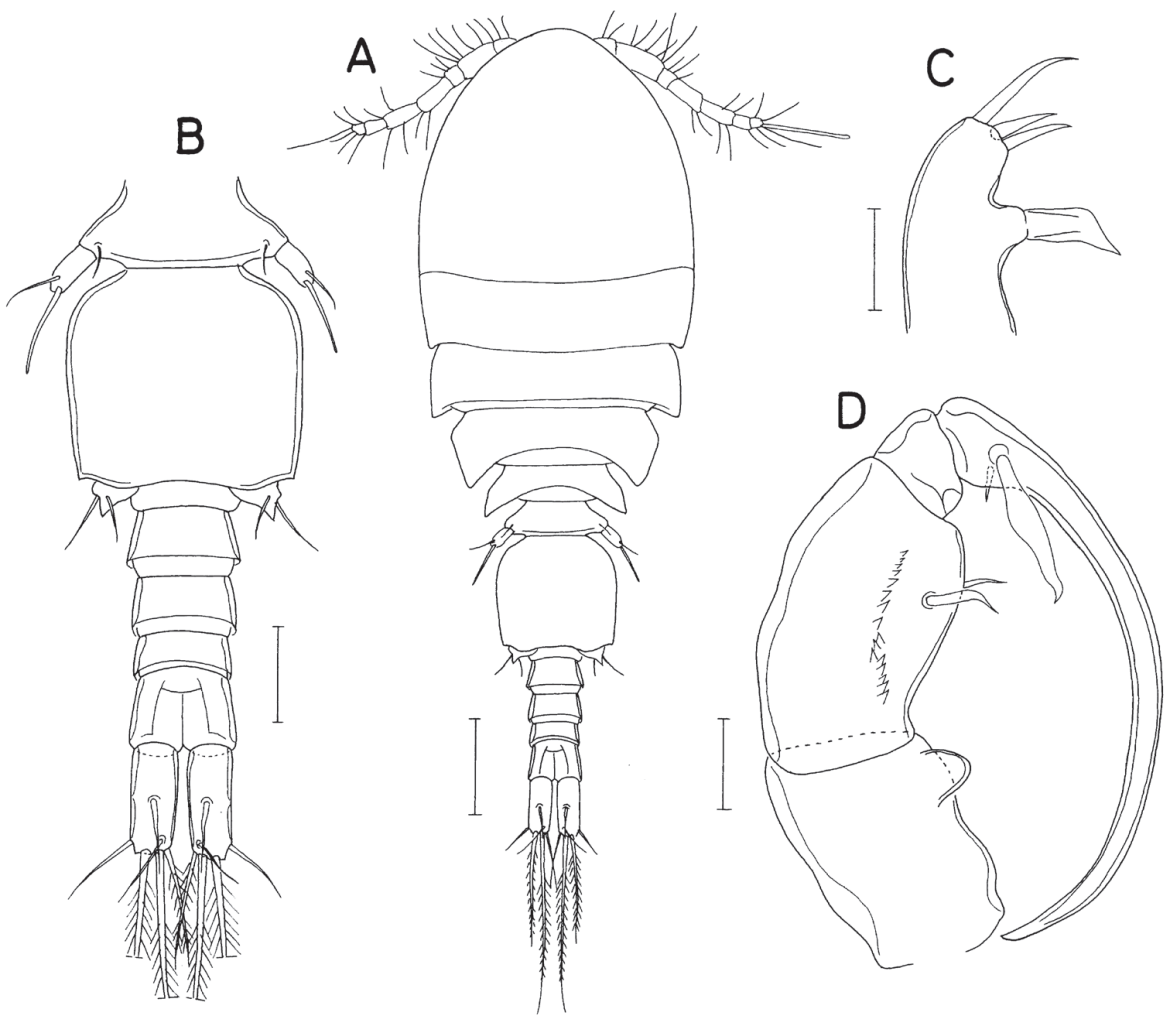
*Eupolymniphilusfoliatus* sp. nov., male **A** habitus, dorsal **B** urosome, dorsal **C** maxillule **D** maxilliped. Scale bars: 0.1 mm (**A**); 0.05 mm (**B**); 0.02 mm (**C, D**).

**Male.** Body (Fig. 24A) narrower and smaller than that of female. Body length 847 μm in dissected paratype (length range 782–847 μm). Prosome 495 × 287 μm. Urosome (Fig. 24B) six-segmented. Fifth pedigerous somite 109 μm wide, lacking posterolateral sleeve-like extension seen in female. Genital somite subquadrate, 127 × 124 μm, with rounded anterolateral corners and pointed posterolateral corners; genital operculum with pointed apex. Four abdominal somites 36 × 60 μm, 29 × 55 μm, 22 × 51 μm, and 36 × 56 μm, respectively. Caudal ramus 2.40 × longer than wide (60 × 25 μm), armed as in female.

Rostrum as in female. Antennule as in female, but with three additional aesthetascs at places of dark circles in Fig. 21E. Antenna, labrum, mandible as in female. Maxillule (Fig. 24C) with less expanded inner margin seta. Maxilla as in female. Maxilliped (Fig. 24D) consisting of three segments and terminal claw; first segment with one large tubercle at inner subdistal region; second segment with two unequal setae and one longitudinal row of spinules; small third segment unarmed; terminal claw elongate, as long as three segments, arched, bearing one setule and one large, slightly undulated seta proximally.

Legs 1–5 as in female. Leg 6 represented by two small setae on genital operculum (Fig. 24B).

###### Etymology.

The specific name of the new species is from Latin *foli* (a leaf), alluding to the leaf-like inner seta of the maxillule.

###### Remarks.

Differences between species of *Eupolymniphilus* are slight. However, *E.foliatus* sp. nov. can be differentiated from its congeners by the key character, the leaf-like modified inner seta of the maxillule. This seta in other species of the genus is known to be simple and slender. Another characteristic feature of the new species is the presence of thick membranes on the distal part of the mandibular lash.

The length-to-width ratio of the caudal ramus in *Eupolymniphilus* is somewhat variable among congeneric species. In the female, it is 3.5:1 in *E.finmarchicus* (Scott T., 1903) according to the illustration of G. O. Sars (1918), ~ 10:1 in *E.tenuicaudis* (G. O. Sars, 1918), 1.50:1 in *E.orientalis* Kim, 2006, 1.03:1 in *E.brevicaudatus* Kim, 2009, 2.69:1 in *E.occidentalis* Kim, 2009, and 3.05:1 in *E.mediterraneus* Costanzo, Brugnano & Zagami, 2013. Thus, *E.foliatus* sp. nov., in which the caudal ramus is 3.33 × longer than wide, is comparable to the three species, *E.finmarchicus*, *E.occidentalis*, and *E.mediterraneus*. Furthermore, they differ from the new species, as follows: *E.finmarchicus* has five setae on the first segment of the antennule (Bocquet et al. 1963), and the mandible lacks any outer scale; *E.occidentalis* has acutely pointed posterolateral corners on the second pedigerous somite (cf. with blunt posterolateral corners in *E.foliatus* sp. nov.), seven aesthetascs on the male antennule (cf. six aesthetascs in *E.foliatus* sp. nov.), and a shorter terminal segment of the antenna which is 1.92 × longer than wide according to Kim (2009) (cf. 2.65 × longer than wide in *E.foliatus* sp. nov.); and *E.mediterraneus* has a small body size, 0.75 mm in the female, and the terminal segment of the antenna bears four claws (Costanzo et al. 2013) (cf. three claws in *E.foliatus* sp. nov.).

### ﻿Family Taeniacanthidae Wilson C.B., 1911

#### Genus *Anchistrotos* Brian, 1906

##### ﻿ Anchistrotos kojimensis

Taxon classificationAnimaliaCyclopoidaTaeniacanthidae

Do & Ho, 1983

FFEC8BE1-3C6F-5A9C-A2BF-1FA7E6E1A784

###### Material examined.

Two ♀♀, Site 31, 11 Nov. 2020.

###### Remarks.

This is a fish-parasitic copepod, living in the gill cavity of the host. Known hosts of this copepod are the gobiid fishes *Acanthogobiusflavimanus* (Temminck & Schlegel, 1845) and *A.hasta* (Temminck & Schlegel, 1845).

**Order Siphonostomatoida Burmeister, 1835**

### ﻿Family Artotrogidae Brady, 1990

#### Genus *Artotrogus* Boeck, 1859

##### ﻿ Artotrogus acutus

Taxon classificationAnimaliaSiphonostomatoidaArtotrogidae

Kim, 1996

955E5926-FC7B-5B2B-BC51-7D591E0C5815

###### Material examined.

One ♂, Site 4, 19 Jul. 2016; 1 ♀, 1 ♂, Site 11, 03 Jun. 2019; 2 ♂♂, Site 11, 10 Jun. 2020.

#### Genus *Ascidipontius* Kim I.H., 1996

##### ﻿ Ascidipontius rarus

Taxon classificationAnimaliaSiphonostomatoidaArtotrogidae

Kim, 1996

AAE7EFD1-D380-5570-A278-4D06FE9D1D15

###### Material examined.

One ♀, 1 ♂, Site 11, 10 Jun. 2020; 2 ♀♀, 1 ♂, Site 21, 26 Apr. 2021.

#### Genus *Bradypontius* Giesbrecht, 1895

##### ﻿ Bradypontius halocynthiae

Taxon classificationAnimaliaSiphonostomatoidaArtotrogidae

Kim, 1996

07421D6D-902D-52E8-8D83-141B42FC979B

###### Material examined.

One ♀, 1 ♂, Site 11, 10 Jun. 2020.

#### Genus *Cryptopontius* Giesbrecht, 1899

##### ﻿ Cryptopontius ascidius

Taxon classificationAnimaliaSiphonostomatoidaArtotrogidae

Kim, 1996

9C4C8A1C-098F-563A-A2DC-856805DBB003

###### Material examined.

One ♀, Site 11, 03 Jun. 2019; 5 ♀♀, Site 11, 10 Jun. 2019; 1 ♀, 10 ♂♂, Site 11, 10 Jun. 2020.

##### ﻿ Cryptopontius donghaensis

Taxon classificationAnimaliaSiphonostomatoidaArtotrogidae

Kim, 1996

1F43CA98-A5D8-5640-ACB6-0139D59C5E1B

###### Material examined.

One ♀, 5 ♂♂, Site 1, 28 Jun. 2021; 1 ♀, 4 ♂♂, Site 2, 01 Jul. 2021; 1 ♀, Site 3, 17 Jul. 2016; 10 ♀♀, 41 ♂♂, Site 7, 19 Jul. 2016; 2 ♀♀, 5 ♂♂, Site 12, 16 Mar. 2013; 1 ♀, 4 ♂♂, Site 14, 04 Jul. 2020.

###### Remarks.

This species is the most frequently found artotrogid copepod in Korean waters; living on sponges, among sea weeds, and on submerged fishing nets in ports (Kim 2010a).

#### Genus *Pteropontius* Giesbrecht, 1895

##### ﻿ Pteropontius trimerus

Taxon classificationAnimaliaSiphonostomatoidaArtotrogidae

Kim, 1996

29B2EF8D-064D-5E86-954D-7B1161E30B39

###### Material examined.

Five ♀♀, Site 33, 11 Aug. 2020.

###### Remarks.

This copepod had been found only on the external surface of the tunicate *Halocynthiaigaboja* Oka, 1906.

### ﻿Family Asterocheridae Giesbrecht, 1899

#### Genus *Acontiophorus* Brady, 1880

##### ﻿ Acontiophorus estivalis sp. nov.

Taxon classificationAnimaliaSiphonostomatoida Asterocheridae

EDC2C572-4DF3-5AF8-BA49-927F82CAB8A0

https://zoobank.org/5654B945-0217-4D32-A0EB-2BF6D204BD4E

Figs 25 , 26 , 27

###### Material examined.

***Holotype*** ♀ (MABIK CR00250115) and ***paratypes*** 3 ♀♀, 2 ♂♂ (MABIK CR00250116) preserved in 90% alcohol, and ***paratypes*** 1 ♀, 1 ♂ dissected and mounted on a slide, Site 11 (Yeongdo, Pusan, 35°04'31.0"N, 129°05'08.7"E), 07 Jul. 2020, leg. J. G. Kim. Dissected paratypes (1 ♀, 1 ♂) are retained in the collection of I.-H. Kim.

###### Description.

**Figure 25.**
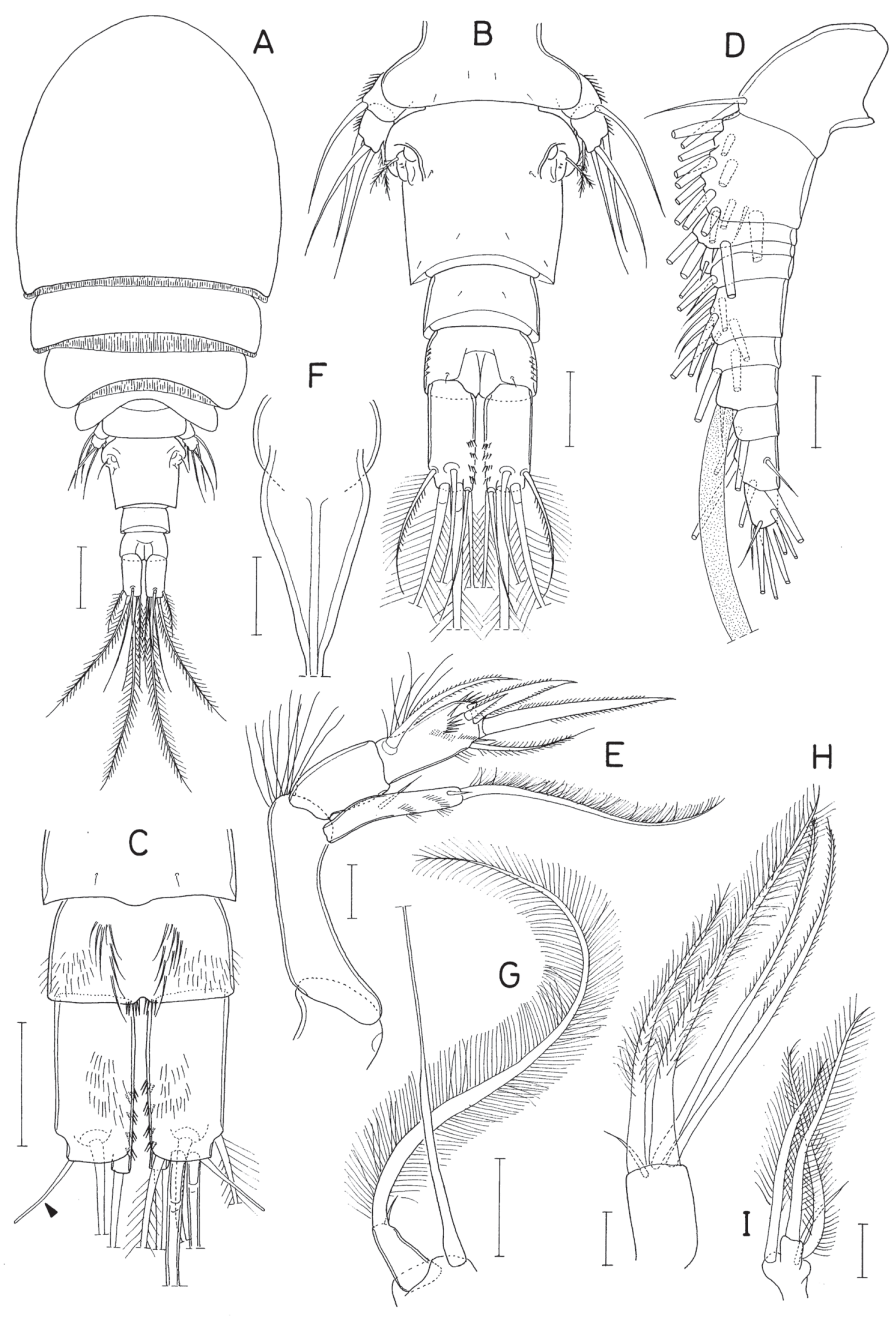
*Acontiophorusestivalis* sp. nov., female **A** habitus, dorsal **B** urosome, dorsal **C** anal somite and caudal rami, ventral **D** antennule **E** antenna **F** oral siphon **G** mandible **H** inner lobe of maxillule **I** outer lobe of maxillule. Scale bars: 0.1 mm (**A**); 0.05 mm (**B, C, F, G**); 0.02 mm (**D, E, H, I**).

**Female.** Body (Fig. 25A) stout, 938 μm long in dissected and figured paratype (length range 893–945 μm, holotype 945 μm). Prosome 625 × 425 μm, occupying 67% of body length, consisting of cephalothorax and second to fourth pedigerous somites. Cephalothorax 425 μm long, as long as wide, without any dorsal suture line delimiting cephalosome and first pedigerous somite. Cephalothorax and second to third pedigerous somites with membranous fringe along posterodorsal margin. Fourth pedigerous somite with deeply concave posterior margin. Urosome (Fig. 25B) four-segmented. Fifth pedigerous somite 133 μm wide, with round lateral margins. Genital double-somite wider than long (115 × 132 μm), consisting of laterally expanded anterior third and narrower posterior two-thirds, with pointed posterolateral corners; genital apertures positioned dorsolaterally at expanded anterior region. Two free abdominal somites 39 × 77 μm and 45 × 75 μm. Anal somite (Fig. 25C) ornamented on ventral surface with two groups of several large setules on medial region, scattered fine setules on lateral regions, and several spinules at medial posterior margin near bases of caudal rami. Caudal ramus (Fig. 25C) rectangular, 2.12 × longer than wide (70 × 33 μm), armed with six setae plus one aesthetasc-like element (indicated by arrowhead in Fig. 25C), and ornamented with fine setules on ventral surface and four transverse rows of minute spinules on inner margin; dorsal setae (setae VI and VII) naked, other setae pinnate; outer lateral seta (seta II) with long setules along outer margin but spinulose (or with short setules) along inner margin; seta VI inserted on prolongation of ramus.

Rostrum absent. Antennule (Fig. 25D) short, 147 μm long, 11-segmented; armature formula 2, 14, 4, 2, 2, 8, 2, 1+aesthetasc, 2, 4, and 7; aesthetasc on 8^th^ segment large; setae densely arranged, difficult to distinguish from one another. Antenna (Fig. 25E) consisting of coxa, basis, one-segmented exopod, and two-segmented endopod; coxa short, unarmed; basis longest segment, narrowed in mid-region, with tuft of long setules at inner distal corner; exopod elongate, 6.0 × longer than wide (54 × 9 μm), extending to middle of second endopodal segment, armed with one small seta in middle, one minute seta subdistally, and one large, unilaterally pinnate seta (97 μm long) distally; first endopodal segment unarmed, 32 × 22 μm; second endopodal segment 2.2 × longer than wide (40 × 18 μm), armed with six setae consisting of one large proximal seta, three unequal subdistal setae (one minute, setule-like), and two broad apical setae 75 and 43 μm long, and ornamented with several rows of fine spinules or setules.

**Figure 26.**
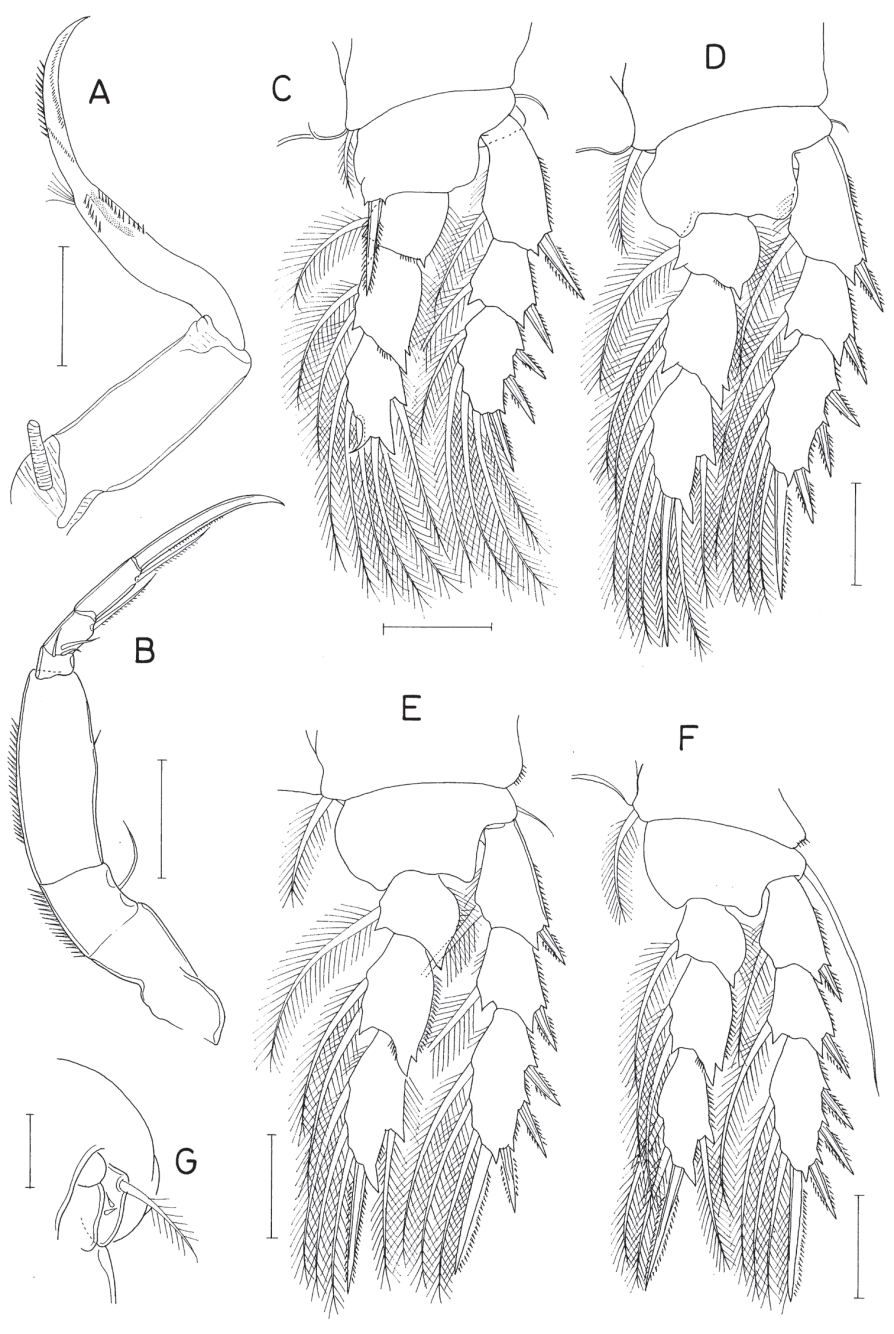
*Acontiophorusestivalis* sp. nov., female **A** maxilla **B** maxilliped **C** leg 1 **D** leg 2 **E** leg 3 **F** leg 4 **G** right genital aperture, dorsal. Scale bars: 0.05 mm (**A–F**); 0.02 mm (**G**).

Oral siphon (Fig. 25F) consisting of conical proximal part (maximum width 67 μm) and thin distal part, extending to middle of genital double-somite. Mandible (Fig. 25G) consisting of thread-like stylet and palp; palp short, tapering, armed with one large, heavily pinnate seta and one minute, setule-like seta distally. Maxillule (Fig. 25H, I) bilobed; larger inner lobe armed with four large (two feebly pinnate and two plumose) and one small setae distally; smaller inner lobe armed with three pinnate and one small, naked setae. Maxilla (Fig. 26A) slender, two-segmented; proximal segment (syncoxa) unarmed, basally with short tube of maxillary gland; distal segment (basis) forming long claw, longer than proximal segment, ornamented with rows of small spinules and one tuft of few setules. Maxilliped (Fig. 26B) five-segmented, consisting of syncoxa, basis, three-segmented endopod, and terminal claw; syncoxa with trace of articulation delimiting praecoxal and coxal regions, coxal region with one seta on inner margin and row of spinules along outer margin; basis with one rudimentary seta at distal third of inner margin and row of spinules along outer margin; three endopodal segments armed with two, two, and one setae, respectively; terminal claw weakly curved, 66 μm long, more than twice longer than third endopodal segment (31 μm long).

Legs 1–4 (Fig. 26C–F) biramous, with three-segmented rami. Inner coxal seta well-developed in legs 1–4; outer seta on basis small in legs 1–3, but markedly large in leg 4. Inner distal spine on basis of leg 1 extending to middle of second endopodal segment. Second endopodal segment of legs 1–4 with bicuspid outer distal corner. Inner distal process of third endopodal segment of leg 1 acutely pointed. Armature formula for legs 1–4 as follows:

**Table:** 

	Coxa	Basis	Exopod	Endopod
Leg 1	0-1	1–I	I-1; I-1; II, I, 5	0-1; 0-2; 1, 2, 3
Leg 2	0-1	1-0	I-1; I-1; III, I, 4	0-1; 0-2; 1, 1+I, 3
Leg 3	0-1	1-0	I-1; I-1; III, I, 3	0-1; 0-2; 1, I, 3
Leg 4	0-1	1-0	I-1; I-1; III, I, 3	0-1; 0-2; 1, I, 2

**Figure 27.**
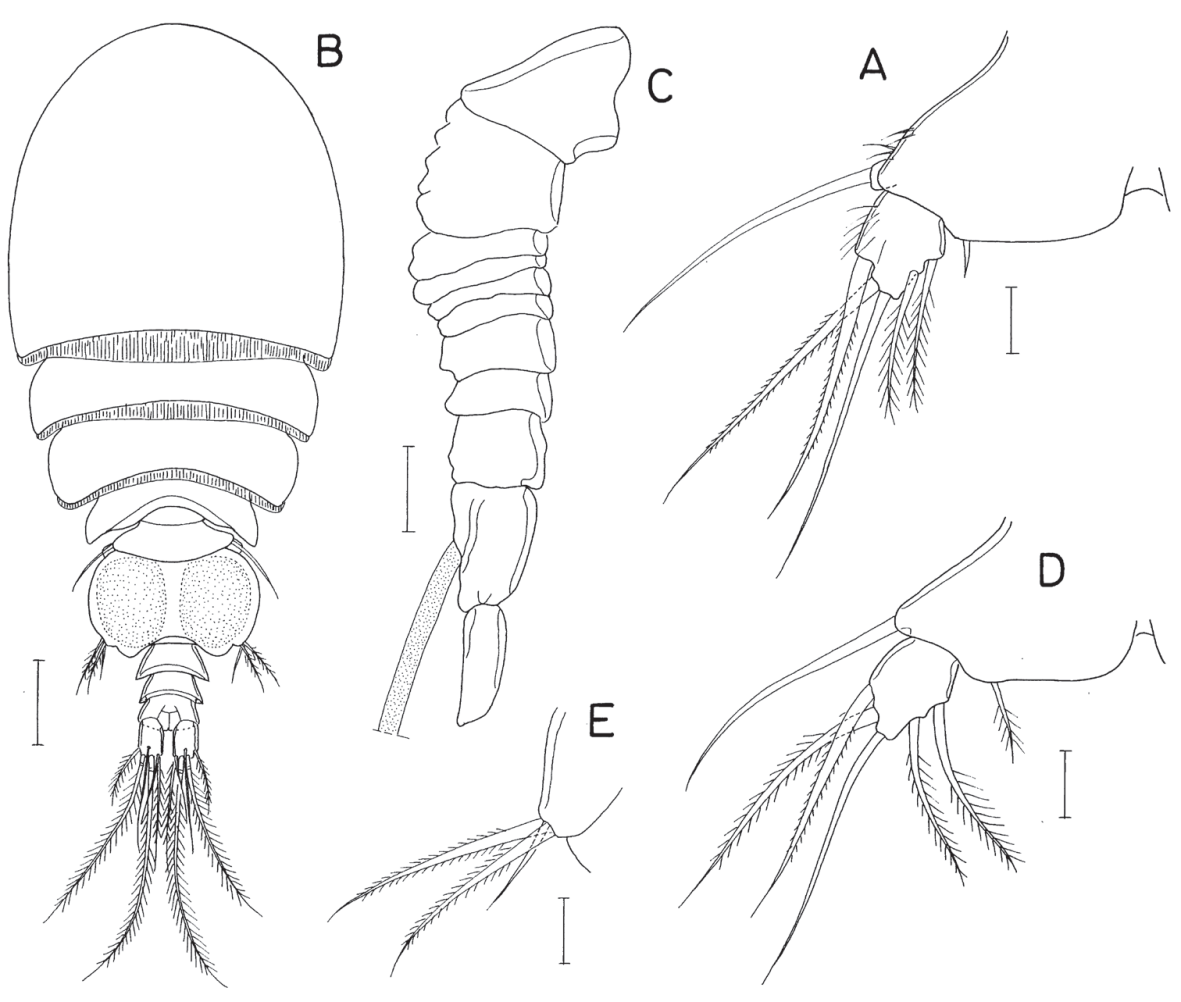
*Acontiophorusestivalis* sp. nov., female **A** leg 5, dorsal. Male **B** habitus, dorsal **C** antennule (setae omitted) **D** leg 5, dorsal **E** leg 6. Scale bars: 0.02 mm (**A, C–E**); 0.1 mm (**B**).

Leg 5 (Fig. 27A) two-segmented. First segment (protopod) broad, not articulated from somite, armed with large, naked outer distal seta and small, naked inner distal seta. Distal segment (exopod) 1.12 × longer than wide (28 × 25 μm), armed with five setae, and ornamented with fine setules on outer surface; two smaller setae on inner margin pinnate, terminal seta naked, two outer setae feebly pinnate. Leg 6 (Fig. 26G) represented by one pinnate seta and two minute setules on genital operculum.

**Male.** Body form (Fig. 27B) as in female. Body length 890 μm in dissected and figured paratype (length range 821–890 μm). Prosome 600 μm long. Cephalothorax slightly wider than long (374 × 407 μm). Urosome five-segmented, Genital somite much wider than long. First two free abdominal somites broadened distally, with pointed posterolateral corners. Caudal ramus 1.44 × longer than wide (46 × 32 μm), armed as in female.

Antennule (Fig. 27C) 168 μm long, 11-segmented; setae entangled, difficult to distinguish from one another; aesthetascs five on second segment, two on third, one on each 7^th^ and 10^th^ segment; aesthetasc on 10^th^ segment large. Antenna as in female.

Oral siphon, mandible, maxillule, maxilla, maxilliped, and legs 1–4 same as those of female. Leg 5 (Fig. 27D) also shaped as in female; inner distal seta on protopod pinnate; exopodal segment 1.33 × longer than wide (32 × 24 μm), armed as in female. Leg 6 (Fig. 27E) represented by three setae (two larger, weakly pinnate and one smaller naked) on genital operculum.

###### Etymology.

The specific name *estivalis* is derived from Latin *estival* (summer), indicating the discovery of the new species in the summer.

###### Remarks.

The segmentation of the antennule appears to be a reliable character for the differentiation of *Acontiophorus* species. *Aconiophorusestivalis* sp. nov. has an 11-segmented antennule in the female; this feature is shared with three congeners, *A.antennatus* Hansen, 1923, *A.scutatus* (Brady & Robertson, 1873) and *A.zealandicus* Sewell, 1944. *Acontiphorusantennatus* was redescribed by Eiselt (1969) and according to his illustration and description, the caudal ramus of *A.antennatus* is ~ 4 × longer than wide in the female (cf. 2.12 × longer than wide in *A.estivalis* sp. nov.) and the third exopodal segment of leg 1 is armed with three spines and four setae (formula III, 4; against III, 5 in *A.estivalis* sp. nov.). In *A.zealandicus* the male antennule is ten-segmented (cf. 11-segmented in *A.estivalis* sp. nov.) and the oral siphon is extremely long, extending beyond the caudal rami (Nicholls 1944) (cf. extending to middle of genital double-somite in A.estivalis sp. nov.). Therefore, *A.antennatus* and *A.zelandicus* can be distinguished from *A.estivalis* sp. nov. with confidence. *Acontiophorusestivalis* sp. nov. closely resembles *A.scutatus*. As noticeable differences between them, the caudal ramus of *A.scutatus* is 3 × longer than wide, and the oral siphon of the latter species extends to the caudal rami (Sars 1915) (cf. the siphon extends to the middle of the genital double-somite in *A.estivalis* sp. nov.). Additionally, the first segment of the male antennule of *A.scutatus* bears an aesthetasc, according to the illustration of Sars (1915), which is absent in *A.estivalis* sp. nov.

#### Genus *Dermatomyzon* Claus, 1889

##### ﻿ Dermatomyzon nigripes

Taxon classificationAnimaliaSiphonostomatoidaAsterocheridae

(Brady & Robertson, 1880)

0CEEC9D9-AAD8-5821-8A11-D116466BCF71

###### Material examined.

Two ♂♂, Site 1, 28 Jun. 2021; 1 ♀, Site 3, 17 July. 2016; 1 ♀, Site 4, 03 Jun. 2019; 2 ♀♀, Site 5, 21 Jun. 2016; 1 ♀, Site 6, 21 Sep. 2020; 1 ♀, 2 ♂♂, Site 11, 20 Aug. 2020; 2 ♀♀, 1 ♂, Site 13, 03 Jul. 2020; 6 ♀♀, 6 ♂♂, Site 16, 04 Jul. 2020.

###### Remarks.

*Dermatomyzonnigripes* is a cosmopolitan species, and has frequently been collected in Korean coasts. The host of this copepod is still unknown.

#### Genus *Thermocheres* Kim I.H., 2010

##### ﻿ Thermocheres pacificus sp. nov.

Taxon classificationAnimaliaSiphonostomatoidaAsterocheridae

D5294558-6A7E-5C1C-8A97-17ABD371518B

https://zoobank.org/5F56B3F1-9731-49D8-A209-C6945F094AEA

Figs 28 , 29

###### Material examined.

***Holotype*** ♂ (MABIK CR00250117) preserved in 90% alcohol, Site 22 (Yesong, Bogil Island, south coast, 34°08'11"N, 126°33'49"E), 26 Apr. 2021, leg. J. Lee and C. Y. Chang; ***Paratype*** ♂ dissected and mounted on a slide, Site 15 (Namhae Island, south coast, 34°45'00.5"N, 127°54'33.9"E), 04 Jul. 2020, leg. J. G. Kim. Dissected paratype is retained in the collection of I.-H. Kim.

###### Description.

**Figure 28.**
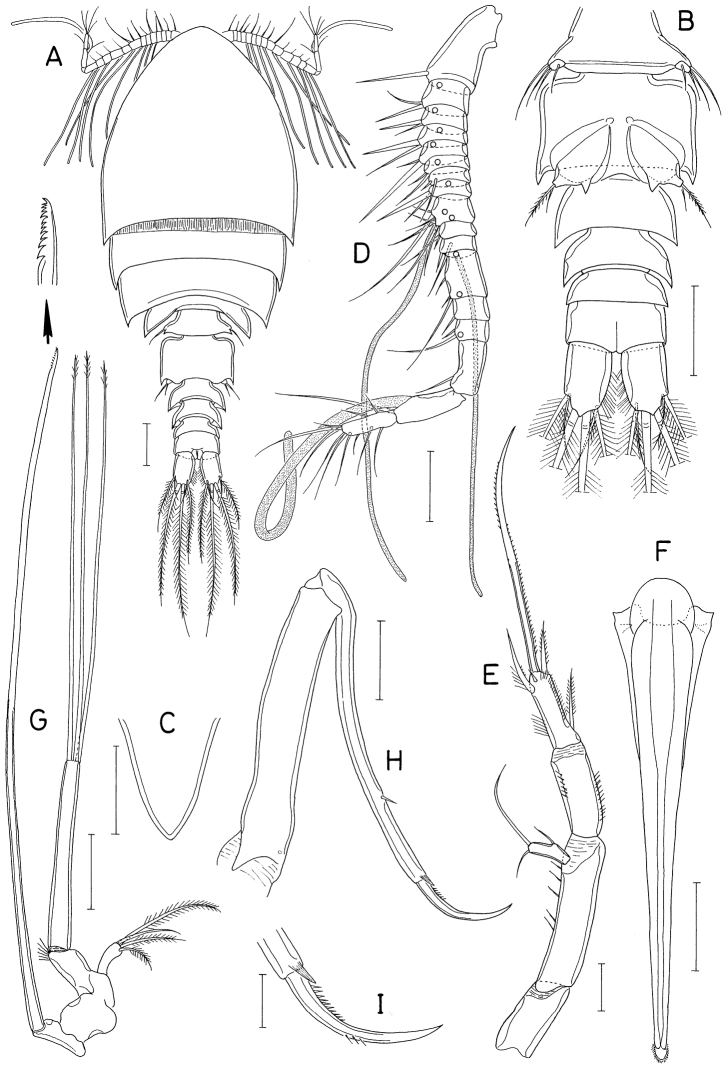
*Thermocherespacificus* sp. nov., male **A** habitus, dorsal **B** urosome, ventral **C** rostrum **D** antennule (open circles indicate insertions of aesthetascs on opposite surface) **E** antenna **F** oral siphon **G** mandible and maxillule **H** maxilla **I** distal part of maxilla. Scale bars: 0.1 mm (**A, B, F**); 0.05 mm (**C, D, G, H**); 0.02 mm (**E, I**).

**Male.** Body (Fig. 28A) cyclopiform, moderately broad. Body length 1.14 mm in dissected and figured paratype (1.25 mm in holotype). Prosome 695 μm long, four-segmented, consisting of cephalothorax and three free pedigerous somites. All prosomal somites with acutely pointed posterolateral corners. Cephalothorax slightly wider than long (477 × 486 μm), consisting of completely fused cephalosome and first pedigerous somite, fringed with membrane along posterodorsal margin. Urosome (Fig. 28B) six-segmented. Fifth pedigerous somite narrower than genital somite, with tapered lateral apex. Genital somite quadrangular, wider than long (125 × 184 μm), with parallel lateral margins and pointed, tooth-like posterolateral corners; genital operculum well-developed, with one large cusp on distal margin and pair of unequal setae on tip of posterolateral apex. Four abdominal somites 57 × 140 μm, 45 × 125 μm, 36 × 116 μm, and 52 × 114 μm, respectively; first and second abdominal somites with acutely pointed, posteriorly extended posterolateral corners. Caudal ramus (Fig. 28B) 1.57 × longer than wide (83 × 53 μm), with six pinnate setae, ornamented with setules along inner margin; all setae positioned distally or subdistally.

Rostrum (Fig. 28C) slightly longer than wide, tapered, with angular apex. Antennule (Fig. 28D) 368 μm long, 17-segmented, geniculate between antepenultimate and penultimate segments; armature formula 1+aesthetasc, 2+aesthetasc, 2+aesthetasc, 2+aesthetasc, 2+aesthetasc, 2, 2+aesthetasc, 2, 7+3 aesthetascs, 2, 2+aesthetasc, 3+aesthetasc, 1, 2+aesthetasc, 1, 1+aesthetasc, and 11; setae naked, mostly short; aesthetascs thin but that of penultimate segment thicker. Antenna (Fig. 28E) consisting of coxa, basis, one-segmented exopod, and two-segmented endopod; coxa 35 × 17 μm, unarmed; basis 65 × 17 μm, with few spinules on outer margin; exopod 3 × longer than wide (18 × 6 μm), armed with two unequal setae distally and one seta near middle; first endopodal segment 38 × 15 μm, unarmed but with row of spinules on inner and outer margins; second endopodal segment 35 × 12 μm, terminated in long spiniform seta (107 μm long), armed with one seta on proximal inner margin, three (one minute) setae distally and subdistally, and ornamented with setules on inner and outer margin.

**Figure 29.**
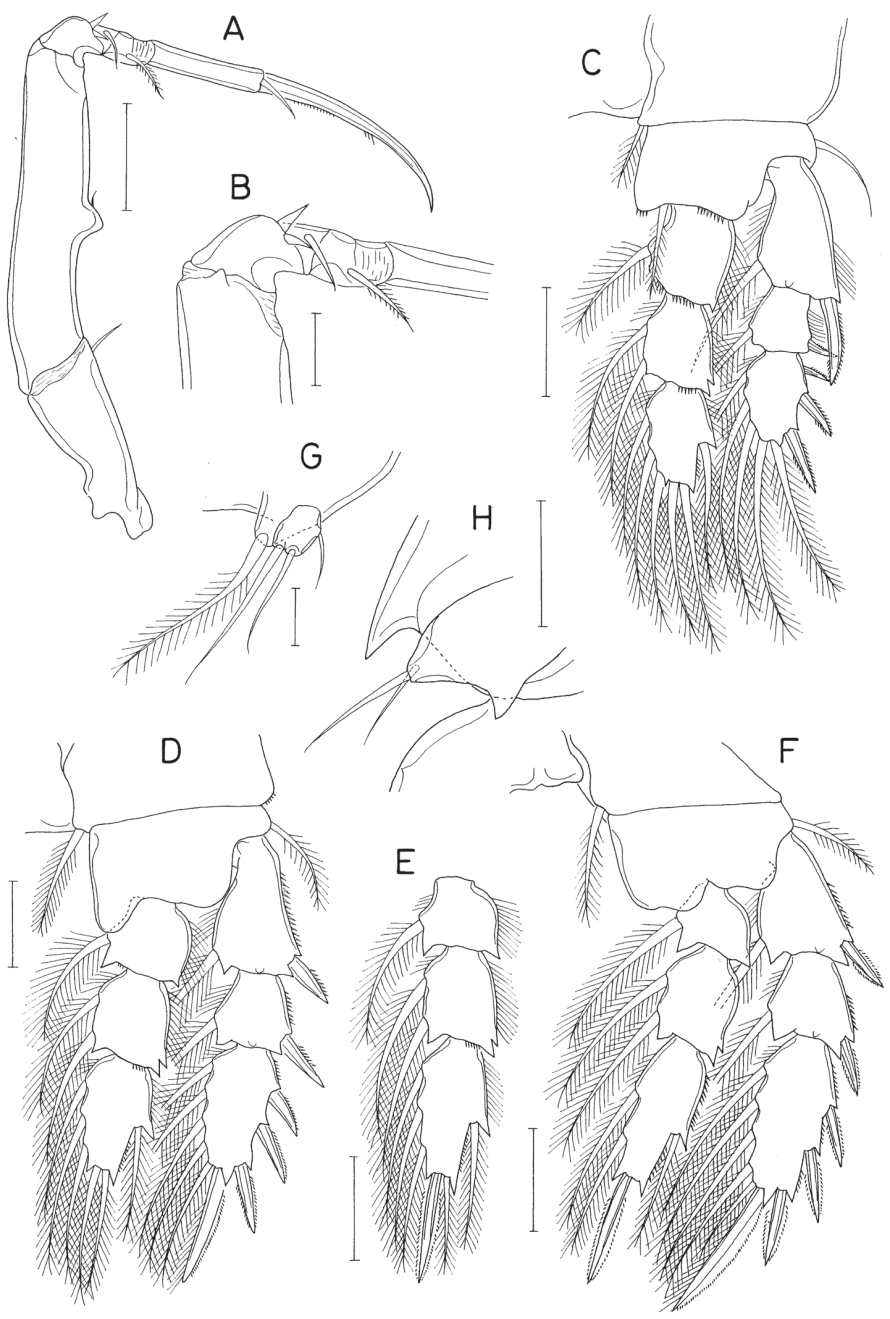
*Thermocherespacificus* sp. nov., male **A** maxilliped **B** endopodal region of maxilliped **C** leg 1 **D** leg 2 **E** endopod of leg 3 **F** leg 4 **G** leg 5 **H** right genital operculum, ventral. Scale bars: 0.05 mm (**A, C–F, H**); 0.02 mm (**B, G**).

Oral siphon (Fig. 28F) 454 μm long, evenly tapering from proximal to distal, extending to insertions of leg 2. Mandible (Fig. 28G) consisting of short basal segment and elongate, slender stylet bearing 11 teeth distally. Maxillule (Fig. 28G) bilobed; small outer lobe 23 × 9 μm, distally with four setae, one naked, other three pinnate; elongate inner lobe based on segment-like extension, 127 × 15 μm, tipped with three thin, equally long, distally feebly pinnate setae. Maxilla (Fig. 28H) slender, consisting of syncoxa (189 μm long), basis (205 μm long) and terminal claw; basis with small seta at 70% region and small, tapering membrane distally; terminal claw (Fig. 28I) 64 μm long, curved, with row of spinules along proximal half of concave margin. Maxilliped (Fig. 29A, B) consisting of syncoxa, basis, four-segmented endopod, and terminal claw; syncoxa with one seta on inner distal corner; basis longest, with one blunt tubercle on inner margin bearing minute setule on distal margin of tubercle; endopodal segments with two, one, one, and one setae respectively; terminal claw slender, 103 μm long, weakly curved, ~ twice longer than terminal endopodal segment.

Legs 1–4 (Fig. 29C–F) biramous, with three-segmented rami. Outer seta on basis naked in leg 1 but pinnate in legs 2–4. Outer spine on first exopodal segment of leg 1 large, extending beyond base of first outer spine of third exopodal segment. Second endopodal segment of legs 1–4 with bicuspid outer distal corner. Inner distal seta on third exopodal segment of leg 4 distinctly smaller than proximal setae. Armature formula for legs 1–4 as follows:

**Table:** 

	Coxa	Basis	Exopod	Endopod
Leg 1	0-1	1-1	I-1; I-1; III, 5	0-1; 0-2; 1, 2, 3
Leg 2	0-1	1-0	I-1; I-1; III, I, 5	0-1; 0-2; 1, 2, 3
Leg 3	0-1	1-0	I-1; I-1; III, I, 5	0-1; 0-2; 1, 1+I, 3
Leg 4	0-1	1-0	I-1; I-1; III, I, 5	0-1; 0-2; 1, I, 2

Leg 5 (Fig. 29G) consisting of pinnate lateral seta on fifth pedigerous somite and small exopod; exopodal segment 18 × 14 μm, articulated from somite, with three naked setae (two on distal margin and one on posterior margin). Leg 6 (Fig. 29H) represented by two naked setae on genital operculum.

**Female.** Unknown.

###### Etymology.

The specific name of the new species refers to its discovery in the Pacific Ocean, in contrast with the Indian Ocean in which the type locality, Madagascar, of the type species is located.

###### Remarks.

The discovery of this new species reinforces the taxonomic status of the genus *Thermocheres*. The type species of the genus, *T.validus* Kim, 2010, was described as an associate of a sponge in Madagascar (Kim 2010b). Although the new species is represented by only a single male, it exhibits diagnostic characters of the genus. In particular, the form of the maxillule in which the inner lobe is elongated and armed with three long, slender setae and the armature condition (formula III, I, 5) of the third exopodal segment of legs 2–4 are shared by the two species. Within the Asterocheridae, the latter character is shared only by *Australomyzon* Nicholls, 1944 and *Bythocheres* Humes, 1988. But *T.pacificus* sp. nov. and *T.validus* reveal two important differences, i.e., (1) one of four setae on the outer lobe of the maxillule is positioned in middle in *T.validus*, whereas all of the four setae are distally positioned in *T.pacificus* sp. nov.; and (2) the third endopodal segment of leg 4 is armed with one spine plus four setae (formula 1, 1+I, 2), but with one spine plus three setae (formula 1, I, 2) in *T.pacificus* sp. nov. These two differences are so significant that the Korean material should be separated from the type species as a new species. The proportional length of the caudal ramus and the developmental condition of the protopod of leg 5 also appear different between the two species, although these characters are subject to sexual dimorphism. *Thermocheresvalidus* was described on the basis of the female only.

### ﻿Family Caligidae Burmeister, 1835

#### Genus *Caligus* Müller O.F., 1785

##### ﻿ Caligus amblygenitalis

Taxon classificationAnimaliaSiphonostomatoidaCaligidae

Pillai, 1961

5F8C75B1-50B1-5CE2-8174-ADE035194577

Figs 30 , 31 , 32

Caligus amblygenigtalis Pillai, 1961: 98, figs 8, 10; Ho and Lin, 2003: 56, figs 1, 2. Caliguslongipedis: Ho and Lin, 2001: 188, fig. 9 (male only).

###### Material examined.

Two ♀♀, 5 ♂♂ (MABIK CR00250989–CR00250995), Site 7, 21 Nov. 2019; 1 ♂, Site 11, 10 Jun. 2019.

###### Description.

**Figure 30.**
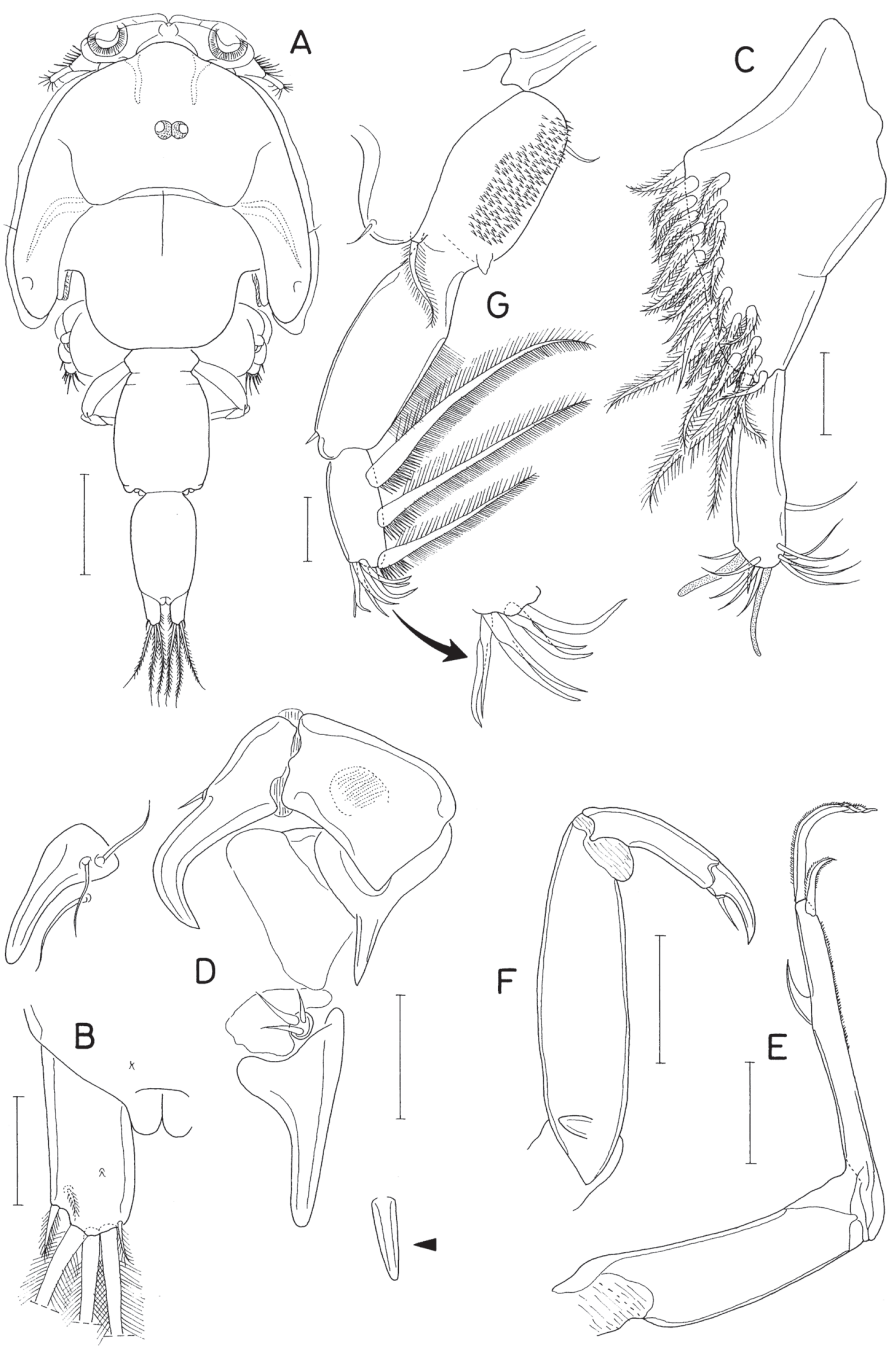
*Caligusamblygenitalis* Shiino, 1961, female **A** habitus, dorsal **B** caudal ramus, dorsal **C** antennule **D** antenna, postantennary process, maxillule, and post-maxillular process (indicated by arrowhead) **E** maxilla **F** maxilliped **G** leg 1. Scale bars: 0.5 mm (**A**); 0.1 mm (**B, D–F**); 0.05 mm (**C, G**).

**Female.** Body (Fig. 30A) 3.06 mm long. Cephalothoracic shield 1.66 × 1.52 mm. Lunules distinct. Thoracic zone of cephalothorax distinctly extending beyond posterior ends of lateral zones. Genital complex longer than wide (619 × 479 μm), nearly rectangular, not clearly articulated from fourth pedigerous somite. Abdomen one-segmented, longer than wide (540 × 330 μm). Caudal ramus (Fig. 30B) 2.09 × longer than wide (167 × 80 μm), with three large and three small setae; one of small setae located on ventral surface of ramus.

Antennule (Fig. 30C) two-segmented; proximal segment 220 μm long, armed with 29 setae, two dorsal setae naked; distal segment 123 μm long, armed with 12 naked setae and two aesthetascs. Antenna (Fig. 30D) three-segmented; first segment with narrow, pointed process; second segment unarmed, with adhesion pad on anterior surface; third segment bearing curved distal claw and one small seta on convex margin. Postantennary process (Fig. 30D) bluntly tipped, with two papillae each bearing unbranched setule; another setule-bearing papilla on sternum posterior to process.

**Figure 31.**
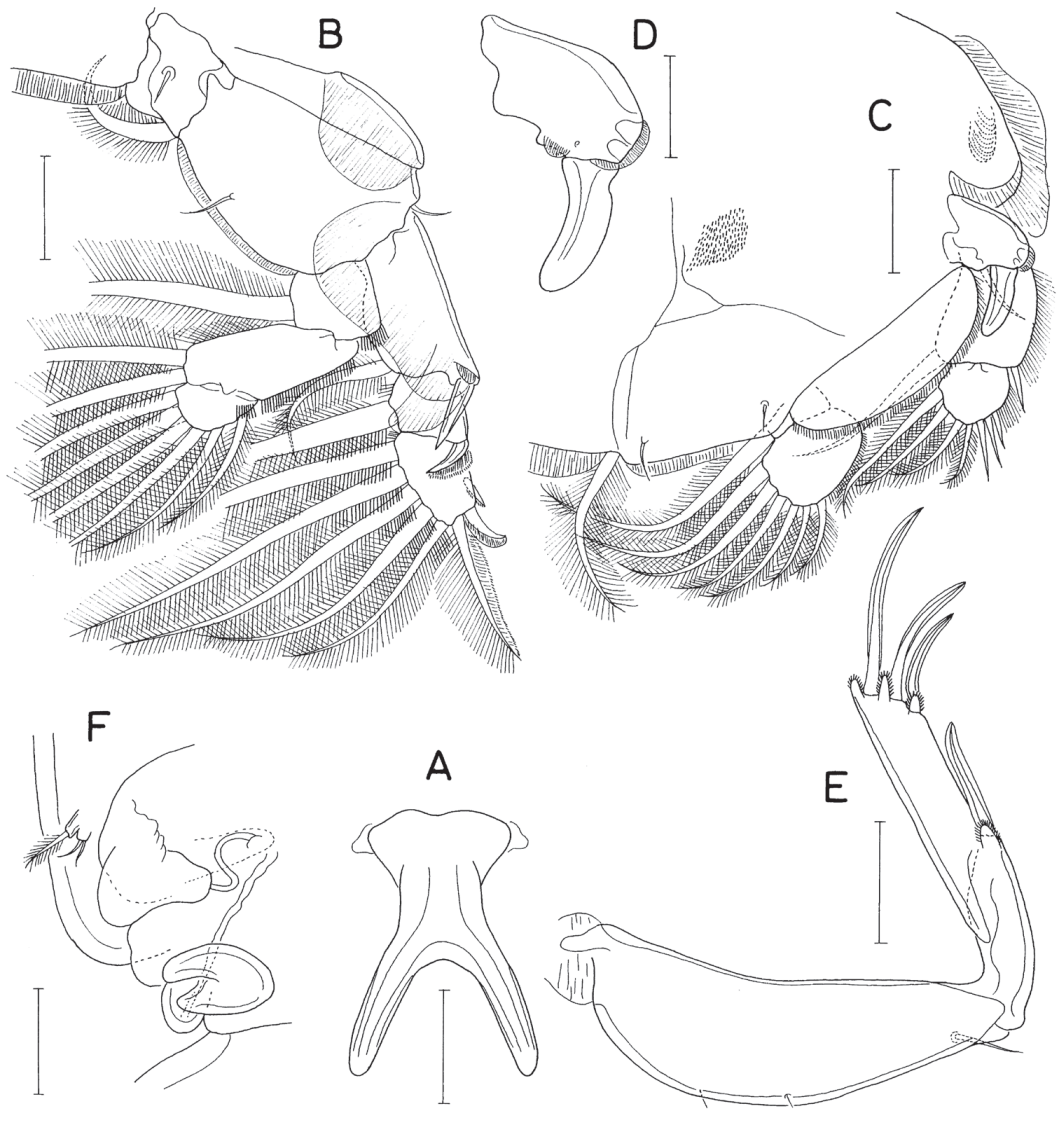
*Caligusamblygenitalis* Shiino, 1961, female **A** sternal furca **B** leg 2 **C** leg 3 **D** first exopodal segment of leg 3 **E** leg 4 **F** right genital area. Scale bars: 0.1 mm (**A–C, E**); 0.05 mm (**D, F**).

Mandible with 12 teeth on distal blade. Maxillule (Fig. 30D) comprising anterior papilla bearing three setae and bluntly tipped posterior process. Post-maxillular process (indicated by arrowhead in Fig. 30D) present postero-medial to maxillule. Maxilla (Fig. 30E) two-segmented; proximal segment unarmed; distal segment slender, bearing hyaline membrane at distal 38% region of segment and distally with short canna and long calamus; distal half of inner margin of distal segment with fine spinules. Maxilliped (Fig. 30F) slender, consisting of two segments and terminal claw; proximal segment proximally with sclerotized process; distal segment less than half length of proximal segment, unarmed; terminal claw short, proximally with one small seta. Sternal furca (Fig. 31A) with widely divergent, narrow tines.

Leg 1 (Fig. 30G) consisting of coxa, basis, two-segmented exopod and rudimentary endopod; basis with two setae (one outer and one medio-distal) and large patch of spinules on ventral surface; proximal exopodal segment with one small subdistal seta on outer margin; distal exopodal segment with three large, pinnate setae on medial margin, and four small, naked setal elements on distal margin, outer spines 1–3 each with accessory process. Leg 2 (Fig. 31B) as usual for the genus; armature formula I-1; I-1; II, I, 5 for exopod, 0-1; 0-2; 6 for endopod. Leg 3 as Fig. 31C, D. Leg 4 (Fig. 31E) consisting of protopod and two-segmented exopod; protopod with one small seta subdistally; proximal and distal segments of exopod armed with one and three spines, respectively. Leg 5 (Fig. 31F) represented by two papillae; outer and inner papillae tipped with one and two small setae, respectively.

**Figure 32.**
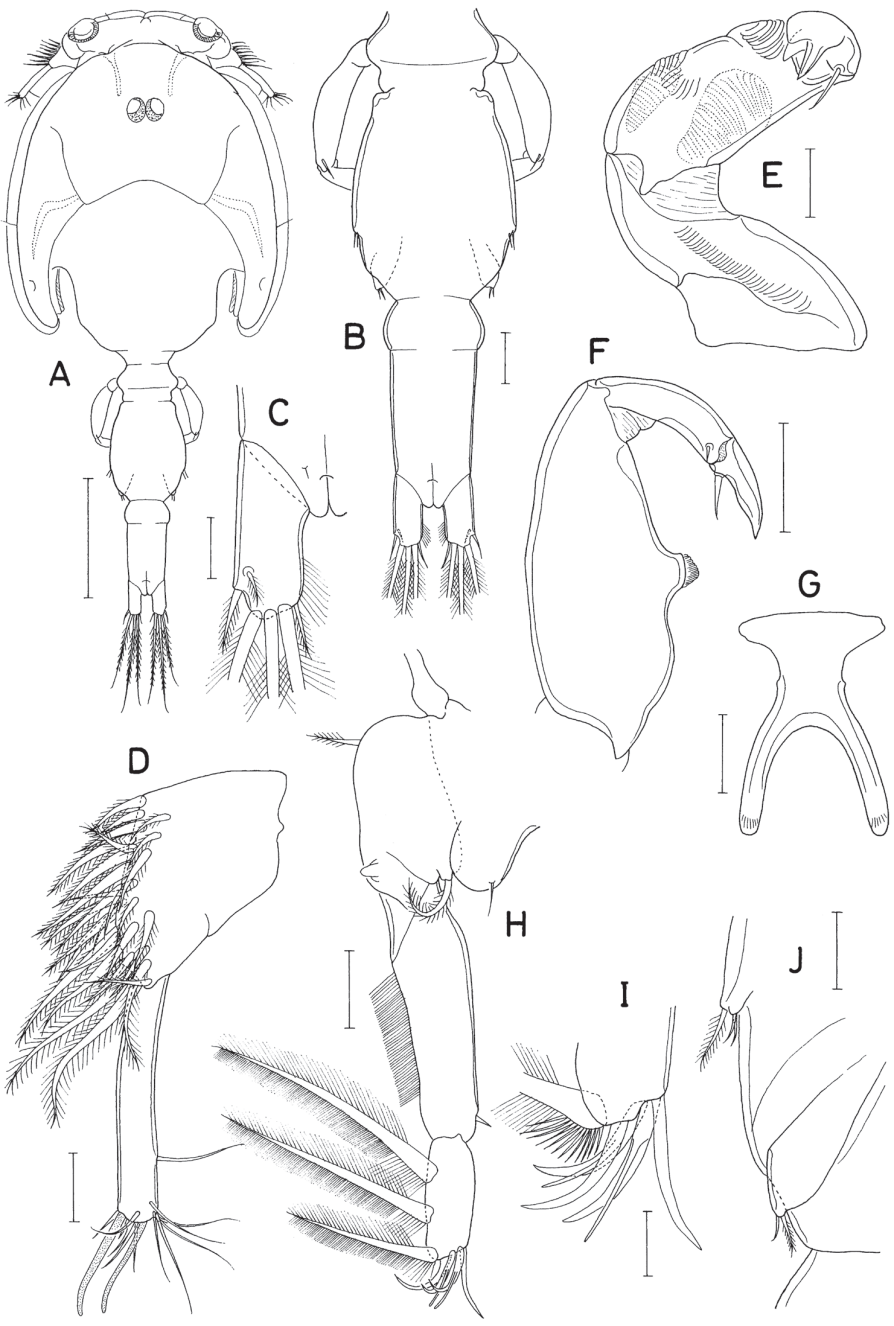
*Caligusamblygenitalis* Shiino, 1961, male **A** habitus, dorsal **B** urosome, dorsal **C** caudal ramus, ventral **D** antennule **E** antenna **F** maxilliped **G** sternal furca **H** leg 1 **I** distal region of leg 1 exopod **J** legs 5 and 6. Scale bars: 0.5 mm (**A**); 0.1 mm (**B, F**); 0.05 mm (**C–E, G, H, J**); 0.02 mm (**I**).

**Male.** Body (Fig. 32A) 2.56 mm long. Urosome (Fig. 32B) indistinctly four-segmented. Fifth pedigerous somite (first urosomal somite) not clearly demarcated from genital complex. Genital complex rhomboidal, 424 × 323 μm. Abdomen indistinctly two-segmented; proximal somite 95 × 195 μm; distal somite 1.63 × longer than wide (273 × 168 μm). Caudal ramus (Fig. 32C) straight backwards, 2.39 × longer than wide (136 × 57 μm).

Antennule (Fig. 32D) armed as in female; proximal segment 172 μm long; distal segment elongated, 184 μm long, longer than proximal segment. Antenna (Fig. 32E) three-segmented; first segment with one corrugated pad; second segment with several corrugated pads; short third segment with one claw-like process, one leaf-like plate and one seta. Postantennary process acutely pointed, larger than that of female. Maxilliped (Fig. 32F) with blunt protrusion tipped with corrugated pad on inner margin. Sternal furca (Fig. 31G) with more slender tines than in female.

Leg 1 (Fig. 32H) different from that of female in absence of spinules on basis, elongate first exopodal segment, and the lack of an accessory process on outer distal spine 1 (Fig. 32I). Legs 2–4 as in female. Leg 5 (Fig. 32J) as in female. Leg 6 (Fig. 32J) represented by two small setae on genital operculum.

###### Remarks.

*Caligusamblygenitalis* was originally described by Pillai (1961) on the basis of a single female specimen from India. Subsequently, Ho and Lin (2003) redescribed this species based on a single female from Taiwan. Previously, Ho and Lin (2001) recorded one female and one male of *C.longipedis* Bassett-Smith, 1898 from Taiwan. However, when Venmathi Maran et al. (2009) redescribed the latter species based on females and males from Penang, Malaysia, they found that the female and the male of Ho and Lin (2001) were not conspecific. A comparison of our Korean material with the above records indicates that the male of Ho and Lin (2001) is not *C.longipedis* but *C.amblygenitalis*. Our female specimens collected by a light trap from Korea are identifiable as young adults, since they are ~ 3.0 mm long, compared to 4.14 mm long in the female of Ho and Lin (2003), and the female genital complex is immature. *Caligusamblygenitalis* is new to the Korean fauna. Both *C.amblygenitalis* and *C.longipedis* belong to the “*C.macarovi*-group” defined by Boxshall (2018).

##### ﻿ Caligus fugu

Taxon classificationAnimaliaSiphonostomatoidaCaligidae

Yamaguti, 1936

F4ADA496-D337-5879-86F3-9B2ED64BB557

###### Material examined.

One ♀, Site 19, 04 Jun. 2020.

###### Remarks.

This species had been placed in the genus *Pseudocaligus*, which is now synonymized with *Caligus* through a molecular analysis (Freeman et al. 2013). *Caligusfugu* is frequently found on the puffer fish *Takifuguniphobles* (Jordan & Snyder, 1901).

##### ﻿ Caligus orientalis

Taxon classificationAnimaliaSiphonostomatoidaCaligidae

Gusev, 1951

72A9864E-0481-58AE-B626-1BCC94CA29D3

###### Material examined.

One ♀, 2 ♂♂, Site 21, 26 May 2017; 1 ♂, Site 25, 06 Jul. 2016; 1 ♂, Site 26, 06 Jul. 2016; 3 ♀♀, 3 ♂♂, Site 27, 09 Jul. 2016; 2 ♀♀, 6 ♂♂, Site 28, 07 Jul. 2016; 4 ♀♀, 1 ♂, Site 29, 09 Jul. 2016; 1 ♀, Site 30, 17 Oct. 2020.

###### Remarks.

*Caligusorientalis* is a parasite of coastal marine and brackish-water fish in the East Asian waters. It has a wide host range and has been reported from over 20 fish species of different orders and families (Nagasawa 2004).

##### ﻿ Caligus punctatus

Taxon classificationAnimaliaSiphonostomatoidaCaligidae

Shiino, 1955

8910C0E6-E9C4-508E-AE86-D977424C5F3C

###### Material examined.

One ♂, Site 15, 04 Jul. 2020; 7 ♀♀, 12 ♂♂, Site 19, 04 Jun. 2020; 4 ♀♀, 7 ♂♂, Site 20, 06 Jun. 2020; 20 ♀♀, 5 ♂♂, Site 22, 31 May 2021.

###### Remarks.

This caligid is common on the gobiid fishes living in brackish-waters in Korea. Kim (1993) studied post-embryonic developmental stages of this species.

##### ﻿ Caligus triangularis

Taxon classificationAnimaliaSiphonostomatoidaCaligidae

Shiino, 1954

BDB2016A-237D-582B-9F93-CAC3B53C5783

###### Material examined.

Two ♀♀, 1 ♂, Site 10, 13 Oct. 2015.

###### Remarks.

The genital complex of the female of this caligid is characteristically triangular. The only known host of this copepod was *Halichoerespoecilopterus* (Richardson, 1846) which has been treated as a junior synonym of *Parajulispoecilepterus* (Temminck & Schlegel, 1845).

##### ﻿ Caligus undulatus

Taxon classificationAnimaliaSiphonostomatoidaCaligidae

Shen & Li, 1959

DC048E6C-F220-5F37-B482-06C0392D6C8B

Fig. 33

###### Material examined.

Four ♀♀, 1 ♂, Site 11, 16 Apr. 2014; 1 ♀, 1 ♂, Site 20, 05 Jun. 2020; 1 ♀, Site 26, 06 Jul. 2016; 1 ♀, 2 ♂♂, Site 31, 15 Nov. 2020.

###### Other material from fish host.

1 ♀ and 1 chalimus from the skin of the fish *Konosiruspunctatus* (Temminck & Schlegel, 1846), at a market at Gonam, Hadong, south coast (34˚59'47"N, 126˚48'37"E), 25 Jul. 2012, leg. I.-H. Kim.

###### Supplementary description of female.

**Figure 33.**
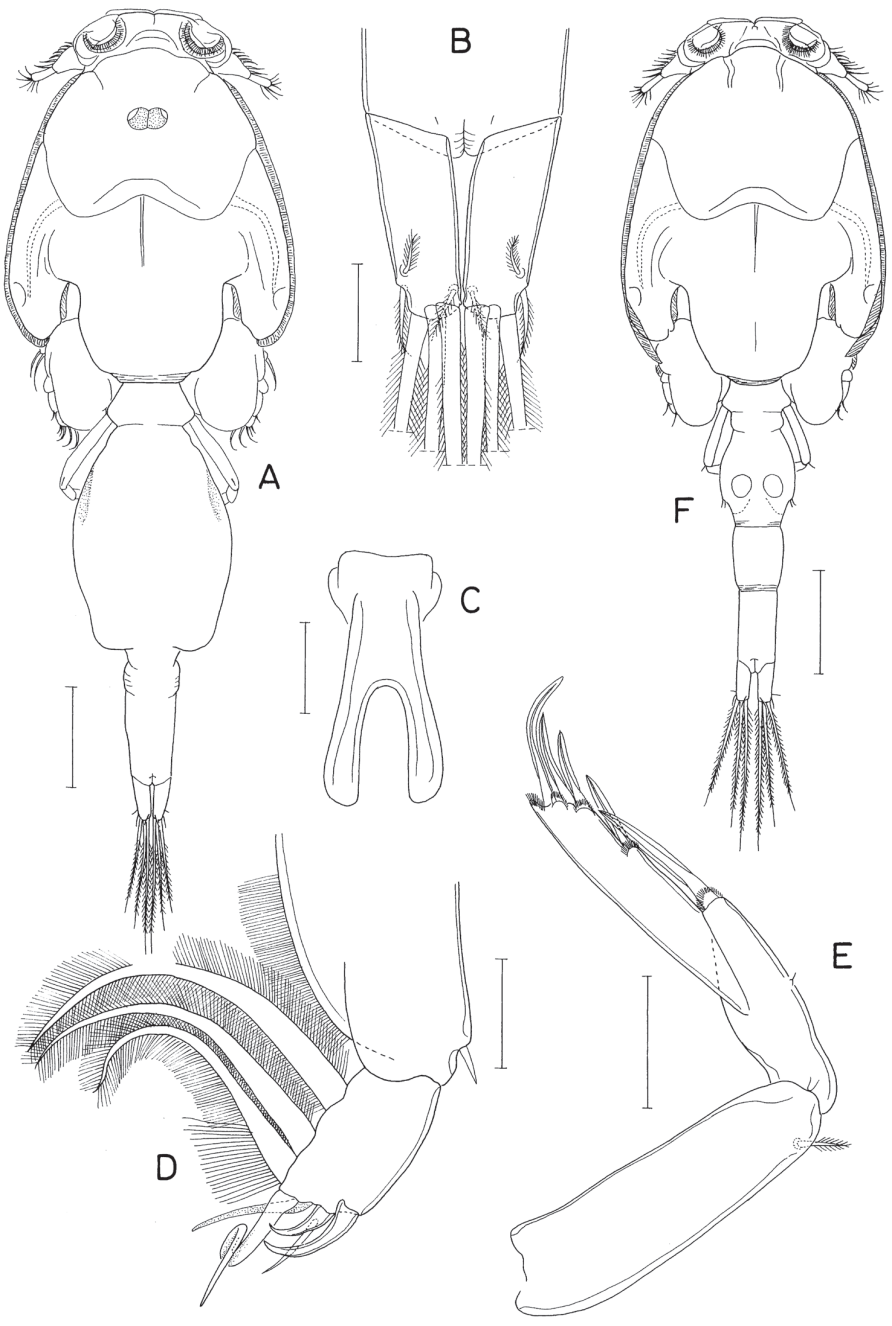
*Caligusundulatus* Shen & Li, 1959 Female **A** habitus, dorsal **B** caudal rami, dorsal **C** sternal furca **D** distal exopodal segment of leg 1 **E** leg 4. Male **F** habitus, dorsal. Scale bars: 0.5 mm (**A, F**); 0.1 mm (**B, C, F**); 0.05 mm (**D**).

Body (Fig. 33A) narrow. Body length 3.92 mm. Cephalothoracic shield distinctly longer than wide (1.76 × 1.36 mm); thoracic zone extending beyond posterior tips of lateral zones. Urosome longer than cephalothoracic shield. Genital complex nearly fusiform, ~ 1.4 × longer than wide (1.07 × 0.77 mm), incompletely articulated from fourth pedigerous somite, truncate posteriorly. Abdomen 0.61 × 0.26 mm, unsegmented, elongate, not articulated from genital complex, with wrinkled cuticle at proximal region. Caudal rami (Fig. 33B) slightly convergent, 2.50 × longer than wide (195 × 78 μm), armed with three large and three small setae; one of small setae positioned ventrally.

Sternal furca (Fig. 33C) narrow; tines gradually narrowed distally, with blunt apex. Distal exopodal segment of leg 1 (Fig. 33D) with three large, pinnate setae on inner margin and four distal armature elements comprising, from outer to inner, claw-like spine 1, smaller claw-like spine 2 bearing accessory process, transparent, aesthetasc-like seta, and long, naked seta. Leg 4 (Fig. 33E) consisting of protopod and two-segmented endopod; protopod with one small seta distally; proximal exopodal segment armed with one spine of 114 μm long; distal exopodal segment armed with four spines of 64, 62, 75, and 101 μm long, respectively, from proximal to distal.

###### Description.

**Male.** Body (Fig. 33F) smaller than that of female, 3.30 mm long. Cephalic shield 1.75 × 1.24 mm. Genital complex longer than wide. Abdomen two-segmented; proximal and distal abdominal somites 310 × 250 μm and 360 × 185 μm, respectively. Caudal ramus 2.57 × longer than wide (185 × 72 μm).

###### Remarks.

*Caligusundulatus* is distributed in tropical and warm waters of the world, and has been frequently found from plankton samples. Moon and Park (2019) also recorded its occurrence in plankton samples from Korea. Ohtsuka et al. (2020) recorded the fish *Sardinellazunasi* (Bleeker, 1854) as a host of *C.undulatus*, which was the first host record. In the present study, we report *Konosiruspunctatus* as an additional host record. The fish hosts *S.zunasi* and *K.punctatus* live on East Asian coasts, sometimes entering bays or brackish waters. It is conceivable that *C.undulatus* and other caligids may detach from the hosts due to the salinity change when the hosts approach brackish waters.

A female specimen from Site 11 exhibited a shrunken genital complex with undulated lateral margins as observed in the type material of Shen and Li (1959). This form of the genital complex may occur immediately after oviposition.

## Supplementary Material

XML Treatment for Anthessius atrinae

XML Treatment for Anthessius graciliunguis

XML Treatment for Conchyliurus quintus

XML Treatment for Hemicyclops japonicus

XML Treatment for Hemicyclops nasutus

XML Treatment for Hemicyclops rapax

XML Treatment for Hemicyclops parilis

XML Treatment for Hersiliodes exiguus

XML Treatment for Pontoclausia cochleata

XML Treatment for Pontoclausia pristina

XML Treatment for Kelleria andamanensis

XML Treatment for Herrmannella dentata

XML Treatment for Herrmannella hoonsooi

XML Treatment for Herrmannella macomae

XML Treatment for Heteranthessius unisetatus

XML Treatment for Modiolicola bifidus

XML Treatment for Pusanomyicola

XML Treatment for Pusanomyicola sensitivus

XML Treatment for Polyankylis ovilaxa

XML Treatment for Polyankylis bogilensis

XML Treatment for Pseudanthessius linguifer

XML Treatment for Critomolgus anthopleurus

XML Treatment for Eupolymniphilus orientalis

XML Treatment for Eupolymniphilus foliatus

XML Treatment for Anchistrotos kojimensis

XML Treatment for Artotrogus acutus

XML Treatment for Ascidipontius rarus

XML Treatment for Bradypontius halocynthiae

XML Treatment for Cryptopontius ascidius

XML Treatment for Cryptopontius donghaensis

XML Treatment for Pteropontius trimerus

XML Treatment for Acontiophorus estivalis

XML Treatment for Dermatomyzon nigripes

XML Treatment for Thermocheres pacificus

XML Treatment for Caligus amblygenitalis

XML Treatment for Caligus fugu

XML Treatment for Caligus orientalis

XML Treatment for Caligus punctatus

XML Treatment for Caligus triangularis

XML Treatment for Caligus undulatus

## References

[B1] Avdeev GV (1987) Two new copepod species (Sabelliphilidae, Poecilostomatoida) from bivalvian molluscs in the Peter the Great Bay of the Japan Sea. Zoologicheskoy Zhurnal 66(4): 608–613.

[B2] Bocquet C Stock JH Bernard F (1959) Copépodes parasites d’invertébrés des côtes de France. IX. Description d’une nouvelle espece remarquable de Lichomolgidae. *Heteranthessiusscotti* n. sp. (Cyclopoida). Proceedings of the Koninklijke Nederlande Akademie van Wetenschappen series c 62(2): 111–118.

[B3] Bocquet C Stock JH Kleeton G (1963) Copépodes parasites d’invertébrés des côtes de la Manche. X. Cyclopoïdes Poecilostomes associés aux Annélides Polychètes, dans la région de Roscoff. Archives de Zoologie Expérimentale et Générale 102(notes et revue 1): 20–40.

[B4] Boxshall G (2018) The sea lice (Copepoda: Caligidae) of Moreton Bay (Queensland, Australia), with descriptions of thirteen new species. Zootaxa 4398(1): 1–172. 10.11646/zootaxa.4398.1.1 29690340

[B5] Boxshall GA Halsey SH (2004) An Introduction to Copepod Diversity. The Ray Society, London, 966 pp.

[B6] Chan BKK Shao K-T Chang Y-W (2016) A simplified, economical, and robust light trap for capturing benthic and pelagic zooplankton. Journal of Experimental Marine Biology and Ecology 482: 25–32. 10.1016/j.jembe.2016.04.003

[B7] Chang CY (2012) First record of monstrilloid copepods in Korea: Description of a new species of the genus *Cymbasoma* (Monstrilloida, Monstrillidae). Animal Systematics, Evolution and Diversity 28(2): 126–132. 10.5635/ASED.2012.28.2.126

[B8] Chang CY (2014) Two new records of monstrilloid copepods (Crustacea) from Korea. Animal Systematics, Evolution and Diversity 30(3): 206–214. 10.5635/ASED.2014.30.3.206

[B9] Chang CY Song SJ (1995) Marine harpacticoid copepods of genus *Eudactylopus* (Harpacticoida, Thalestridae) in Korea. Animal Systematics, Evolution and Diversity 11(3): 379–388.

[B10] Cho DH Wi JH Suh H-L (2018) A new species of *Eudactylopus* (Copepoda: Harpacticoida) from the south coast of Korea based on morphological and molecular evidence. Animal Systematics, Evolution and Diversity 34(3): 127–142. 10.5635/ASED.2018.34.3.127

[B11] Costanzo G Brugnano C Zagami G (2013) A new species of *Eupolymniphilus* (Copepoda: Cyclopoida: Sabelliphilidae) from an anchialine cave of the Mediterranean Sea with a key to the seven species of the genus. Vie et Milieu- Life and Environment 63(2): 75–80.

[B12] Do TT Kajihara T (1984) Two poecilostomatoid copepods, *Anthessiusgraciliunguis* n. sp. and *Modiolicolabifidus* Tanaka, 1961 from the blue mussel, *Mytilusedulisgalloprovincialis* Lamarck, in Japan. Fish Pathology 19(1): 5–15. 10.3147/jsfp.19.5

[B13] Eiselt VJ (1969) Revision von *Acontiophorusantennatus* Hansen 1923 und materialien zur variabilität von Acontiophorus armatus Brady 1880 (Cyclop. Siph., Copepoda, Crust.). Sitzungsberichten der Österreichischen Akademie der Wissenschaften. Mathematisch-Naturwissenschaftliche Klasse 177(8–10): 177–185.

[B14] Freeman MA Anshary H Ogawa K (2013) Multiple gene analyses of caligid copepods indicate that the reduction of a thoracic appendage in *Pseudocaligus* represents convergent evolution. Parasites & Vectors 6(1): 1–9. 10.1186/1756-3305-6-336 24286135 PMC4176263

[B15] Hernandez FJ Shaw RF (2003) Comparison of plankton net and light trap methodologies for sampling larval and juvenile fishes at offshore petroleum platforms and a coastal jetty off Louisiana. American Fisheries Society Symposium 36: 15–38.

[B16] Ho J-S Kim I-H (1997) A new family of poecilostomatoid copepods (Polyankyliidae) from a tide pool on mud flat in Korea. Korean Journal of Biological Sciences 1: 429–434.

[B17] Ho J-S Kim I-H (2003) New clausiid copepods (Poecilostomatoida) associated with polychaetes of Korea, with cladistic analysis of the family Clausiidae. Journal of Crustacean Biology 23(3): 568–581. 10.1651/C-2370

[B18] Ho J-S Lin C-L (2001) Sea lice (Copepoda, Caligidae) parasitic on carangid fishes of Taiwan. Taiwan Shuichanxue Hui Kan 28(3): 177–201.

[B19] Ho J-S Lin C-L (2003) Three species of *Caligus* (Copepoda: Caligidae) parasitic on fishes of the northeast coast of Taiwan. Taiwan Shuichanxue Hui Kan 30(1): 55–70.

[B20] Holmes JMC (1985) *Anchistrotoslucipetus* sp. nov. (Copepoda, Taeniacanthidae), a parasitic copepod From Lough Ine, South West Ireland. Crustaceana 48(1): 18–25. 10.1163/156854085X00684

[B21] Hong J-S Kim I-H (2021) Copepods of the family Kelleriidae from tropical waters (Crustacea, Copepoda, Cyclopoida). Journal of Species Research 10(4): 364–386. 10.12651/JSR.2021.10.4.364

[B22] Humes AG (1986) *Myicolametisiensis* (Copepoda: Poecilostomatoida), a parasite of the bivalve *Myaarenaria* in eastern Canada, redefinition of the Myicolidae, and diagnosis of the Anthessiidae n. fam. Canadian Journal of Zoology 64(4): 1021–1033. 10.1139/z86-152

[B23] Jeon D Lee W Suh HY (2018) A new genus and two new species of monstrilloid copepods (Copepoda: Monstrillidae): integrating morphological, molecular phylogenetic, and ecological evidence. Journal of Crustacean Biology 38(1): 45–65. 10.1093/jcbiol/rux095

[B24] Jeon D Lee W Soh HY (2019) New species of *Caromiobenella* Jeon, Lee & Soh, 2018 (Crustacea, Copepoda, Monstrilloida) from Chuja Island, Korea. ZooKeys 814: 33–51. 10.3897/zookeys.814.29126 PMC6333727 30651711

[B25] Karanovic T (2008) Marine interstitial Poecilostomatoida and Cyclopoida (Copepoda) of Australia. Crustaceana Monographs 9. Brill, Leiden, 331 pp. 10.1163/ej.9789004164598.i-332

[B26] Kim I-H (1993) Developmental stages of *Caliguspunctatus* Shiino, 1955 (Copepoda: Caligidae). In: Boxshall GA Defaye D (Eds) Pathogens of Wild and Farmed Fish: Sea Lice.Ellis Horwood, New York, etc., 16–29.

[B27] Kim I-H (1998) Illustrated Encyclopedia of Fauna & Flora of Korea. Vol. 38. Cirripedia, Symbiotic Copepoda, Pycnogonida. Ministry of Education, Korea, 1038 pp.

[B28] Kim I-H (2003) Copepodid stages of *Critomolgusanthopleurus* (Copepoda, Poecilostomatoida, Rhynchomolgidae). Journal of Crustacean Biology 23(3): 558–567. 10.1651/C-2375

[B29] Kim I-H (2004) Copepods associated with bivalves in Korea and their distribution. Zoological Studies 43(2): 187–192.

[B30] Kim I-H (2009) Poecilostome copepods (Crustacea: Cyclopoida) associated with marine invertebrates from tropical waters. Korean Journal of Systematic Zoology 7(Special Issue): 1–90.

[B31] Kim I-H (2010a) Invertebrate fauna of Korea, Vol. 21, No. 5. Symbiotic copepods. Flora and Fauna of Korea, National Institute of Biological Resources, Ministry of Environment, Korea, 222 pp.

[B32] Kim I-H (2010b) Siphonostomatoid Copepoda (Crustacea) associated with invertebrates from tropical waters. Korean Journal of Systematic Zoology 8(Special Issue): 1–176.

[B33] Kim I-H (2014) Six new species of Copepoda (Clausiidae, Pseudanthessiidae, Polyankyliidae) associated with polychaetes from Korea. Journal of Species Research 3(2): 95–122. 10.12651/JSR.2014.3.2.095

[B34] Kim I-H Hong J-S (2014) Copepods (Crustacea, Copepoda, Cyclopoida) associated with marine invertebrates from Thailand. Animal Systematics, Evolution and Diversity 30(4): 274–318. 10.5635/ASED.2014.30.4.274

[B35] Kim I-H Sato SI (2010) A review of copepods associated with bivalves in Japan, with description of two new species (Crustacea, Copepoda, Cyclopoida). Bulletin of the Tohoku University Museum 9: 1–22.

[B36] Kim I-H Stock JH (1996) A new species of Clausidiidae (Copepoda, Poecilostomatoida) associated with the bivalve *Ruditapesphilippinarum* in Korea. Cahiers de Biologie Marine 37(1): 1–6.

[B37] Lee J Chang CY (2016) A new species of *Monstrilla* Dana, 1849 (Copepoda: Monstrilloida: Monstrillidae) from Korea, including a key to species from the north-west Pacific. Zootaxa 4174(1): 396–409. 10.11646/zootaxa.4174.1.24 27811807

[B38] Lee J Kim D Chang CY (2016) Two new species of the genus *Monstrillopsis* Sars, 1921 (Copepoda: Monstrilloida: Monstrillidae) from South Korea. Zootaxa 4174(1): 410–423. 10.11646/zootaxa.4174.1.25 27811808

[B39] López-González PJ Conradi M (1995) *Heteranthessiushoi*, a new species (Copepoda: Pseudanthessiidae) from a sea-anemone in the Straits of Gibraltar, with remarks on the genus. Proceedings of the Biological Society of Washington 108(1): 107–116.

[B40] McLeod LE Costello MJ (2017) Light traps for sampling marine biodiversity. Helgoland Marine Research 71(1): e2. 10.1186/s10152-017-0483-1

[B41] Moon SY Kim I-H (2010) Three new species of *Hemicyclops* (Copepoda, Cyclopoida, Clausidiidae) from Korea. Korean Journal of Systematic Zoology 26(3): 279–293. 10.5635/KJSZ.2010.26.3.279

[B42] Moon SY Park JS (2019) Occurrence of sea lice, *Caligusundulatus* Shen and Li, 1959 (Copepoda: Siphonostomatoida: Caligidae) in plankton samples collected from Korea. Journal of Species Research 8(4): 365–372. 10.12651/JSR.2019.8.4.36

[B43] Nagasawa K (2004) Sea lice, *Lepeophtheirussalmonis* and *Caligusorientalis* (Copepoda: Caligidae), of wild and farmed fish in sea and brackish waters of Japan and adjacent regions: a review. Zoological Studies 43(2): 173–178.

[B44] Nicholls AG (1944) Littoral Copepoda from South Australia (II) Calanoida, Cyclopoida, Notodelphyoida, Monstrilloida and Caligoida. Records of the South Australian Museum 8: 1–62.

[B45] Ohtsuka S Nawata M Nishida Y Nitta M Hirano K Adachi K Kondo Y Venmathi Maran BA Suárez-Morales E (2020) Discovery of the fish host of the ‘planktonic’ caligid *Caligusundulatus* Shen & Li, 1959 (Crustacea: Copepoda: Siphonostomatoida). Biodiversity Data Journal 8: e52271. 10.3897/BDJ.8.e52271 PMC7295818 32565681

[B46] Øresland V (2007) Description of the IMR standard light trap and the vertical distribution of some decapod larvae (Homarus and Nephrops). Western Indian Ocean Journal of Marine Science 6(2): 225–231. 10.4314/wiojms.v6i2.48249

[B47] Pillai NK (1961) Copepods parasitic on South Indian fishes. Part. 1, Caligidae. Bulletin of the Research Institute. University of Kerala 8: 87–130.

[B48] Porter SS Eckert GL Byron CJ Fosher JL (2008) Comparison of light traps and plankton tows for sampling brachyuran crab larvae in an Alaskan Fjord. Journal of Crustacean Biology 28(1): 175–179. 10.1651/06-2818R.1

[B49] Sars GO (1915) CopepodaCyclopoida. Parts IX & X. Ascomyzontidae (concluded), Acontiophoridae, Myzopontiidae, Dyspontiidae, Artotrogidae, Cancerillidae. An account of the Crustacea of Norway with short descriptions and figures of all the species. Bergen Museum, Bergen 6: 105–140.

[B50] Sars GO (1918) CopepodaCyclopoida. Parts XIII & XIV. Lichomolgidae (concluded), Oncaeidae, Corycaeidae, Ergasilidae, Clausiidae, Eunicicolidae, Supplement. An account of the Crustacea of Norway with short descriptions and figures of all the species. Bergen Museum, Bergen 6: 172–225.

[B51] Scott T (1903) On some new and rare Crustacea collected at various times in connection with the investigations of the Fisheries board for Scotland. Twenty-first Annual Report of the Fishery Board for Scotland 21(3): 109–135.

[B52] Shen CJ Li HL (1959) Parasitic copepods from fishes of China, IV. Caligoida. Caligidae (3). Acta Zoological Sinica 11(1): 12–23.

[B53] Sigurdsson GM Morse B Rochette R (2014) Light traps as a tool to sample pelagic larvae of American lobster (*Homarusamericanus*). Journal of Crustacean Biology 34(2): 182–188. 10.1163/1937240X-00002219

[B54] Stock JH (1954) Redescription de *Tococherescylindraceus* Pelseneer, 1929, copépode commensal de Loripes lacteus. Beaufortia 4(38): 73–80.

[B55] Stock JH (1971) Découverte du genre Heteranthessius (Copepoda) on Méditerranée: *H.furcatus* n. sp. Bulletin de la Société Zoologique de France 95: 335–340.

[B56] Stock JH Humes AG Gooding RU (1964) Copepoda associated with West Indian invertebrates. IV. The genera *Octopicola*, *Pseudanthessius* and *Meomicola* (Cyclopoida, Lichomolgidae). Studies on the Fauna of Curaçao 18(77): 1–74.

[B57] Ueda H Nagai H Hibino M Tanaka M (2006) Redescription of a symbiotic poecilostomatoid copepod *Anthessiusgraciliunguis* Do & Kajihara from plankton: The second record of the species and first record of the male. Plankton & Benthos Research 1(2): 102–108. 10.3800/pbr.1.102

[B58] Venmathi Maran BA Seng Ohtsuka S Nagasawa K (2009) Records of *Caligus* (Crustacea: Copepoda: Caligidae) from marine fish cultured in floating cages in Malaysia with a redescription of the male of *Caliguslongipedis* Bassett-Smith, 1898. Zoological Studies 48(6): 797–807.

[B59] WoRMS Editorial Board (2021) World Register of Marine Species. http://www.marinespecies.org [accessed 1 November 2021]

